# Interlaboratory study to validate a STR profiling method for intraspecies identification of mouse cell lines

**DOI:** 10.1371/journal.pone.0218412

**Published:** 2019-06-20

**Authors:** Jamie L. Almeida, Aleksandra Dakic, Karin Kindig, Maikan Kone, Deborah L. D. Letham, Scott Langdon, Ruth Peat, Jayamalini Holding-Pillai, Erin M. Hall, Mark Ladd, Megan D. Shaffer, Heath Berg, Jinliang Li, Georges Wigger, Steve Lund, Carolyn R. Steffen, Barbara B. Fransway, Bob Geraghty, Manuela Natoli, Beth Bauer, Susanne M. Gollin, Dale W. Lewis, Yvonne Reid

**Affiliations:** 1 National Institute of Standards and Technology, Biosystems and Biomaterials Division, Gaithersburg, Maryland, United States of America; 2 ATCC, Standards Resource Center, Manassas, Virginia, United States of America; 3 Charles River Laboratories, Malvern, Pennsylvania, United States of America; 4 Duke University, Durham, North Carolina, United States of America; 5 The Francis Crick Institute, Cell Services, London, United Kingdom; 6 Genetica Cell Line Testing –a LabCorp brand, Burlington, North Carolina, United States of America; 7 IDEXX BioAnalytics, Columbia, Missouri, United States of America; 8 Laragen Inc., Culver City, California, United States of America; 9 Microsynth AG, Balgach, Switzerland; 10 National Institute of Standards and Technology, Statistical Engineering Division, Gaithersburg, Maryland, United States of America; 11 National Institute of Standards and Technology, Biomolecular Measurement Division, Gaithersburg, Maryland, United States of America; 12 University of Arizona, Genetics Core Lab, University of Arizona Genetics Core, Tucson, Arizona, United States of America; 13 University of Cambridge, Cancer Research UK, Cambridge Institute, Cambridge, United Kingdom; 14 University of Maryland, College Park, Maryland; 15 University of Pittsburgh, Department of Human Genetics, Pittsburgh, Pennsylvania, United States of America; Suez Canal University Faculty of Medicine, EGYPT

## Abstract

The Consortium for Mouse Cell Line Authentication was formed to validate Short Tandem Repeat (STR) markers for intraspecies identification of mouse cell lines. The STR profiling method is a multiplex polymerase chain reaction (PCR) assay comprised of primers targeting 19 mouse STR markers and two human STR markers (for interspecies contamination screening). The goals of the Consortium were to perform an interlaboratory study to–(1) validate the mouse STR markers to uniquely identify mouse cell lines (intraspecies identification), (2) to provide a public database of mouse cell lines with the National Institute of Standards and Technology (NIST)-validated mouse STR profiles, and (3) to publish the results of the interlaboratory study. The interlaboratory study was an international effort that consisted of 12 participating laboratories representing institutions from academia, industry, biological resource centers, and government. The study was based on 50 of the most commonly used mouse cell lines obtained from the American Type Culture Collection (ATCC). Of the 50 mouse cell lines, 18 had unique STR profiles that were 100% concordant (match) among all Consortium laboratory members, and the remaining 32 cell lines had discordance that was resolved readily and led to improvement of the assay. The discordance was due to low signal and interpretation issues involving artifacts and genotyping errors. Although the total number of discordant STR profiles was relatively high in this study, the percent of labs agreeing on allele calls among the discordant samples was above 92%. The STR profiles, including electropherogram images, for NIST-validated mouse cell lines will be published on the NCBI BioSample Database (https://www.ncbi.nlm.nih.gov/biosample/). Overall, the interlaboratory study showed that the multiplex PCR method using 18 of the 19 mouse STR markers is capable of discriminating at the intraspecies level between mouse cell lines. Further studies are ongoing to refine the assay including (1) development of an allelic ladder for improving the accuracy of allele calling and (2) integration of stutter filters to identify true stutter.

## Introduction

The impact of misidentification and cross-contamination of animal cell lines in the scientific community continues to be a serious problem [1, 2]. Although some journals require cell line authentication prior to publication [2], others only recommend it, allowing for non-validated materials and possible misinformation to enter the public domain. The scientific community and funding agencies are encouraging data transparency to improve reproducibility and the quality of research (https://grants.nih.gov/reproducibility/index.htm) [3–6]. One aspect of reproducibility is validating the biological reagents used in experiments to include cell lines which continue to be the main work horse in biological research. For human cell lines there are validated methods [7], kits, and databases available for intraspecies identity testing. For non-human cell lines there are validated methods for interspecies identification such as DNA barcoding [8] and the species-specific multiplex PCR assay [9] (ASN-0004: Species-Level Identification and Cross-Contamination Screening in Animal Cells by Multiplex PCR–Working group in progress); however, these methods are unable to distinguish between non-human cell lines of the same species.

Mouse models have been an important tool in biomedical research, especially with the advancement of transgenic mouse technologies, such as the CRISPR/Cas9 system [10]. With the increasing number of transgenic systems and potential new cell lines created, there is a need for intraspecies identification of mouse cell lines. Indeed, mouse cell lines are the second most frequently used cell lines in basic research when compared to human cell lines. The Mouse Cell Line Authentication Consortium was formed to validate a short tandem repeat (STR) multiplex PCR assay to distinguish among mouse cell lines. This assay improves upon the patented methods (US Patent 9,556,482 B2) previously described [11], which now includes additional primers that target a total of 19 mouse STR loci and two human STR markers for contamination detection (US patent application publication 2017/0101677 A1). This method is rapid, inexpensive, and uses related methods and instrumentation already in place for human cell line authentication by STR profiling. This validated method is the first step in authenticating a mouse cell line by determining its STR profile identity.

In October of 2016, a Federal Register Notice (FRN) was published inviting eligible laboratories to join the Mouse Cell Line Authentication Consortium (https://www.federalregister.gov; 81 FR 70665). Eleven laboratories responded to the FRN and formally signed a Cooperative Research and Development Agreement (CRADA) with NIST, the 12^th^ laboratory, to join the Consortium (see Table 1). Test kits were distributed to Consortium laboratory members; each kit contained DNA isolated from 50 mouse cell lines (coded samples), primers, PCR Master Mix, calibrants, and control human DNA. Each Consortium laboratory member was provided a detailed protocol and asked to perform the assay as written. The protocol described how to amplify the STR target regions of the mouse DNA from the 50 cell lines using PCR, separate the amplicons using capillary electrophoresis (CE), size the fragments based on calibrants, and submit the exported data file to NIST. NIST analyzed the data and submitted comments to the Consortium members. Consortium members met on several occasions via webinar and teleconference calls to discuss the interpretation and analysis of the submitted data.

**Table 1:** Consortium laboratory members.

Affiliation	Institution	Country
ATCC	Cell Bank	USA
Charles River Laboratories	Industry	USA
Duke University	Academia	USA
Francis Crick Institute	Academia	UK
Genetica Cell Line Testing	Industry	USA
IDEXX BioAnalytics	Industry	USA
Laragen Inc.	Industry	USA
Microsynth AG	Industry	Switzerland
NIST	Government	USA
University of Arizona	Academia	USA
University of Cambridge	Academia	UK
University of Pittsburgh	Academia	USA

This multiplex PCR assay provides a unique STR profile for each mouse cell line tested, including closely related cell lines. Moreover, cell lines from within a mouse strain showed conserved alleles at particular loci that can be used to identify cell lines at the strain level, enabling the detection of misidentified cell lines. Here we describe the results of the study in detail and the path forward.

## Results

### Initial data analysis

Upon observation of the first set of data submitted to NIST, it was apparent that interpretation of data was inconsistent among the Consortium laboratory members primarily due to PCR artifacts, and to a lesser extent, low signal and genotyping (bin) errors. PCR artifacts, mainly stutter, were a major factor in the discordant allele calls. The electropherograms for each cell line were very similar among Consortium laboratory members, but interpretation of allele calls, most notably peaks in the +4 and -4 stutter positions, varied. Stutter is a PCR artifact that is caused by strand slippage of the polymerase enzyme during amplification resulting in a small peak that occurs either one repeat before or after the true allele at a STR marker [12, 13]. The STR loci targeted in this assay are tetranucleotide repeats; therefore, the stutter appears either four base pairs before or after the true allele. Traditionally, stutter filters are used in fragment analysis software to flag a peak in the stutter position as being a potential allele or an artifact. Since stutter filters were not yet established for the mouse cell line STR profiling assay, some Consortium laboratory members relied on default stutter filters validated for human cell lines in their software to call alleles for each sample. Consequently, some alleles were missed due to the lack of pre-established stutter filters.

### Data analysis using stutter rules

Since stutter filters for the mouse cell line STR profiling assay were not yet established, the Consortium laboratory members in a consensus pronouncement, determined the stutter filter settings based on the data. The stutter ratios were calculated based on the following rules: (1) any peak in the -4 stutter position with a stutter ratio greater than 20% would be called an allele, and (2) any peak in the +4 stutter position with a stutter ratio above 10% would be called an allele. The Consortium laboratory members were also asked to ensure that each allele was properly labeled since the exported data file only included information for peaks that were assigned an allele call. Using these guidelines, the Consortium achieved full concordance in 18 out of 50 mouse cell lines (see Concordance section). A large part of discordance was due to variations in data interpretation, specifically the lack of stutter ratio calculations, and in some cases low instrument signal. Consortium laboratory members were asked to provide reasons for discordance including errors in stutter ratio calculations, artifacts present, or low signal. These determinations led to corrections in final allele calls. After establishing rules and offering several training webinars, the consensus allele calls among the Consortium laboratory members improved to over 92% (see Concordance section under Results).

### Stutter ratio comparison across laboratories

**Fig 1:**
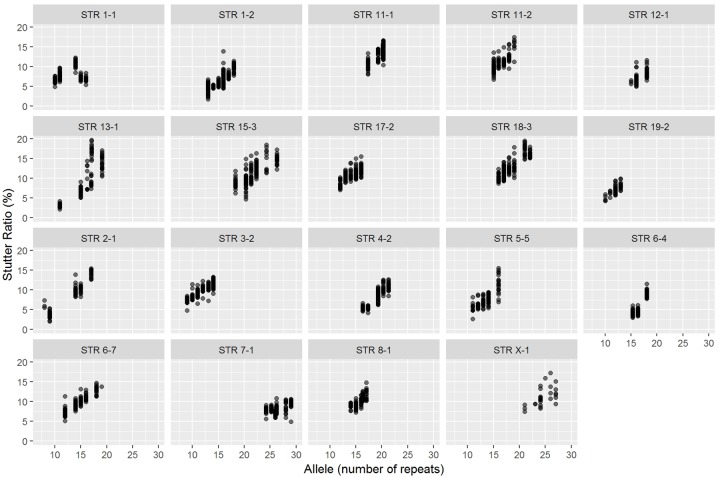
Stutter ratios calculated from diploid mouse DNA samples. Each marker with associated stutter ratios for the represented alleles is plotted here. The y-axis is the observed percent stutter ratio and x-axis shows the allele number (number of repeats).

To determine if stutter ratios vary across the different instrument platforms in the interlaboratory study, stutter ratios were analyzed in DNA samples from normal mouse tissue (diploid) for each represented allele in the calibrant samples (C1 though C5, see Materials and methods) from all Consortium laboratory members. As seen in literature, stutter ratios increased with increasing number of uninterrupted repeats (see Fig 1), except in some microvariant alleles where the longest uninterrupted repeat is shorter than what was found in the full repeat [14].

**Fig 2:**
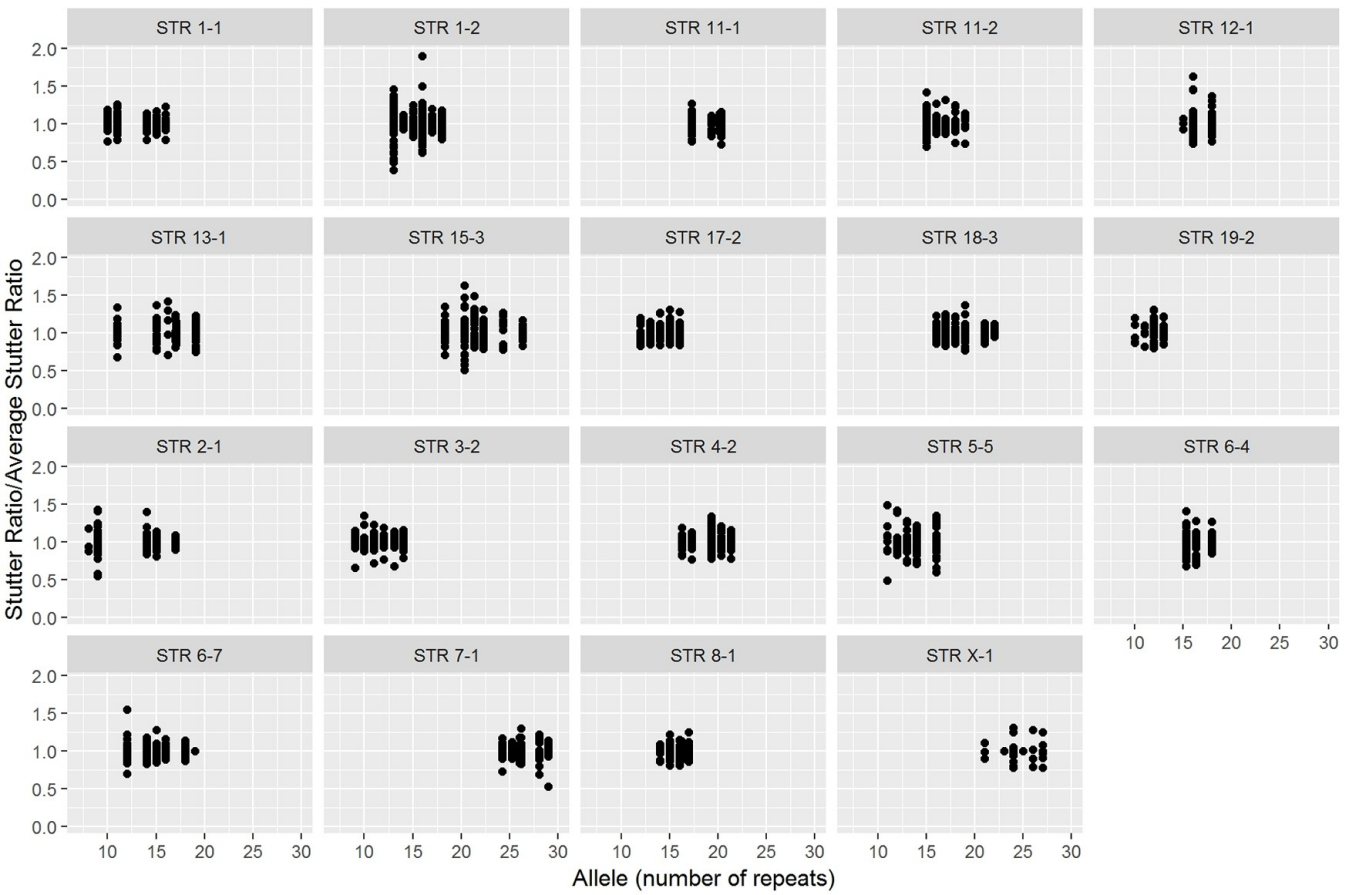
Normalizing stutter across alleles. The y-axis shows each observed stutter ratio divided by the average of all stutter ratios observed for the same marker and allele. The x-axis shows the allele number (number of repeats).

The stutter ratios across alleles were then normalized for repeat length, since this was the most prominent source of variation. That is, each observed stutter ratio was divided by the average of all stutter ratios observed for the same marker and allele combination (see Fig 2).

**Fig 3:**
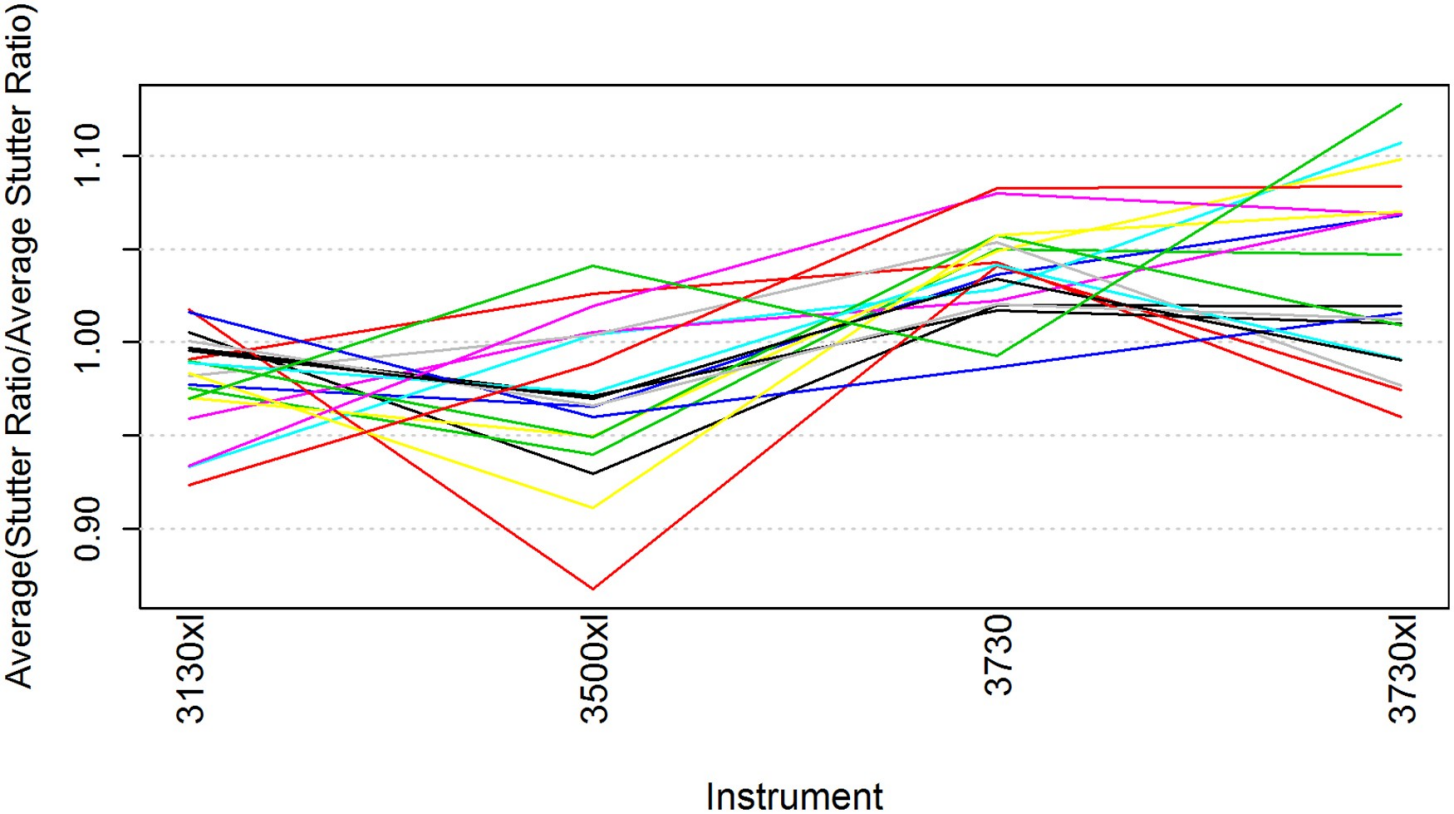
Summary of the effect of instrument on stutter ratio across markers. The combination across the 19 STR markers and relative position for each of the instruments is condensed into a single plot. Each line corresponds to a particular marker, where the four points on each line denote the respective average normalized stutter ratios within that STR marker for the four instruments.

To see a trend or pattern across the relationships of the instruments, the average normalized stutter ratio was computed for each instrument within each marker. The Friedman test [15], an analysis looking for trends among ranks, was applied and showed that there was a statistically significant difference between the instruments (see Fig 3). Stutter ratios from either the ABI 3130xl or 3500xl instruments were found to be roughly 1% lower on average than stutter ratios from either the ABI 3730 and 3730xl instruments. Although statistically significant, the difference is negligible from a practical perspective, as it is roughly the same size as variability in stutter ratios observed from one CE run to another on different days using the same instrument and running the same sample.

### Stutter filter recommendations determined from this study

Stutter filters for the mouse multiplex PCR assay were not available during the interlaboratory study. However, data from the study were used to create stutter filter recommendations for future studies. The stutter ratios calculated are allele-specific (see S1 Table). Currently, most available software only allows the addition of marker-specific stutter filters. For this study, marker-specific stutter was calculated across represented alleles from normal, diploid mouse DNA by taking the mean of the stutter ratios for all alleles at a specific locus and three standard deviations were added (see Table 2). This method has been used previously for marker-specific stutter filters for human STRs [16]. As seen in Table 2, there are two loci (18–3 and 13–1) that have stutter filter recommendations above the 20% stutter threshold that was used to make allele calls in this study. Only DNA from normal, diploid samples was used in calculating stutter ratios. Therefore, some alleles that are present in cancer cell lines may not be represented in the marker-specific stutter recommendations. Application of this recommendation led to consistent allele calls.

**Table 2:** Marker-specific stutter filter recommendations.

STR Marker	Known Allele Range	Stutter Ratio (%)	Allele Range Represented in Stutter Ratio Calculation
18–3	13–28	20.52	16–23
4–2	13–23.3	13.80	16.3–22.3
11–1	17–23.3	19.75	17.3–23.3
6–7	11–26	16.13	12–26
19–2	10–16	11.77	10–16
1–2	12–30.1	13.20	13–22
7–1	19.2–33.2	12.98	24.2–33.2
1–1	10–19	13.71	10–20
3–2	9–25	16.07	9–25
8–1	6–19	18.12	6–18.3
2–1	8–17.1	17.71	9–16
15–3	12–31.3	19.03	18.3–27.3
6–4	12.2–21	12.16	15.3–21
11–2	12–26.2	17.16	15–26.2
17–2	11–22	16.98	12–22
12–1	15–24.1	11.90	15–23
5–5	11–22	13.72	11–21
X-1	15.2–37	18.74	21–37
13–1	11–20.1	22.60	11–20.1

The allele-specific stutter filter would be useful in determining allele calls for these markers, especially at the lower repeat number (allele) that have a much smaller associated percent stutter. In the future, stutter filters will be incorporated into the data analysis software program and will be used to flag alleles that need to be examined more closely. If the sample is composed of a mixture of cell lines, then stutter filters cannot be applied to the data set, since multiple contributors may have varying peak heights in the profile that can invalidate the stutter filters. It is important to note that not all alleles were represented in the stutter filter calculations.

### Signal intensity

Signal intensity was appropriate to allow for genotyping of the cell lines. The signals were above threshold limits and accurate allele calls were made for a nominal starting amount of 2 ng of DNA. Accurate quantitation to determine DNA concentration is important for optimal signal intensity (see Methods section). In few cases, low signal was observed in the red dye channel, the lowest fluorescence intensity of all the dyes, for markers X-1 and 13–1. This may be due to a lower DNA amount resulting from loss of DNA to the walls of the tube or pipetting error. The low signal can be remedied by increasing the amount of DNA in the PCR reaction mix, changing the injection time/voltage during CE, or increasing the number of cycles during PCR.

### Genotyping (Bin)

Genotyping is the determination of allele calls among STR markers. In order to make accurate allele calls or genotyping, bins were created. Bins are place holders in the software for converting imprecise DNA fragments into alleles that have been determined based on sequenced calibrants (refer to S2 and S3 Files for protocol information regarding establishing bins in the different software platforms). Calibrants, which are sequenced diploid DNA extracts from known mouse strains, were used to adjust bins, optimize the assay on the different instrument platforms (see Table 3), and to aid in accurate allele calling (reference S2 and S3 Files). Optimized protocols for capillary electrophoresis were determined by each Consortium laboratory member and the parameters are described in Table 4.

**Table 3:** Consortium laboratory member instrument, polymer, array, and software used in the interlaboratory study.

Group	Instrument	Array	Polymer	Software
1	ABI 3130xl	36 cm	POP-4	GeneMapper v5.0
2	ABI 3130xl	50 cm	POP-7	GeneMapper v5.0
3	ABI 3130xl	36 cm	POP-4	GeneMapper ID v3.2.1
4	ABI 3130xl	50 cm	POP-7	GeneMapper v5.0
5	ABI 3730xl	50 cm	POP-7	GeneMapper 4.0
6	ABI 3130xl	36 cm	POP-4	GeneMapper v4.0
7	ABI 3730xl	50 cm	POP-7	GeneMapper v4.0, v5.0
8	ABI 3730xl	50 cm	POP-7	Gene Marker v2.7.6
9	ABI 3130xl	36 cm	POP-4	GeneMapper v4.1, v5.0
10	ABI 3730	36 cm	POP-7	GeneMarker v1.85, v2.6.3
11	ABI 3500xl	50 cm	POP-7	GeneMapper ID-X v1.2
12	ABI 3500xl	36 cm	POP-4	GeneMapper ID-X v1.3

**Table 4:** Parameters for capillary electrophoresis for each group.

Group	CE Parameters
**1, 9, 12**	**ABI 3500xl, ABI3130xl, 36cm array using POP-4**: oven temp (60 °C), run time (1500 sec), run voltage (15 kV), pre-run time (180 sec), pre-run voltage (15 kV), injection time (15 sec), injection voltage (1.2 kV), and data delay (1 sec).
**11**	**ABI 3500xl 50cm array with POP-7**: oven temp (60 °C), run time (2000 sec), run voltage (15 kV), pre-run time (180 sec), pre-run voltage (15 kV), injection time (15 sec), injection voltage (1.2 kV), and data delay (1 sec).
**2**	**ABI 3130xl 50cm array with POP-7**: oven temp (60 °C), run time (6000 sec), run voltage (15kV), pre-run time (180 sec), pre-run voltage (15 kV), injection time (15 sec), injection voltage (1.6 kV), and data delay (200 sec).
**4**	**ABI 3130xl 50cm array with POP-4**: oven temp (60 °C), run time (2200 sec), run voltage (15 kV), pre-run time (180 sec), pre-run voltage (15 kV), injection time (15 sec), injection voltage (1.6 kV), and data delay (500 sec).
**3, 6**	**ABI 3130xl 36cm array with POP-4**: oven temp (60 °C), run time (1500–2700 sec), run voltage (15 kV), pre-run time (180 sec), pre-run voltage (15 kV), injection time (10 sec), injection voltage (3 kV), and data delay (1 sec).
**7**	**ABI 3730xl 50cm array with POP-7**: oven temp (63 °C), run time (5000 sec), run voltage (10 kV), pre-run time (180 sec), pre-run voltage (15 kV), injection time (25 sec), injection voltage (1.6 kV), and data delay (NA).
**8**	**ABI 3730xl 50cm array with POP-7**: oven temp (63 °C), run time (2100 sec), run voltage (15 kV), pre-run time (180 sec), pre-run voltage (15 kV), injection time (10 sec), injection voltage (1.6 kV), and data delay (500 sec).
**5**	**ABI 3730xl 50cm array with POP-7**: oven temp (60 °C), run time (3000 sec), run voltage (15 kV), pre-run time (180 sec), pre-run voltage (15 kV), injection time (15 sec), injection voltage (1.2 kV), and data delay (1 sec).
**10**	**ABI 3730 36cm array with POP-7**: oven temp (66 °C), run time (1200 sec), run voltage (15 kV), pre-run time (180 sec), pre-run voltage (15 kV), injection time (10 sec), injection voltage (2 kV), and data delay (120 sec).

The calibrants covered 111 alleles (67%) of the 165 alleles observed in the 50 mouse cell lines. There was sufficient allele coverage by the calibrants for accurate allele calls of 49 of the 50 cell lines tested; consequently, identifying alleles in the test samples using extrapolation of alleles in the calibrants was successful. However, there was one exception for cell line C1498, when extrapolation of calibrants was unsuccessful for STR marker 18–3. Allele 28, at STR marker 18–3, fell outside the range of the calibrant at allele 22. Sanger sequencing at marker 18–3 confirmed the allele had 28 repeats. Increased coverage of alleles would improve allele calls with the application of an allelic ladder. These calibrants used for genotyping are not a renewable source. NIST and ATCC are currently developing an allelic ladder for the mouse STR markers.

### Human cell line control sample

The human diploid palatal mesenchymal cell line, HEPM, was used as a control to detect contamination of human cells by confirming the presence of primers for two human STR markers, D8S1106 and D4S2408. The STR markers were incorporated in the multiplex PCR assay to test for human contamination of mouse cell lines. The electropherogram for the human control DNA obtained from HEPM is shown in S1 Fig. All human control samples resulted in amplified products exclusively sized in the human STR regions. In this study, no human cell line contamination was observed in the fifty mouse cell lines tested. Previous studies described sensitivity of the human STR markers [11] in an assay targeting nine mouse STR loci with levels of human contamination detected as low as 62 pg of DNA. Similar results were found when DNA from HEPM cells were tested with the additional mouse STR markers used in this interlaboratory study.

### Removal of STR marker 11–1

**Fig 4:**
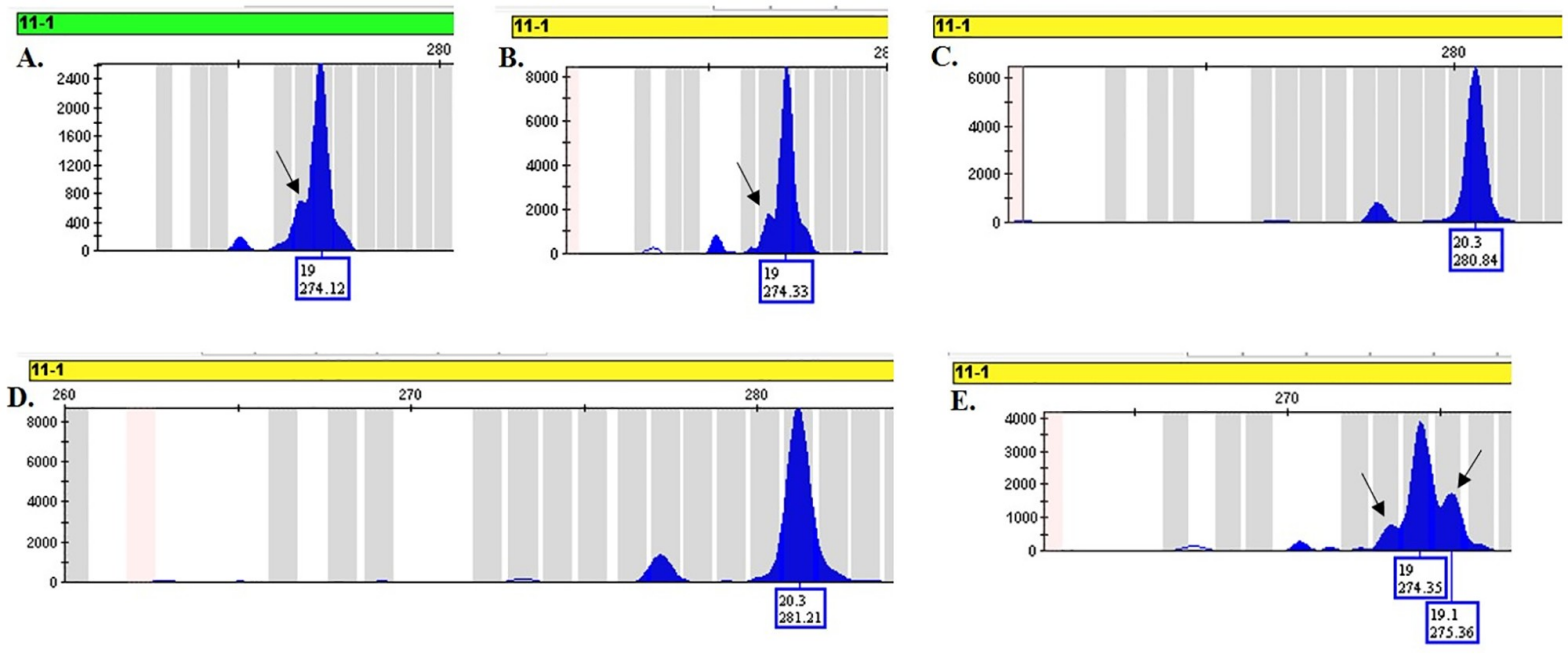
Peak morphology of STR marker 11–1. A. Arrow denotes irregular peak morphology of allele 19 from diploid DNA from an AKR mouse (Jackson Laboratories). B. Arrow denotes irregular peak morphology of allele 19 from the JC cancer cell line. C. Normal peak morphology of allele 20.3 from diploid DNA from C58 mouse (Jackson Laboratories). D. Normal peak morphology of allele 20.3 from the CMT-93 cancer cell line. E. Arrows denote irregular peak morphology of allele 19 from the CT26.CL25 cancer cell line.

STR marker 11–1 was one of the original 19 markers analyzed by the Consortium and was ultimately removed. The removal of this marker did not change the informativeness of the remaining 18 STR markers to discriminate among the 50 mouse cell lines (see S2 Table). The marker for 11–1 was removed due to an abnormal peak morphology which appears as a “shoulder” either +1 or -1 bp from the true allele (see Fig 4), leading to discordant results. This phenomenon, most likely incomplete adenylation, was seen in STR genotyping from both diploid DNA samples and cell lines, and could not be resolved with a modification of the PCR that included an increased 4 h soak time at 60 °C at the end of the thermal cycling program or with added Pigtail sequences to the 5’ end of the reverse primer [17]. The “shoulder” appears most prominently in full repeat alleles, such as allele 19, and is less of a problem for microvariants, such as allele 20.3 (Fig 4).

### Concordant results

Review of the raw data (electropherogram) for each Consortium laboratory member showed concordance for the 50 cell lines. However, using 19 STR markers data misinterpretation resulted in discordance for 32 of the 50 cell lines. The reasons for discordance include high or low DNA amount, stutter calculation errors, missed allele, bin error, and artifacts. All of these interpretation errors can be resolved with training and experience. A sample was labeled discordant if one or more alleles was miscalled, for example, the NIH/3T3 cell line resulted in 311 out of 312 correct allele calls (99.6% consensus allele calls among the Consortium laboratory members) (see Table 5). In this example, one allele (20.3) was missed at STR marker 4–2, resulting in a discordant cell line.

**Table 5:** Discordant NIH/3T3 cell line.

	STR Markers																	
	18–3	4–2	6–7	19–2	1–2	7–1	1–1	3–2	8–1	2–1	15–3	6–4	11–2	17–2	12–1	5–5	X-1	13–1
Lab	Consortium Laboratory Member Allele Calls																	
1	17,19	19.3, 20.3	12	11,12	13,17	29	10	14,15	15	9	20.3	15.3	15,17	13,14	20	14,15	25	16.2
2	17,19	19.3, 20.3	12	11,12	13,17	29	10	14,15	15	9	20.3	15.3	15,17	13,14	20	14,15	25	16.2
3	17,19	19.3, 20.3	12	11,12	13,17	29	10	14,15	15	9	20.3	15.3	15,17	13,14	20	14,15	25	16.2
4	17,19	19.3	12	11,12	13,17	29	10	14,15	15	9	20.3	15.3	15,17	13,14	20	14,15	25	16.2
5	17,19	19.3, 20.3	12	11,12	13,17	29	10	14,15	15	9	20.3	15.3	15,17	13,14	20	14,15	25	16.2
6	17,19	19.3, 20.3	12	11,12	13,17	29	10	14,15	15	9	20.3	15.3	15,17	13,14	20	14,15	25	16.2
7	17,19	19.3, 20.3	12	11,12	13,17	29	10	14,15	15	9	20.3	15.3	15,17	13,14	20	14,15	25	16.2
8	17,19	19.3, 20.3	12	11,12	13,17	29	10	14,15	15	9	20.3	15.3	15,17	13,14	20	14,15	25	16.2
9	17,19	19.3, 20.3	12	11,12	13,17	29	10	14,15	15	9	20.3	15.3	15,17	13,14	20	14,15	25	16.2
10	17,19	19.3, 20.3	12	11,12	13,17	29	10	14,15	15	9	20.3	15.3	15,17	13,14	20	14,15	25	16.2
11	17,19	19.3, 20.3	12	11,12	13,17	29	10	14,15	15	9	20.3	15.3	15,17	13,14	20	14,15	25	16.2
12	17,19	19.3, 20.3	12	11,12	13,17	29	10	14,15	15	9	20.3	15.3	15,17	13,14	20	14,15	25	16.2

With the removal of STR marker 11–1, the Consortium laboratory members had concordant STR profiles for 21 of the 50 cell lines tested (see Table 6). Allele calls for these 21 cell lines were in full concordance among the Consortium laboratory members at 18 STR loci.

**Table 6:** Concordant STR profiles for 21 mouse cell lines.

	Cell Lines																				
STR Marker	NCTC clone 929	BALB/ 3T3 clone A31	L1210	CMT-93	STO	NMuMG	P19	M-NFS-60	Hepa-1c1c7	M-1	JC	WEHI-13VAR	RAW 264.7 gamma NO-	EOC 20	EMT6	YAC-1	M1	BCL1 clone 5B1b	2E8	J558	RAW 264.7
	Alleles																				
18–3	16	18	16,17	16	19	18	16,17	17	16,17	17	18,19	17,18	18	16	18,19	18	18	18,19	18	18	18
4–2	20.3	21.3	20.3	20.3	20.3	20.3	13,21.3	19.3,20.3	18.3,19.3	20.3	21.3	21.3,22.3	22.3,23.3	19.3	21.3	21.3	20.3	21.3	21.3	21.3	22.3
6–7	12	12	12	15	12	15	12	12	15	12	12	12	12	12	12	12	16	12	12	12	12
19–2	12	14	12	14	12	12	12,14	11,12	10,11	13	14	13	12,14	12	13	12,13	13,14	12,13	14	14	14
1–2	17	17	16	19,20	16	17	16,17	17	13	19	17,18	17	17	16	17,18	17,18	13	17	17	17	17
7–1	25,26,27	25.2	27,28	26.2	25.2	25.2	26	28,29	25.2	26.2	24.2	25.2	25.2	26	25.2	25.2	25.2	26.2	24.2	25.2	25.2
1–1	10	16	15,17	15	12	15, 16	10	10,16	14,15	17	14	15	15,16	10	15	11	16	14	15	16	15,16
3–2	13,14	14	13	14	14	14	14	13,14	10	14	14	14	14	14	14	14	12	14	14	14	14
8–1	16	13	16	16	16	16	16	15,16	15	16	13	13	13	16,17	13	16	16,17	13	13	13	13
2–1	9	16	15	16	9	15, 16	9	9,16	14	9,16	16	16,17	16	9	16	16	9	15	16	16	16
15–3	24.3,25.3,26.3	22.3	21.3,22.3	22.3	20.3	21.3, 22.3	26.3	20.3,21.3	17,18,19	22.3	22.3,23.3	22.3	22.3	25.3	22.3,23.3	22.3	22.3	22.3	23.3.24.3	22.3	22.3
6–4	17,18	18	17	18,19	15.3	17,18	18	15.3,18	18,19	15	18	18,19	18	19	18	18	16.3	18	17	18	18
11–2	15,16	17	17	16	15	15	16	17,19	16	16	17	17,18	17	17	18	15	15,16	18	16,17	17	17
17–2	15	15,16	16	15,16	13	16,17	15	13,15	14	15	16	16	14,16	16	16	16	12	15	17	16	14,16
12–1	16	16	16	17	19	16	16	16,20	17,18	17	16	16	16,17	16	16	16	16,17	16	16	16	16
5–5	14	14	13	14,16,17	14	15	14	14,15	17,18	17	14	13,14	14	15	13,15	16	14	13,14	14	14	14
X-1	26,27	25	27	27	26	27,28	26	25,27	25	28	25	26	25	27	24	26	26	24,25	26	25	24
13–1	17	16.2	17	17	16.2	16.2,17	17	13,15.2	17.1	17	16.2	15.2	16.2	17	16.2	16.2,17.2	16.2	16.2	16.2	16.2	16.2

Although the remaining 29 cell lines were discordant, the percent of accurately calling alleles among the Consortium laboratory members were above 92% for all cell lines. Each discordant cell line is summarized with an average percent allele agreement, the corresponding number of STR markers that were discordant, and the reasons for discordance (Table 7).

**Table 7:** Summary of discordant cell line.

	At 18 Mouse STR Markers		
Cell Line Name	Mean % Groups Agree on Allele Calls	Number of Markers Discordant	Reason(s) for Discordance *
A9	0.989	2	b
3T3-Swiss Albino	0.981	2	a, d
P3X63Ag8.653	0.994	2	b
Sp2/0-Ag14	0.984	4	b, c, e
LL/2 (LLC1)	0.991	4	a, b, d, e
NIH/3T3	0.997	1	b
McCoy B	0.981	3	a, b, d
WEHI 164	0.991	1	c
Hepa 1–6	0.986	2	a, b, e
HT-2 clone A5E	0.953	7	a, b, c
MLTC-1	0.993	1	b
E.G7-OVA	0.963	5	b
mIMCD-3	0.985	2	d
MS1	0.993	1	b
4T1	0.993	2	b
W-20-17	0.997	1	e
CT26.WT	0.977	4	b
CT26.CL25	0.990	2	b
Renca	0.997	1	b
B16-F0	0.990	2	b
B16-F1	0.995	2	b
A20	0.992	1	b
CTLL-2	0.991	1	a
D10.G4.1	0.982	3	b, e
EL4	0.963	5	a, b, d
C1498	0.967	4	b, c
P815	0.989	2	a, b, c
Sarcoma 180	0.918	16	a, b, c
WEHI-3	0.985	5	b, c, e

*Reasons for discordance are coded as follows: a. low fluorescent signal, b. error in calculation of stutter ratio, c. stutter ratio was at the stutter threshold, d. missed allele (not labeled), e. artifact (pull-up, dye blob, spike)

### Cell lines that require further characterization

Eight cell lines, HT-2 clone A5E, Sarcoma 180, McCoy B, BALB/3T3 clone A31, J558, E.G7-OVA, EL4, and C1498 were removed for misidentification, cross-contamination, or lack of consensus of allele calls among Consortium laboratory members. These eight cell lines will not be included in the NCBI BioSample database entry. A spleen cell line, HT-2 clone A5E, reported in the literature as being derived from a BALB/c mouse strain [18] exhibited greater similarity with a C57BL strain by sharing over 50% alleles. This cell line also has three alleles or more at seven loci, high peak height imbalance, and is discordant at eight STR markers. The HT-2 clone A5E cell line needs more characterization to determine if it is contaminated or misidentified due to the inconsistencies with the strain of origin and the increase of alleles per marker. Sarcoma 180 is another cell line that has high peak height imbalance and greater than three alleles at 17 loci, making it difficult to apply stutter filter guidelines. Additionally, two cell lines J558 and BALB/3T3 clone A31, purported to be unrelated, have 95% allele matching based on pairwise comparison using Master’s algorithm (see S3 Table). These three cell lines requires further characterization to determine authenticity. There is limited information in the literature regarding a clear origin for the McCoy B cell line, which was initially described as originating from the parental cell line McCoy A, derived from synovial fluid in the knee from a human patient [19]. McCoy B cell line is of mouse origin, not human, and is a clear indication of misidentification [20]. The additional cell lines, E.G7-OVA, EL4, and C1498 have less than 97% consensus agreement in allele calls (see Table 7), to include discordant calls at greater than four loci, and greater than three alleles present at a specific locus. These disagreements call for further characterization of these cell lines.

### Pairwise comparison

A pairwise comparison using Master’s algorithm (percent match = number shared alleles/total number of alleles in the questioned profile) [21] was calculated on the mouse cell lines and control diploid mouse strains from commercial sources (Jackson Laboratories and Zyagen, Inc.) (see S3 Table). When a cell line was compared against itself it showed 100% matching. However, cell lines compared to its derivatives demonstrated a percent matching greater than 85% (see Table 8). Moreover, cell lines derived from the same mouse strain, in some cases, showed high levels of relatedness (over 80%). Based on the matching algorithm data, there are several cell lines that need further studies due to high level of relatedness.

**Table 8:** Percent matching of known related cell lines using Master’s algorithm.

Parental Cell Line	Derivative of parental cell line	Percent Matching
NCTC clone 929	A9	85%
WEHI 164	WEHI-13VAR	91%
CT26.WT	CT26.CL25	87%
B16-F0	B16-F1	94%
RAW 264.7	RAW 264.7 gamma NO-	95%

## Discussion

The mouse multiplex PCR assay for intraspecies mouse cell line identity testing was validated by the Mouse Cell Line Authentication Consortium. Overall, each Consortium laboratory member, using various instrumentation, polymers and arrays, generated STR profiles for the 50 mouse cell lines. Review of the electropherograms from the Consortium laboratory members showed concordance for the 50 cell lines. Cell lines that were known to be related showed greater than 85% matching alleles using Master’s algorithm. Several reported related and unrelated cell lines showed greater than 80% allele matching and require further investigation. Data misinterpretation resulted in discordance based on 18 of the 19 STR markers. All of these discordant allele calls can be resolved with training and experience.

The Consortium started out with 19 STR markers to validate mouse cell lines and test for human contamination. It is recommended by the Consortium that marker 11–1 be removed from the multiplex PCR assay due to the abnormal peak morphology in the amplified products. The removal of this marker will not affect the overall informativeness of the remaining 18 mouse STR markers and their ability to discriminate between the fifty mouse cell lines tested.

Future plans include collaboration between NIST and ATCC to develop an allelic ladder to increase allele coverage and aid in more accurate allele calls. A written consensus standard for mouse cell line authentication would be useful to the community to establish rules and guidelines. Points to be considered for a future consensus standard include: allelic ladder, stutter filter recommendations, DNA quantitation methods, and troubleshooting. The NIST-validated STR profiles from this study have be added to the NCBI BioSample database (https://www.ncbi.nlm.nih.gov/biosample/?term=mouse+cell+line+STR+profile%5Battribute+name%5D) with BioProject accession number PRJNA539973. The cell lines listed in the database, having greater than 98% allele consensus among the Consortium laboratory members, have background information included, allele calls for 18 mouse STR markers, and their associated electropherograms.

Finally, it is important to note that concordance in STR profile among the Consortium determines the accuracy to perform and interpret the STR results and does not demonstrate complete authenticity of a particular cell line. However, there is enough confidence that a concordance value greater than 98% provides an accurate STR profile for the identity of mouse cell lines. The STR profiles published on the NCBI BioSample database are validated by the Consortium based on the cell lines provided by ATCC. These STR profiles are the first step in authentication of mouse cell lines.

## Materials and methods

Several activities were performed by NIST in preparation of the interlaboratory study. Some of these activities included:

1. Preparation of DNA from 50 mouse cell lines and one human cell line
2. Calculating stutter ratios from diploid mouse DNA samples
3. Preparation of calibrants
4. Preparation of primers
5. Preparation of kit
  - Reagents
  - Protocols

### Preparation of cell line DNA samples

Fifty-one cell lines (50 mouse, one human) of seed stock material were provided by ATCC (see Table 9) to NIST for the interlaboratory study for mouse STR genotyping. Vials of frozen cell lines were stored in a -150 °C freezer upon receipt.

**Table 9:** Cell lines used in the interlaboratory study.

Code	ATCC#	Cell Line Designation	Mouse Strain	Tissue Type and/or Disease
M01	CCL-1	NCTC clone 929	C3H/An	Subcutaneous connective tissue
M02	CCL-1.4	A9	C3H/An	Subcutaneous connective tissue
M03	CCL-163	BALB/3T3 clone A31	BALB/c	Embryo
M04	CCL-219	L1210	DBA/2	Lymphocytic leukemia
M05	CCL-223	CMT-93	C57BL/icrf	Rectal, Polyploid carcinoma
M06	CCL-92	3T3-Swiss albino	Swiss	Embryo
M07	CRL-1503	STO	SIM	Mouse embryonic fibroblasts
M08	CRL-1580	P3X63Ag8.653	BALB/c	Plasmacytoma/Myeloma
M09	CRL-1581	Sp2/0-Ag14	BALB/c	Spleen, Myeloma
M10	CRL-1636	NMuMG	NAMRU	Mammary gland
M11	CRL-1642	LL/2 (LLC1)	C57BL	Lewis Lung carcinoma
M12	CRL-1658	NIH/3T3	Swiss	Embryo
M13	CRL-1696	McCoy (McCoy B)	C3H/An	Synovial fluid in the knee joint
M14	CRL-1751	WEHI 164	BALB/c	Fibrosarcoma
M15	CRL-1825	P19	C3H/He	Teratocarcinoma/Embryonal carcinoma
M16	CRL-1830	Hepa 1–6	C57L	Liver, Hepatoma
M17	CRL-1838	M-NFS-60	NFS/N (NFSxDBA/2)	Blood, Myelogenous leukemia
M18	CRL-1841	HT-2 clone A5E	BALB/c	Spleen
M19	CRL-2026	Hepa-1c1c7	C57L	Liver, Hepatoma
M20	CRL-2038	M-1	Tg(SV40)Bri/7	Normal renal tissue, SV-40 transfected
M21	CRL-2065	MLTC-1	C57BL/6	Testis, Leydig cell tumor
M22	CRL-2113	E.G7-OVA	C57BL/6N	Blood, Lymphoma
M23	CRL-2116	JC	BALB/cRos	Mammary adenocarcinoma
M24	CRL-2123	mIMCD-3	Tg(SV40)Bri/7	Kidney medullary duct, SV-40 transfected
M25	CRL-2148	WEHI-13VAR	BALB/c	Fibrosarcoma
M26	CRL-2278	RAW 264.7 gamma NO(-)	BALB/c	Macrophage, virus transformed
M27	CRL-2279	MS1 (Mile Sven 1)	C57BL/6	Pancreas/islet of Langerhans, SV-40 transfected
M28	CRL-2469	EOC 20	C3H/HeJ	Brain
M29	CRL-2539	4T1	BALB/cfC3H	Breast, Mammary tumor (stage IV)
M30	CRL-2623	W-20-17	W++	Bone marrow, Stroma
M31	CRL-2638	CT26.WT	BALB/c	Colon carcinoma
M32	CRL-2639	CT26.CL25	BALB/c	Colon carcinoma
M33	CRL-2755	EMT6	BALB/cCrgl	Breast, Mammary carcinoma
M34	CRL-2947	Renca	BALB/cCr	Kidney, Renal adenocarcinoma
M35	CRL-6322	B16-F0	C57BL/6J	Skin, Melanoma
M36	CRL-6323	B16-F1	C57BL/6J	Skin, Melanoma
M37	TIB-160	YAC-1	A/Sn	Lymphoma, MLV*-induced
M38	TIB-192	M1	SL	Lymphocyte, Myeloid leukemia
M39	TIB-197	BCL1 clone 5B1b	BALB/c	Leukemia/Lymphoma
M40	TIB-208	A20	BALB/cAnN	B lymphocyte, Reticulum cell sarcoma
M41	TIB-214	CTLL-2	C57BL/6	Cytotoxic T lymphocyte
M42	TIB-224	D10.G4.1	AKR/J	Lymph Node, Helper T lymphocyte
M43	TIB-239	2E8	BALB/c.xid	Bone marrow
M44	TIB-39	EL4	C57BL/6N	T lymphocyte, Lymphoma
M45	TIB-49	C1498	C57BL/6J	Lymphocyte, Acute myeloid leukemia
M46	TIB-6	J558	BALB/c	B lymphocyte, Plasmacytoma/myeloma
M47	TIB-64	P815	DBA/2	Mast cell, Mastocytoma
M48	TIB-66	Sarcoma 180	Swiss Webster	Ascites, Sarcoma
M49	TIB-68	WEHI-3	BALB/c	Peripheral blood, Leukemia
M50	TIB-71	RAW 264.7	BALB/c	Ascites, AMLV*-induced tumor
H1	CRL-1486	HEPM (Human)	Not applicable	Palatal mesenchyme

*Moloney leukemia virus (MLV); Abelson murine leukemia virus (AMLV)

#### DNA isolation

DNA extraction was performed on the washed cell pellets using Quick-DNA Miniprep (Zymo Research, Irvine, CA, USA) following the manufacturer’s protocol for Cell Monolayer Samples.

#### DNA concentration

DNA concentrations were determined using the Quant-iT Qubit dsDNA BR Assay Kit (Invitrogen, Carlsbad, CA, USA) and fluorescence was measured using the Qubit 3 Fluorometer (Invitrogen). Based on those values, the DNA was diluted to 2 ng/μL in TE (10 mM Tris-HCl (pH 8.0), 0.1 mM EDTA) (Ambion, Foster City, CA, USA) and final DNA concentrations were determined using the Quant-iT Qubit dsDNA HS Assay Kit (Invitrogen) and fluorescence measured using the Qubit 3 Fluorometer (Invitrogen).

#### DNA purity

DNA purity was determined by absorbance using the Synergy MX and the Take3 micro-volume plate (BioTek, Winooski, VT, USA) with an optical pathlength of 0.5 mm. A260/A280 ratios were between 1.83 to 1.92 (values greater than or equal to 1.8 are ideal for DNA purity).

Fifty-one cell line DNA samples were blinded and provided a code (mouse cell lines were coded M01 through M50 and the human cell line was coded H1). 20 μL of each DNA sample, concentrations ranging from 2 ng/μL to 2.7 ng/μL, were aliquoted into labeled 0.2 mL 8-strip tubes. Each of the Consortium laboratory members received duplicate DNA samples.

### Diploid mouse DNA samples used to calculate stutter ratios

Genomic DNA samples from 48 mouse strains obtained from Jackson Laboratories (Bar Harbor, ME, USA), two mouse strains obtained from Zyagen (San Diego, CA), and 15 wild-caught mice (courtesy of Dr. Michael Nachman, University of Arizona, USA) were used in calculating stutter ratios for normal diploid mouse samples to create stutter filter recommendations.

### Preparation of calibrants from diploid mouse DNA

Genomic DNA samples from 12 mouse strains obtained from Jackson Laboratories were used to create five calibrant DNA samples (C1 through C5). The DNA calibrant samples were prepared from the following 12 strains of mice: C58/J, CE/J, BTBR T<+> tf/J, NZW/LacJ, NOD/ShiLtJ, WSB/EiJ, A/J, KK/HlJ, MA/MyJ, C3H/HeJ, I/LnJ, and NZB/BlNJ. The calibrants were used to assign alleles. Sanger sequencing was performed on each strain at all 19 STR loci to correlate the number of repeating units with fragment length. These highly characterized DNA samples were diluted to approximately 2 ng/μL in TE buffer (10 mM Tris-HCl, pH 8.0, 0.1 mM EDTA) (Ambion) and quantified using Qubit dsDNA HS Assay Kit (Invitrogen) and Qubit 4 Fluorometer (Invitrogen). The various DNA samples were combined in a single tube to prepare each calibrant

- Calibrant 1 (C1): C58/J, CE/J, BTBR T<+> tf/J were combined at a 1:1:1 ratio, each 2 ng/μL
- Calibrant 2 (C2): NZW/LacJ, NOD/ShiLtJ, WSB/EiJ were combined at a 1:1:1 ratio, each 2 ng/μL
- Calibrant 3 (C3): A/J, KK/HlJ, MA/MyJ were combined at a 1:1:1 ratio, each 2 ng/μL
- Calibrant 4 (C4): C3H/HeJ and I/LnJ were combined at a 1:1 ratio, each 2 ng/μL
- Calibrant 5 (C5): NZB/BlNJ, 2 ng/μL

Calibrants were aliquoted into 0.2 mL 8-strip tubes (20 μL) in triplicate for all Consortium laboratory members. The STR profile for Calibrant 1 is shown in Table 10 and an example of the associated electropherogram for Calibrant 1 can be found in S2 Fig.

**Table 10:** STR profile for Calibrant 1 (C1).

STR Marker	Alleles
18–3	16,19,21
4–2	16.3,19.3,20.3
11–1	19.3,20.3
6–7	12,15,16
19–2	12,13,14
1–2	13,15,18
7–1	26.2,28,29
1–1	11,14
3–2	13.1,14
8–1	14,15,16
2–1	9
15–3	20.3,22.3,23.3
6–4	15.3,18,18.3
11–2	15,15.3,18
17–2	15,16
12–1	15,16,18
5–5	13,16,17
X-1	24,25,28
13–1	16.2,17,18

### Preparation of primers to target mouse STR markers

Primers for 19 mouse STR markers were developed for intraspecies identification of mouse cell lines. The multiplex PCR primers target the following 19 mouse loci: 18–3, 4–2, 11–1, 6–7, 19–2, 1–2, 7–1, 1–1, 3–2, 8–1, 2–1, 15–3, 6–4, 11–2, 17–2, 12–1, 5–5, X-1 and 13–1. The repeat motif for each STR marker was determined by sequencing to correlate fragment length with repeat number. In addition to the mouse STR markers, two well characterized human STR markers D8S1106 and D4S2408, previously described to identify human and African green monkey DNA [22, 23], are also included in the kit to screen for contamination of mouse cell lines with human cells (Table 11). Table 11 contains the primer sequences that make up the multiplex PCR assay. Forward primers were labeled with one of the following fluorescent dyes at the 5’ end: 6FAM (blue), VIC (green), NED (yellow), or PET (red) (Applied Biosystems). In some cases, an additional guanine base (G) or a ‘‘PIGtail” sequence (GTTTCTT) was added to the 5’ end of the unlabeled reverse primer to promote complete adenylation and are underlined in the reverse primer [17]. The final primer concentrations for the forward and reverse primers in a 20 μL reaction volume were optimized based on DNA concentration and the number of cycles in the PCR program.

**Table 11:** Mouse multiplex PCR assay.

Marker	Species	Chr Loc.	Forward Primer (5'-3')	Reverse Primer (5'-3')	Final [Primer] in 20μL (μM)
18–3	mouse	18	[6FAM]-TCTTTCTCCTTTTGTGTCATGC	GTTTCTTGCTAAATAACTAAGCAAGTGAACAGA	150
4–2	mouse	4	[6FAM]-AAGCTTCTCTGGCCATTTGA	GTTCATAAACTTCAAGCAATGACA	200
11–1	mouse	11	[6FAM]-CCAGCCAGCCACTCTAGTTA	GTTTCTTACAGCTTCAGGACCATGGAA	150
6–7	mouse	6	[6FAM]-AGTCCACCCAGTGCATTCTC	GTTTCTTCATGTGGCTGGTATGCTGTT	200
19–2	mouse	19	[6FAM]-AGGCTAGCACTGTTCCTTGT	GTTTCTTACTCAGCACCTTCCATCCTG	200
1–2	mouse	1	[VIC]-TCTTTAAAAATCAAACAGGCAAA	GTTTCTTGGGGAGGTTGGGGTGTATAA	225
7–1	mouse	7	[VIC]-ACACCAGTTGGGGACTAGGA	GTTTCTTAACTGGTGCTGGGTCTGAAC	200
1–1	mouse	1	[VIC]-CCCTTCACTCCTTCATTCCA	GTTTCTTTGAGCCTAAGGACCTGGACA	150
3–2	mouse	3	[VIC]-TGAGCTACCATGTGGGTACA	GTTTCTTCACACACACACACAAAGATGGA	200
8–1	mouse	8	[VIC]-AGTAATATCCTGGTCCTGGCC	GTTTCTTGAGCTCACTATGTAGCTATTGGA	200
2–1	mouse	2	[NED]-ACAACTTCAAACTTTGCTGGAA	GTTTCTTTCCTGCTTAGCTTGTCATTTCC	125
15–3	mouse	15	[NED]-TCTGGGCGTGTCTGTCATAA	GTTTCTTAGGCTATCTAAACTTGACATGCT	100
6–4	mouse	6	[NED]-TTTGCAACAGCTCAGTTTCC	GTTTCTTAATCGCTGGCAGATCTTAGG	200
11–2	mouse	11	[NED]-AAGGCAGGGGAATTCACAGT	GTTTCTTTCTCACCATTGCAGTCCTGA	200
17–2	mouse	17	[PET]-CTCTTCTCCATCCGTCCATC	GTTTCTTATGGCTCTTGCCACAAATCT	200
12–1	mouse	12	[PET]-TTTCAAAATTGTCATTGAACACA	GTTTCTTTGGTCCTTCAGTATCATCCTTG	200
5–5	mouse	5	[PET]-CGTTTTACCTGGCTGACACA	GTTTCTTTGGTTTAAAACTCAATACCAAACAA	250
X-1	mouse	X	[PET]-AACAAAAATGTCCCTCAATGC	GTTTCTTAAGGTATATATCAAGATGGCATTATCA	275
13–1	mouse	13	[PET]-AACTCCCACTGCTTCTTGGT	GTTTCTTGCCAAAGTAAACCCTTCTCCC	250
D8S1106	human	8	[VIC]-TCTGCTGATTGTGCCCATTG	GTTTCTTATTGGCTGGTTAGTGACACA	200
D4S2408	human	4	[NED]-TCATTTCCATAGGGTAAGTGAAAA	GTTTCTTGCCATGGGGATAAAATCAGA	250

### Characterization of calibrants

#### PCR amplification for sequencing

Sequencing reactions for calibrant samples (C1 through C5) were prepared using the following 20 μL reaction mixture: 4–5 ng of template DNA, 1X GeneAmp PCR Gold buffer (Thermo Fisher), 1.5 mM MgCl_2_ (Thermo Fisher), 1 U AmpliTaq Gold Polymerase (Thermo Fisher), 0.15 μM unlabeled forward and reverse primers (see S6 Table), 0.16 mg/mL non-acetylated BSA (Invitrogen), 200 μM dNTPs (Invitrogen), and PCR grade water. The SeqAmp program was run on the Veriti (Applied Biosystems) with the following parameters: denaturation for 10 min at 95 °C, amplification for 35 cycles of 1 min at 94 °C, 1 min at 55–60 °C (annealing temperature specific to individual primers), and 1 min at 72 °C, extension for 45 min at 60 °C, followed by a hold at 25 °C.

#### Sequencing STR markers in calibrants

To determine the number of repeat units for each fragment length (allele) for the 19 mouse STR markers, each calibrant was subjected to Sanger sequencing. Each calibrant (C1 through C5) DNA sample was amplified with primers targeting all 19 mouse STR markers in single-plex. Amplicons were treated with 2 μL of ExoSap-IT (Applied Biosystems) per 5 μL of PCR product to remove unincorporated primers and deoxyribonucleotide triphosphates (dNTPs) by incubating samples for 90 min at 37 °C followed by 20 min at 80 °C to inactivate the enzymes. Samples were then sent to Eurofins MWG Operon (Louisville, KY, USA) for Sanger sequencing. Resulting sequence profiles were received after data analysis was performed by Eurofins MWG Operon. Lasergene 15.2 (DNA Star, Madison, Wisconsin, USA) alignment software, SeqMan Pro, was used to determine repeats for each allele at every STR marker.

#### PCR amplification for genotyping calibrants

PCR amplification of calibrant samples (C1 through C5) and optimization of CE parameters was completed by the Consortium laboratory members prior to running the full panel of mouse cell line DNA (M01 through M50). Detailed methods are described in S1 File but will be explained briefly here. Each PCR reaction contained 12.5 μL of Qiagen Type-It Microsatellite PCR Mix (MM), 2.5 μL of primers (P21), 9 μL of PCR grade water, and 1 μL of DNA template (approximately 2ng/μL). To improve signal in samples, many labs had to increase the input DNA volume to 2 μL in the reaction. PCR reactions were placed on a thermal cycler with the following parameters: denaturation at 95 °C for 5 min, 29 cycles of amplification step (95 °C for 30 sec, 59 °C for 90 sec, 72 °C for 30 sec), soak at 60 °C for 90 min, and a hold at 25 °C. After optimization of the assay was completed using calibrant DNA, PCR was performed on the full panel of mouse cell line DNA. PCR Master Mix for the 50 mouse DNA samples (M01 through M50), five Calibrants (C1 through C5), one human DNA control (H1), and one negative control (water) was prepared using the same conditions as described for the calibrants.

#### Separation and analysis of PCR products

PCR amplicons were prepared for capillary electrophoresis by adding 9.5 μL of Hi-Di Formamide (Applied Biosystems) and 0.5 μL of GeneScan 600 v2 LIZ internal size standard (Applied Biosystems) to 1 μL of PCR product to each well in a 96-well plate. The 96-well plate was briefly centrifuged at 2000 x g for one minute. The plate was loaded onto the Applied Biosystems 3500xl Genetic Analyzer (or equivalent) (use a STR cassette base if needed). A five-dye DS-33 Matrix Standard Kit (Applied Biosystems) was previously established under the G5 filter with the following dyes: 6FAM, VIC, NED, PET, and LIZ (as per manufacturer’s instruction). Conditions for running an Applied Biosystems 3500xl 36cm array using POP-4 polymer was provided to the Consortium laboratory members and specific adjustments were made individually based on the instrument, array, and polymer being used. The conditions for capillary electrophoresis (CE) using a 3500xl, 36cm array, POP-4 were as follows: oven temp (60 °C), run time (1500 sec), run voltage (15kV), pre-run time (180 sec), pre-run voltage (15 kV), injection time (15 sec), injection voltage (1.2 kV), and data delay (1 sec). Consortium laboratory members were provided a specific set of files for their version of software (which may include bins and panels, analysis method, plot settings, size settings, and table settings).

### Preparation of mouse STR validation kit and protocol distribution

The kits were prepared at NIST. Each kit provided to a Consortium laboratory member contained the following:

- DNA from 50 mouse cell lines (two tubes of each mouse cell line DNA; each tube containing 20 μL of 2 ng/μL of DNA). Each mouse cell line DNA was numerically coded for blind experimentation to avoid experimental bias (M01 through M50)
- Five Calibrant DNA samples labeled C1 through C5 (three tubes of each calibrant; each tube containing 20 μL of 2 ng/μL DNA)
- One human DNA control sample labeled H1 (two tubes of human DNA each containing 20μL of 2 ng/μL DNA)
- PCR Master Mix (Qiagen Type-It Microsatellite PCR Kit, Qiagen, Hilden, Germany) labeled MM. (Three tubes of Master Mix; each containing 0.850 mL)
- Primers for 19 mouse STR markers and two human STR markers labeled P21 (each tube containing 400 μL). Primer mix was tested for signal intensity and presence of accurate allele calls using the calibrant DNA samples and the multiplex PCR assay prior to distribution.

Kits were distributed on dry ice to each Consortium laboratory member by ATCC under appropriate DOT shipping regulations.

### Instructions to consortium laboratory members

The following instructions were given to the Consortium laboratory members. Upon receipt, kit should be stored at -20 °C. Once kit components were thawed for use, subsequent storage of kit components were as follows: Qiagen Type-It Microsatellite PCR Master Mix (MM) should be stored at -20 °C and all DNA samples (M01-M50), Calibrants (C1-C5), and Human Control (H1), and should be stored at 4 °C. Mouse Multiplex Primers (P21) are light sensitive and should be stored in the dark at 4 °C.

In addition to receiving the test kit provided by NIST/ATCC, each Consortium laboratory member was responsible for purchasing the internal size standard GeneScan LIZ 600 v2 (Applied Biosystems) and DS-33 G5 dye matrix (Applied Biosystems) to allow for proper sizing of PCR fragments and to calibrate the CE instrument for the fluorescent dyes used in the assay. The following files were distributed to each consortium member describing PCR sample preparation, thermal cycling conditions, CE conditions, software analysis and settings, and interpretation of data (see supplemental materials):

- Protocol 1: STR profiling of mouse cell lines (S1 File)
- Protocol 2: Data Analysis: GeneMapper ID-X Software; GeneMapper IDv3.2.1, GeneMapper v4.0, v4.1, v5.0 Software (S2 File)
- Protocol 3: Data Analysis: SoftGenetics GeneMarker Software Version 1.85; Version 2.6.3 or Version 2.7.6 (S3 File)
- STR Profiles for Five Calibrant DNA Samples (S4 Table)
- Extrapolation of alleles for bin set (S5 Table)
- Electropherograms for each of the five calibrants (S2–S6 Figs)
- Bins and panels, analysis methods, table settings, plot settings, size settings (S4 File)

Using the protocols, tables and bins/panels provided, each lab was asked to adjust the bins and panels in their respective software platform using the calibrant samples prior to running the 50 mouse DNA samples. Once the instruments and protocols were optimized for the calibrant samples, all samples were genotyped and analyzed. A successful run was determined by the presence of all alleles in the calibrant samples detected above threshold levels, amplification of the human control (H1) at the human STR markers, and no amplification of alleles in the negative control.

### Stutter filter calculations

Stutter ratios were calculated by dividing the height of the stutter peak by the height of the allelic peak [24]. Only peak heights above 100 RFUs were used in stutter ratio calculations. The general stutter guidelines were borrowed from human cell line authentication [7] and are as follows: 1) A peak in the -4 stutter position with a stutter ratio greater than 20% would be called an allele, and 2) A peak in the +4 stutter position with a stutter ratio above 10% would be called an allele.

To assess whether stutter ratios were affected by measurement platform, a Friedman test [15] was conducted using average stutter ratio (normalized to account for variation due to allele length) from each marker for each instrument as input. For each marker, the four considered instruments are ranked in order of average stutter ratio. A Friedman test looks for patterns in these rankings to assess whether any instrument tends to produce higher or lower stutter ratios than the other instruments. A Friedman test does not make any assumptions about the specific distributional form of the stutter ratios, and accounts for differences in average stutter ratios across markers.

Stutter ratios were also calculated for each allele present in the collection of genomic DNA from Jackson Laboratories, Zyagen, and wild-caught mouse samples from Michael Nachman’s lab at the University of Arizona. Allele-specific and marker-specific stutter ratios were determined for all alleles present in the samples tested. Allele-specific stutter filters were computed as the average of stutter ratios reported for the allele across samples plus three standard deviations. Marker-specific stutter filters were computed as the average of all reported stutter ratios across alleles within a marker plus three standard deviations [16]. Incorporating stutter filters in fragment analysis software will aid in future allele calling and flag samples with peak height imbalances that may need further review.

### Concordance

A cell line was considered concordant if all Consortium laboratory members obtained the same allele calls at all 19 loci. For each allele call within each cell line, an agreement proportion was computed as the fraction of the other 11 Consortium laboratory members that also called that allele. For example, suppose for a given marker in a given cell line that 12 Consortium laboratory members called allele 17 and 11 Consortium laboratory members called allele 19 and no other allele calls were made for this marker. The allele agreement for each of the allele 17 calls would be 11/11 = 1, and the allele agreement for each of the allele 19 calls would be 10/11 = 0.909. Allele agreement for each marker was determined by calculating the average agreement proportion across all allele calls for that marker. The agreement score for the marker in the example above would be 12*(1111)+11*(1011)23=0.957, where 23 appears in the denominator because that is the total number of allele calls made for this marker across all groups. Allele agreement was also assessed at the cell line level by computing the average agreement proportion across all alleles and markers. As the term, “agreement proportion” implies, this measure of correspondence conveys the average proportion of the other labs that would agree with a given allele call.

## Supporting information

S1 File Protocol 1: STR profiling of mouse cell lines. Detailed instructions were provided to the Consortium laboratory members regarding sample handling, PCR conditions, and preparing samples for capillary electrophoresis.
(PDF)

S2 File Protocol 2: Data analysis using GeneMapper software. Data Analysis instructions for GeneMapper ID-X Software; GeneMapper IDv3.2.1, GeneMapper v4.0, v4.1, v5.0 Software platforms.
(PDF)

S3 File Protocol 3: Data analysis using GeneMarker software. Data Analysis instructions for SoftGenetics GeneMarker Software Version 1.85; Version 2.6.3 or Version 2.7.6 platforms.
(PDF)

S4 File Bins, panels, analysis method, plot setting, size setting, and table setting for each software platform used in the interlaboratory study (compressed file). (ZIP)

S1 Table Allele-specific stutter ratios. Average allele-specific stutter ratios were determined using normal diploid mouse strain DNA samples. The upper stutter limit was calculated as the mean plus 3 standard deviations, where the standard deviation corresponds to the collection of stutter ratios observed for that specific allele in that specific marker.
(XLSX)

S2 Table Genotype uniqueness of DNA samples using 18 out of 19 STR markers. This table reports each sample’s nearest neighbor (where distance between samples is assessed as the total number of alleles across markers that are present in one sample but not the other).
(XLSX)

S3 Table Pairwise comparison using Master’s algorithm. A pairwise comparison of the mouse cell lines and diploid mouse strains was performed using Master’s algorithm.
(XLSX)

S4 Table STR profiles for the five calibrant DNA samples. Alleles present for each calibrant at 19 mouse STR markers.
(XLSX)

S5 Table Extrapolation of alleles for bin set. The table shows possible alleles present for each mouse STR marker. Alleles highlighted in yellow are represented in the calibrant samples provided. Extrapolate fragment lengths of calibrants +4 bp or -4 bp to unrepresented alleles to aid in allele determination of test samples.
(XLSX)

S6 Table Sequencing primers used to characterize the calibrant DNA samples. Primers were used to sequence STR regions to determine actual number of repeats (alleles) and correlate with fragment length obtained using capillary electrophoresis.
(XLSX)

S7 Table STR profiles for validated mouse cell lines at 18 STR markers. Consensus allele calls (greater than 98%) determined by the Consortium laboratory members.
(XLSX)

S1 Fig Electropherogram of human cell line control (HEPM). Amplification of human alleles present at D8S1106 and D4S2408 in the HEPM cell line using the multiplex PCR assay targeting 19 mouse STR markers and two human STR markers.
(TIF)

S2 Fig Electropherogram of Calibrant 1. STR profile shown for Calibrant 1, a mixed diploid DNA sample from C58/J, CE/J, and BTBR T<+> tf/J mice, at 19 mouse STR markers.
(TIF)

S3 Fig Electropherogram of Calibrant 2. STR profile shown for Calibrant 2, a mixed diploid DNA sample from NZW/LacJ, NOD/ShiLtJ, and WSB/EiJ mice, at 19 mouse STR markers.
(TIF)

S4 Fig Electropherogram of Calibrant 3. STR profile shown for Calibrant 3, a mixed diploid DNA sample from A/J, KK/HlJ, and MA/MyJ mice, at 19 mouse STR markers.
(TIF)

S5 Fig Electropherogram of Calibrant 4. STR profile shown for Calibrant 4, a mixed diploid DNA sample from C3H/HeJ and I/LnJ mice, at 19 mouse STR markers.
(TIF)

S6 Fig Electropherogram of Calibrant 5. STR profile shown for Calibrant 5, a diploid DNA sample from an NZB/BlNJ mouse, at 19 mouse STR markers.
(TIF)

S7 Fig Electropherogram for the A20 cell line showing balanced peak height. A20, a sarcoma cell line, is an example of a cell line that has balanced peak height at STR markers 18–3, 6–7, 1–2, and 1–1.
(TIF)

S8 Fig Electropherogram for CT26.CL25 cell line showing peak height imbalance. A colon carcinoma cell line, CT26.CL25, has several instances of peak height imbalance shown at STR markers 1–2, 1–1, 2–1, and 6–4.
(TIF)
