# Supplementary material for: Interlaboratory study to validate a STR profiling method for intraspecies identification of mouse cell lines
Source: PLoS One. 2019 Jun 20;14(6):e0218412. doi: 10.1371/journal.pone.0218412 (PMC6586308; doi:10.1371/journal.pone.0218412)
Supplement: S1 File — Detailed instructions were provided to the Consortium laboratory members regarding sample handling, PCR conditions, and preparing samples for capillary electrophoresis. (PDF) [file pone.0218412.s001.pdf]

PROTOCOL 1: STR Profiling of Mouse Cell Lines

1. INTRODUCTION/BACKGROUND

1.1. PURPOSE/SCOPE

The purpose of this protocol is to establish a reliable and validated polymerase chain reaction (PCR)-based procedure for short tandem repeat (STR) mouse cell identification at both at the individual cell line level.

The scope of this protocol is to provide a detailed instruction for mouse cell line identity testing using mouse STR markers and to list procedures for the above purpose that are to be applied directly by all Consortium Members.

1.1.1. **A kit** will be available to the Consortium Members to enable accurate and efficient analysis and characterization of mouse STR genotypes. The kit will contain the following:

1.1.1.1. High quality DNA from fifty (50) mouse cell lines (2 vials of 20 µL at 2 ng/µL) numerically coded for blind experiment.

1.1.1.2. Five Calibrant DNA samples (3 vials of 20 µL at2 ng/µL).

1.1.1.3. Human DNA control sample (2 vials of20 µL at 2 ng/µL).

1.1.1.4. Qiagen Type-It Microsatellite PCR Master Mix (3 vials; 0.850 mL/each).

1.1.1.5. Primers targeting 19 mouse and two human (control) STR markers, tested and standardized for use in mouse cell line authentication (Mouse Multiplex) (1 vial; 400 µL). These markers are outlined in Table 1 below:

Table 1. 19 Mouse STR Markers + 2 Human STR Makers			
**No.**	**Mouse STR Markers**	**Chromosome Location***	**No. of alleles**
1	18-3	18	14
2	4-2	4	16
3	11-1	11	20
4	6-7	6	20
5	19-2	19	8
6	1-2	1	14
7	7-1	7	17
8	1-1	1	10
9	3-2	3	14
10	8-1	8	13
11	2-1	2	10
12	15-3	15	23
13	6-4	6	13
14	11-2	11	21
15	17-2	17	14
16	12-1	12	16
17	5-5	5	12
18	X-1	X	18
19	13-1	13	19
20	D8S1106 (human)	8	
21	D4S2408 (human)	4	

*** Note: Diploid mouse cell lines (2N) = 40 chromosomes**

1.1.2. Each member of the Consortium is asked to follow **the protocol instructions,** as written, to use the kit and return the data within 45 days after kit has been received.

1.1.2.1. Calibrant samples must be tested within every run and cannot be extrapolated from one run to another.

1.1.2.2. Protocol should be followed as written; any deviations should be documented. Any problems with the protocol or samples should be brought to attention immediately

1.1.2.3. All DNA samples should be handled and analyzed as initially coded.

1.1.2.4. Members should return the following data set to NIST,

- Raw data, including ABI files
- Copies of the electropherograms
- A completed export data file

2. DEFINITIONS

2.1. **Amplicon:** Copies of the DNA amplified through PCR.

2.2. **Deoxyribonucleic Acid (DNA**): Nucleic acid molecule in the form of a twisted double strand (double helix) that is the major component of chromosomes and carries genetic information. DNA, which is found in all living organisms but not in some viruses, is self-replicating and is responsible for passing along hereditary characteristics from one generation to the next.

2.3. **Genetic Analyzer (e.g. 3500xL): A** capillary electrophoresis instrument which supports a wide variety of applications such as DNA sequencing and fragment sizing. It is an optical instrument that analyzes light signals originating from oligonucleotides labeled with fluorophores.

2.4. **Electropherogram**: A graph showing the relative intensity of fluorescence with respect to time: a plot of results from an analysis done by electrophoresis. An electropherogram shows a sequence of data that is produced by an automated DNA sequencing machine. Electropherograms may be used for deriving results from genealogical testing, paternity testing, DNA sequencing, and, in the context of this work order, genetic profiling.

2.5. **Fluorophore:** A molecule that emits a specific wavelength of light when excited by a different wavelength of light.

2.6. **Nucleotide:** A nucleoside with one or more phosphate groups linked to the 5' carbon of the pentose sugar. Ribose-containing nucleosides include ribonucleoside monophosphate (NMP), ribonucleoside diphosphate (NDP), and ribonucleoside triphosphate (NTP). When the nucleoside contains the sugar deoxyribose, the nucleotides are called deoxyribonucleoside mono-, di-, or tri-phosphates (dNMP, dNDP, or dNTP). A building block of DNA and Ribonucleic Acid (RNA). The nucleotides are adenine (A), cytosine (C), guanine (G), thymine (T), and uracil (U).

2.7. **Polymerase Chain Reaction (PCR):** A powerful method for amplifying specific DNA segments which exploits certain features of DNA replication. For instance, replication requires a primer and specificity is determined by the sequence and size of the primer. The method amplifies specific DNA segments by cycles of template denaturation; primer addition; primer annealing and replication using thermostable DNA polymerase. The degree of amplification achieved is set at a theoretical maximum of 2N, where N is the number of cycles, e.g. 20 cycles gives a maximum theoretical 1, 048, 576-fold amplification. In addition to primers and DNA polymerase, PCR reactions must contain template DNA (the DNA to be amplified) and the DNA "building blocks," deoxynucleotide triphosphates (dNTPs, which include dATP, dTTP, dGTP, and dCTP).

2.8. **Primer:** Short preexisting polynucleotide chain to which new deoxyribonucleotides can be added by DNA polymerase. This DNA or RNA fragment anneals to a template of single-stranded DNA, providing a 3' hydroxyl end from which DNA polymerase extends a new DNA strand to produce a duplex molecule.

2.9. **STR (Short Tandem Repeat**): Microsatellite DNA containing 2-5 bp repeats and found throughout the genome of a species.

2.10. **Template:** Any RNA or single-stranded DNA molecule upon which a complementary nucleotide strand is synthesized. DNA stores coded information and acts as a model or template from which information is copied into complementary strands of DNA or transcribed into messenger

RNA (mRNA). In the context of STR analysis, the template is single-stranded DNA, including heat-denatured double stranded DNA.

3. DOCUMENTS/RECORDS

3.1. Document any changes made in the protocol with an explanation and submit this information along with the analyzed data.

4. EQUIPMENT/SUPPLIES/REAGENTS

4.1. EQUIPMENT

4.1.1. PCR Cabinet (with UV light recommended for decontamination)

4.1.2. Micropipette (2 µL, 10 µL, 20 µL, 100 µL, 200 µL, 1000 µL)

4.1.3. Multi 8-channel pipette (10 µL maximum volume)

4.1.4. Appropriate pre-sterile filter micropipette tips (ShaftGuard preferred)

4.1.5. Vortex mixer

4.1.6. Microcentrifuge with adaptors for 1.5 mL tubes and 8-strip 0.2 mL PCR tubes

4.1.7. Eppendorf centrifuge, with 96-well plate adapters (or equivalent)

4.1.8. Applied Biosystem Veriti™ Thermocycler (or equivalent)

4.1.9. Applied Biosystem® ABI 3500xL Genetic Analyzer (or ABI 3100, 3130xL, 3730, 3730xL)

4.1.10. Applied Biosystem 36 cm or 50 cm capillary array

4.2. SUPPLIES

4.2.1. 1.5 mL and 2 mL sterile microcentrifuge tubes and tube rack

4.2.2. 0.2 mL PCR tubes/caps (or equivalent) and tube rack

4.2.3. MicroAmp Optical 96-well plate (ABI Cat. no. N801-0560), or equivalent

4.2.4. MicroAmp Tray and Retainer (ABI Cat. no. 403081), or equivalent

4.2.5. Plate septa 96-well (ABI Cat. no. 4315933), or equivalent

4.3. REAGENTS (excluding Mouse STR Validation Kit)

4.3.1. 10 % bleach and 70 % ethanol (or equivalent)

4.3.2. PCR grade water

4.3.3. DS-33 Matrix Standard Kit (Dye Set G5) (ABI/Thermo Fisher, Cat. No. 4345833)

4.3.4. GeneScan® (GS) LIZ600 v2.0 internal size standard (ABI/Thermo Fisher, Cat. No. 4408399)

4.3.5. Hi-Di Formamide (ABI/Thermo Fisher, Cat. no. 4311320)

4.3.6. Anode buffer (ABI/Thermo Fisher, Cat. no. 4393927), or equivalent

4.3.7. Cathode buffer (ABI/Thermo Fisher, Cat. no. 4408256), or equivalent

4.3.8. POP-4 (POP-4 ABI/Thermo Fisher, Cat. no. 4393710) or POP-6 or POP-7 polymer

5. <u>SAFETY PRECAUTIONS AND STORAGE</u>

5.1 Use good laboratory practices at all times while working. Perform PCR reactions in pre-PCR area and in a PCR hood.

5.2 Refer to the Material Safety Data Sheet (MSDS) for the handling of all reagents prior to use and storage.

5.3 Storage Conditions:

5.3.1 Store all kit components at -20 ˚C upon receipt.

5.3.2 Once kit components are thawed, store Qiagen Type-It Microsatellite PCR Mastermix (MM) at -20 °C. All DNA samples (M01-M50), Calibrants (C1-C5), Human Control (H1), and Mouse Multiplex Primers (P21) should be stored at 4 °C.

5.3.3 Mouse Multiplex Primers (P21) are light sensitive, store in the dark at 4 °C.

5.3.4 Store Pre- and Post-amplification reagents and products, separately.

6. <u>PROCEDURE</u>

6.1. PCR amplification of STR markers in DNA samples

6.1.1. Prepare PCR amplification work area (turn on the hood for ten minutes, clean and decontaminate hood).

6.1.2. Prepare samples to be tested and create a sample sheet that will include negative control (molecular grade water), human DNA control (H1), Calibrants (C1-C5), and all mouse DNAs (M01-M50). Label PCR tubes accordingly.

6.1.3. Prepare the PCR Master Mix for 61 samples (50 mouse DNA samples, 5 Calibrants, 1 human DNA control, 1 negative control (water), and 4 extra reactions for pipetting error.

6.1.4. Prepare PCR Master Mix in a 2 mL microcentrifuge tube using Table 2.

! CAUTION ***Qiagen Mastermix (2X) is very viscous. Completely thaw and warm the Mastermix to room temperature and apply pipetting technique applicable for viscous liquid (aspirate and dispense liquid <u>slowly</u>).***

Table 2. Preparation of Master Mix for 61 PCR Reactions			
**PCR Master Mix Reagent**	**Base Volume (µL)**	**Multiplication factor (number of reactions)**	**Volume Used (µL)**
Water, PCR Grade	8	61	488
Qiagen Mastermix (MM)	12.5	61	762.5
Mouse Multiplex (P21)	2.5	61	152.5
TOTAL VOLUME	23	61	1403

6.1.5. Vortex well, centrifuge and transfer 23 µL of the prepared PCR Master mix into each PCR tube.

6.1.6. Add 2 µL of each sample (2 ng/µL mouse DNA, 2 ng/µL Calibrants, 2 ng/µL human DNA control or negative control- water) to PCR tube using an 8-channel pipette (or equivalent).

6.1.7. Cap (seal) PCR tubes, briefly vortex, centrifuge, and place in the thermal cycler.

6.1.8. Decontaminate PCR hood with UV light (or 10 % bleach followed by 70 % ethanol).

6.1.9. Return all reagents to their proper storage place (all DNAs and P21 primers at 4 °C, and MM at -20 °C).

6.1.10. Run the thermal cycler with the cycling programs shown in Table 3.

Table 3. Cycling Conditions for PCR			
**Step**	**Temperature**	**Time**	**Cycles**
Denaturation	95 °C	5 minutes	
Amplification	95 °C	30 seconds	29 cycles
	59 °C	90 seconds	
	72 °C	30 seconds	
Soak (to promote complete adenylation)	60 °C	90 minutes	

6.1.10 When PCR run is complete, prepare the samples for capillary electrophoresis (CE); or place the reaction tubes in post-PCR refrigerator (at 4 °C) for future analysis.

6.2. Separation and detection of STR amplicons by capillary electrophoresis (CE)

6.2.1. **ABI 3500xL (or 3100, 3130xL, 3730, 3730xL) Genetic Analyzer Set-up**

6.2.1.1. Prepare Master Mix for 61 samples in a 1.5 mL centrifuge tube according to Table 4.

**Table 4. Assembling of Master Mix**			
**Master Mix Reagent**	**Base Volume (µL)**	**Multiplication Factor (number of reactions)**	**Volume Used (µL)**
Hi-Di Formamide	9.5	61	579.5
GeneScan 600 v2 LIZ internal size standard	0.5	61	30.5
**TOTAL VOLUME**	**10**	**61**	**610**

6.2.1.2. Vortex Master Mix, centrifuge and pipet 10 µL of Master Mix to the proper wells of the optical 96-well reaction plate. See plate layout below.

	1	2	3	4	5	6	7	8	9	10	11	12
A	M01	M09	M17	M25	M33	M41	M49	NTC				
B	M02	M10	M18	M26	M34	M42	M50					
C	M03	M11	M19	M27	M35	M43	C1					
D	M04	M12	M20	M28	M36	M44	C2					
E	M05	M13	M21	M29	M37	M45	C3					
F	M06	M14	M22	M30	M38	M46	C4					
G	M07	M15	M23	M31	M39	M47	C5					
H	M08	M16	M24	M32	M40	M48	H1					

- Mouse DNA samples: M01-M50 (orange)
- Calibrant DNA samples: C1-C5 (blue)
- Human DNA control: H1 (yellow)
- No Template Control (negative Ctrl-water): NTC (gray)

6.2.1.3. Add 11 µL of Hi-Di formamide (neat) to each remaining well within an injection, according to the plate layout sheet. For example: for a 24-capillary array, each injection is 24 wells (add 11 µL of Hi-Di to the remaining wells in columns 8 and 9 to complete the total wells needed for 3 injections). Each well must contain either a sample/Hi-Di/Internal size standard, or Hi-Di (neat) per injection.

6.2.1.4. Add 1 µL of amplified PCR product to each corresponding well using a multi 8-channel pipette (or equivalent), as indicated in the plate layout.

6.2.1.5. Seal the 96-well plate with septa and label the plate appropriately.

6.2.2. **3500xL (or 3100, 3130xL, 3730, or 3730xL) Genetic** A**nalyzer Run**

6.2.2.1. Briefly centrifuge the 96-well plate at 2000 x g for 1 min and place the plate into the STR cassette base (provided with the instrument).

6.2.2.2. Confirm that the Genetic Analyzer instrument has sufficient cathode and anode buffers, polymer, and runs available on the array to complete all injections.

6.2.2.3. Confirm that a five-dye matrix has been previously established under the G5 filter with the following dyes: 6FAM, VIC, NED, PET, and LIZ according to the manufacturer's instructions.

Figure 1: Set-up dye set

6.2.2.4. Setup an Instrument Protocol using the parameters below (**for ABI 3500xL and 3730 Genetic Analyzers**):

The Instrument Protocol is named "Cell ID_15s inj". Set the capillary length (either 36 cm or 50 cm) and the polymer (either POP4, POP6, or POP7).

Set the following parameters: oven temp (60 °C), run time (1500 sec), run voltage (15 kV), pre-run time (180 sec), pre-run voltage (15 kV), injection time (15 sec), injection voltage (1.2 kV), and data delay (1 sec).

<u>NOTE:</u> For **ABI 3130 and 3100 Genetic Analyzers**, please use the following conditions:

Inject samples electrokinetically for 10 sec at 3 kV. Separate alleles at 15 kV at a run temperature of 60°C for 1500 seconds.

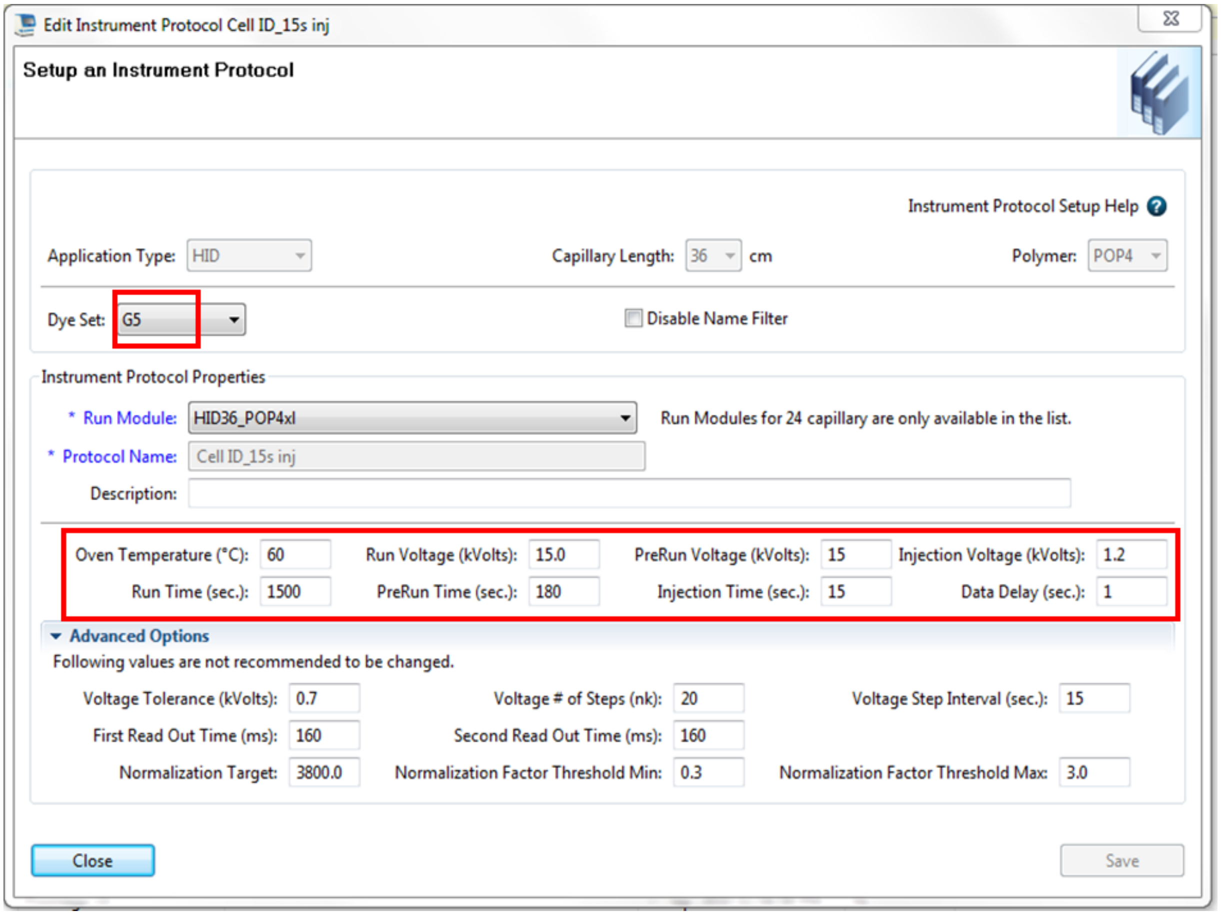

6.2.2.5. Setup a QC Protocol using the parameters below:

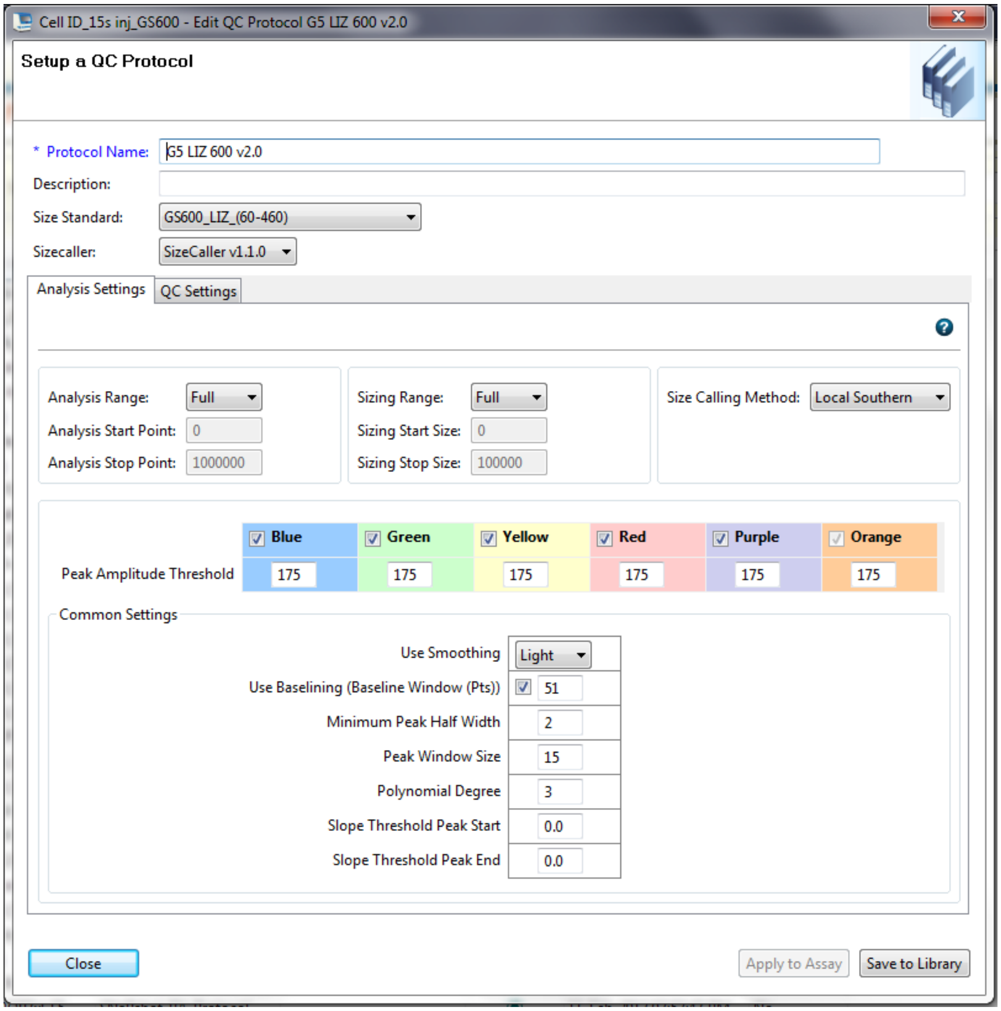

6.2.2.6. Setup an Assay Protocol using the parameters below:

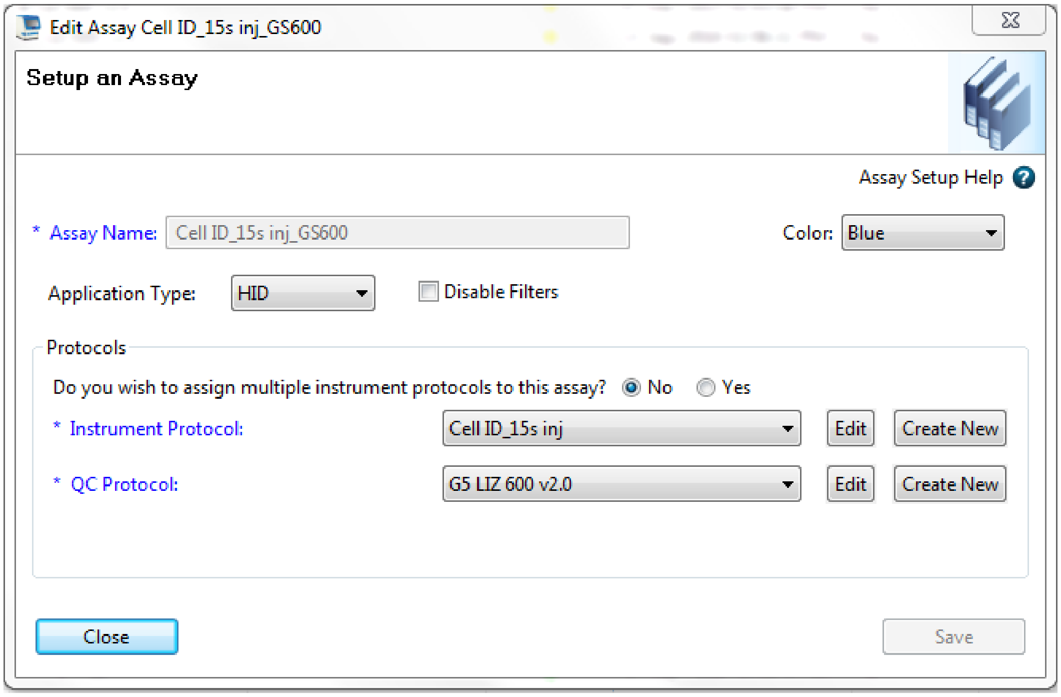

6.2.2.7. Assign sample names, assay, file name convention, and results group to your plate.

6.2.2.7.1. Assign sample names to each well by using the information in Figure 1.

6.2.2.7.2. Select the assay called "Cell ID_15s inj_GS600".

6.2.2.7.3. Select the file name convention "<Samplename>_<well>_<capillary number>".

6.2.2.7.4. Select the results group (we use Identifiler, <Results group name> <IP name>).

6.2.2.8. Highlight the samples on the plate and select the assigned assay, file name convention, and results group to link to the samples.

6.2.2.9. Select "Link Plate".

6.2.2.10. Select "Run".
