# Supplementary material for: Interlaboratory study to validate a STR profiling method for intraspecies identification of mouse cell lines
Source: PLoS One. 2019 Jun 20;14(6):e0218412. doi: 10.1371/journal.pone.0218412 (PMC6586308; doi:10.1371/journal.pone.0218412)
Supplement: S2 File — Data Analysis instructions for GeneMapper ID-X Software; GeneMapper IDv3.2.1, GeneMapper v4.0, v4.1, v5.0 Software platforms. (PDF) [file pone.0218412.s002.pdf]

PROTOCOL 2: DATA ANALYSIS: GeneMapper® ID-X Software; GeneMapper® ID v3.2.1, GeneMapper® v4.0, v4.1, v5.0 Software

1. INTRODUCTION/BACKGROUND

1.1. PURPOSE/SCOPE

The purpose of this protocol is to provide detailed information for data analysis and data export of mouse STR profiles using GeneMapper Software.

1.2. THIS PROTOCOL PROVIDES DETAILED INFORMATION REGARDING:

1.2.1. Importing of bins and panels, analysis methods, table settings, plot settings and size standards for the Applied Biosystems GeneMapper® ID-X Software, GeneMapper ID v3.2.1, or GeneMapper® Version 4, 4.1, or 5.0 Software (files will be provided electronically by NIST).

1.2.2. Adjustment of bins and panels to the appropriate fragment lengths based on data collected using the provided calibrant samples.

1.2.3. Export data file after analysis is complete.

1.3. EACH CONSORTIUM MEMBER WILL BE PROVIDED WITH:

1.3.1. Five calibrant DNA samples

1.3.1.1. Calibrants 1-4 contain mixtures of 2 or more mouse DNAs to obtain the most allele coverage possible; Calibrant 5 is a single sample.

1.3.1.2. Calibrants will be used to generate fragment lengths obtained by genotyping the samples using the mouse multiplex PCR assay.
Note: All alleles present in the calibrant samples have been sequenced).

1.3.2. STR profiles for the calibrant DNA (Table 1) and their associated electropherograms (Figures 1-5) will be provided electronically.

1.3.2.1. These profiles and fragment lengths were obtained in NIST laboratories using an ABI 3500xL, POP-4 polymer, and a 36 cm array.

1.3.2.2. Fragment length values may vary if instruments and consumables are used other than what is listed above.

1.3.3. An Excel file of a fill-in table (Table 2) containing all known alleles for 19 mouse STR markers will be provided electronically.

1.3.3.1. Fragment lengths obtained from genotyping the calibrant samples using the mouse multiplex PCR assay will be recorded in Table 2.

1.3.3.2. Dataset from the calibrants will be used to adjust the bins and panels.

2. DOCUMENTS/RECORDS

Document any changes made in the protocol with an explanation, and submit this information along with the analyzed data.

3. EQUIPMENT/SUPPLIES

3.1. Computer with Windows Software

3.2. GeneMapper® Software (one of the following software platforms)

3.2.1. GeneMapper® ID-X or

3.2.2. GeneMapper® ID v3.2.1 or

3.2.3. GeneMapper® v4.0, 4.1, 5.0 Software

4. IMPORTING BINS AND PANELS

4.1. IMPORTING PANELS AND BIN SETS

4.1.1. Open GeneMapper® Software and log in.

4.1.2. Download the files for bins and panels, provided by NIST, to a desktop with GeneMapper Software. Select “Tools” and then “Panel Manager” from the drop-down menu. Click on “Panel manager” in the navigation panel to the left of the screen.

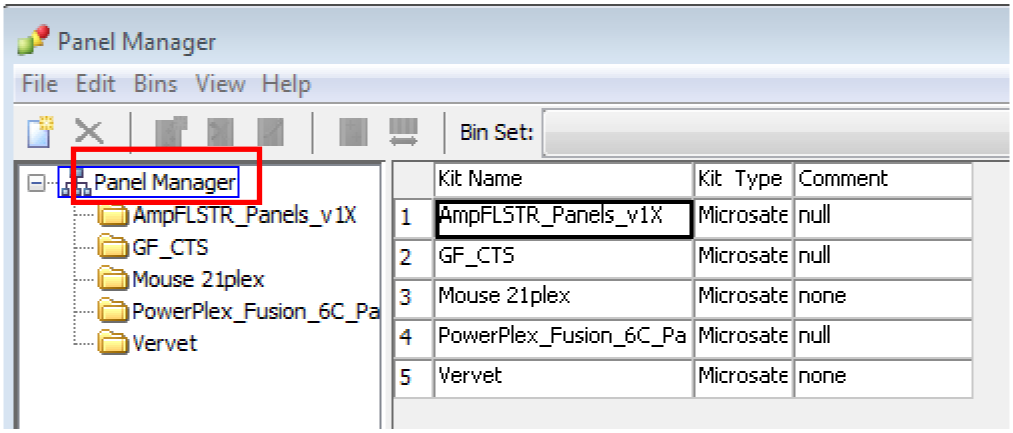

4.1.3. Select “File” and then “Import Panels”. Navigate to the desktop to select (Mouse 21plex-Panels), then click “Import”. Click “OK”.

4.1.4. Select the “Mouse 21plex” folder under “Panel Manager” in the navigation panel to the left of the screen below.

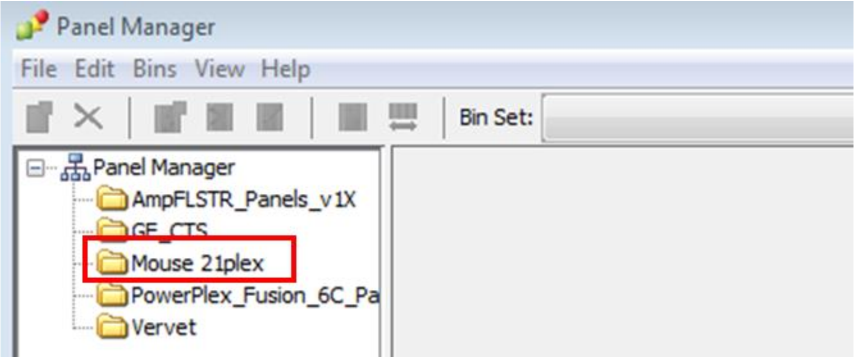

4.1.5. Go to "File" and "Import Bin Set". Navigate to desktop to select the provided file called "Mouse 21plex_bins" and select "Import", then click "OK".

4.1.6. The new bins and panels will now be visible in the "Panel Manager".

4.2. IMPORTING ANALYSIS METHODS, TABLE SETTINGS, PLOT SETTINGS AND SIZE STANDARD

4.2.1. In the toolbar, go to "Tools" and then select "GeneMapper® Manager" from the drop-down menu.

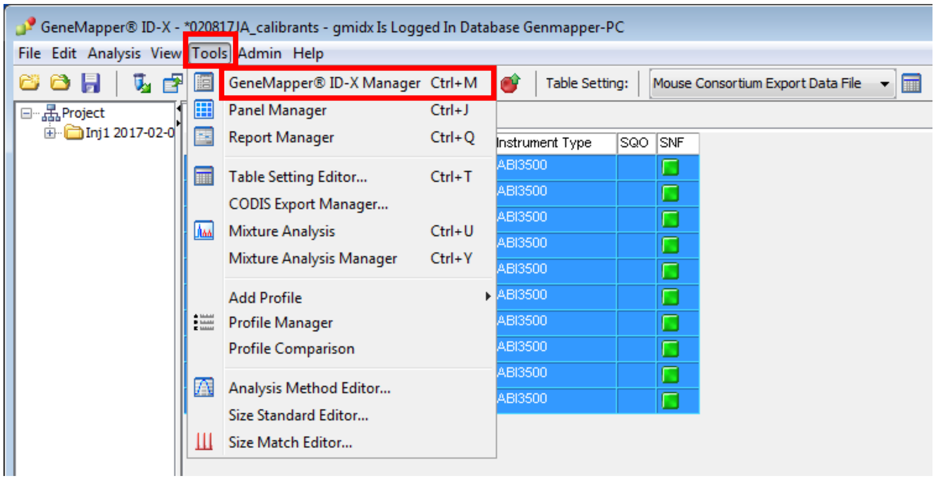

4.2.2. Select the “Analysis Methods” tab and click “Import”.

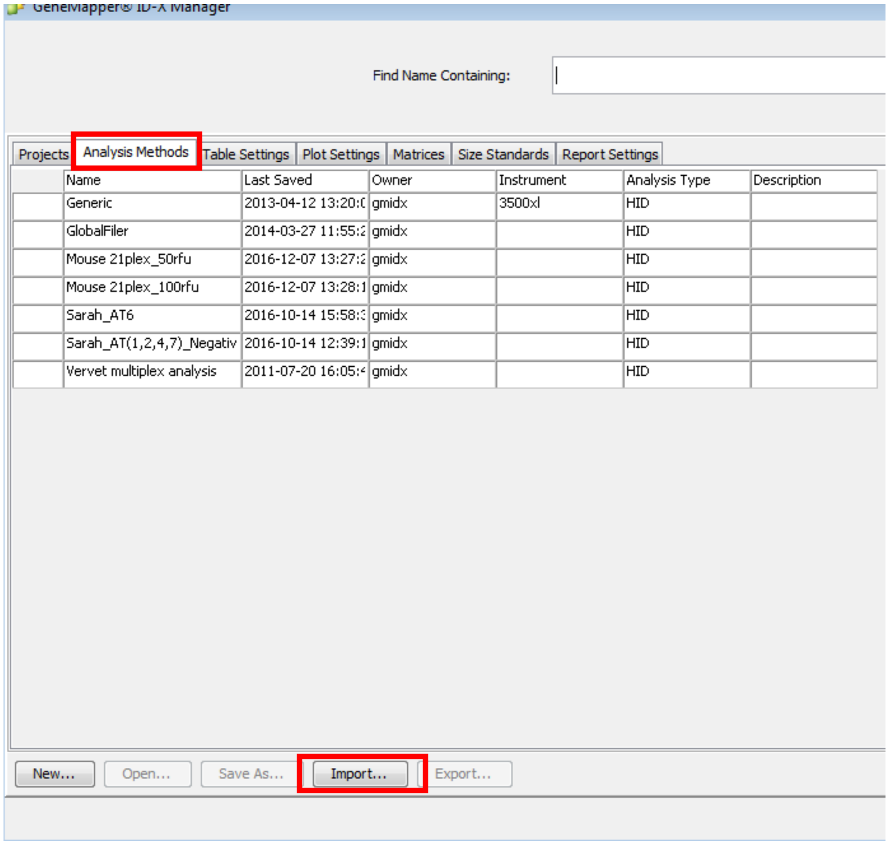

4.2.3. Navigate to desktop to select the provided file called “Analysis Method” (this file actually contains two analysis methods: Mouse21plex_50rfu and Mouse21plex_100rfu). Click “Import” and select “Done”.

4.2.4. Go to the “Table Setting” tab and click “Import”. Navigate to desktop to select the provided file called “Mouse Consortium Export Data File” and click “Import”, then click “Done”.

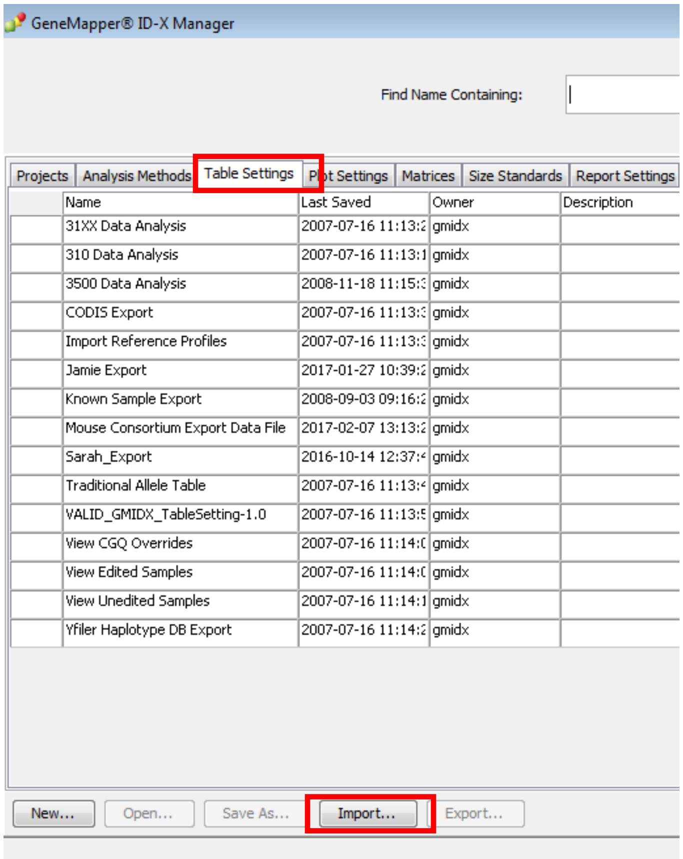

4.2.5. Go to the "Plot Settings" tab and click "Import". Navigate to desktop to select the provided file called "Traditional Genotype Plot" and click "Import", then click "Done".

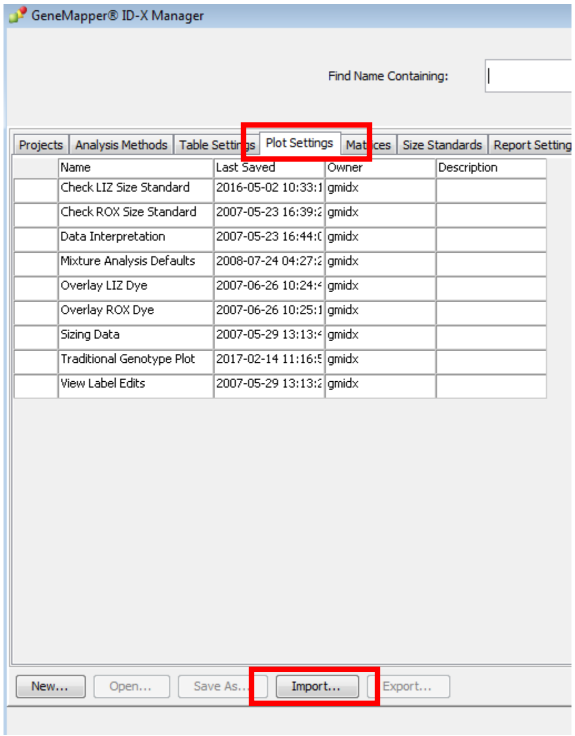

4.2.6. Go to the “Size Standards” tab and click on “Import”. Navigate to desktop to select the provided file “GS600_LIZ”. Click “Import”. Click “Done”.

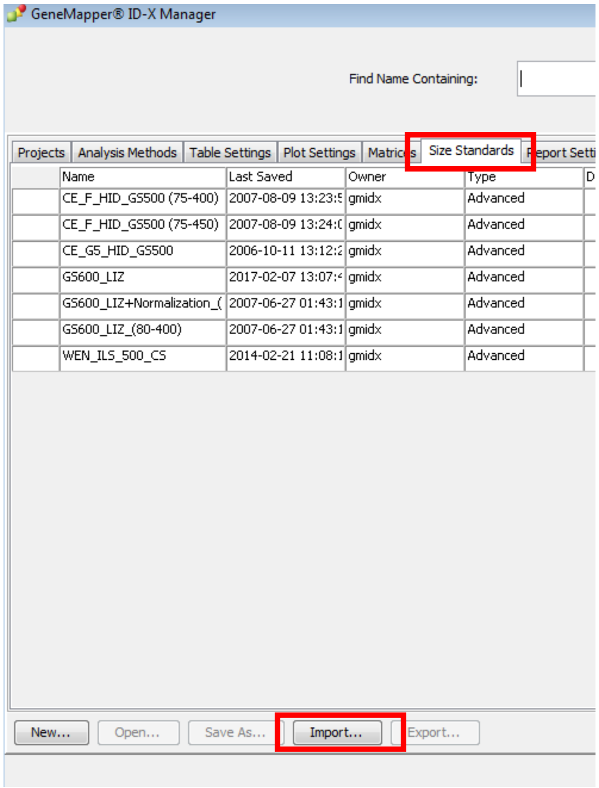

5. ADJUSTING OF BINS AND PANELS

The current bins and panels reflect fragment lengths using the ABI 3500xL, POP-4 polymer, and a 36 cm array in our lab. Different instruments, DNA size standards, polymers, and arrays may result in different fragment lengths of the alleles (size in base pairs). Calibrant samples are provided with known repeats based on sequence data. The bins and panels may need to be adjusted to reflect differences in fragment length based on instrumentation and consumables, other than what is listed above.

5.1. STR ANALYSIS OF FIVE CALIBRANTS

5.1.1. Complete PCR and fragment analysis of the five calibrant DNA combination samples by following **Protocol 1: PCR and Genetic Analyzer Sample Preparation**.

5.1.2. Import data from the Genetic analyzer onto a desktop with GeneMapper® Software.

5.1.3. Add samples to project (test tube icon; or choose “Edit”, then “Add Samples to Project”).

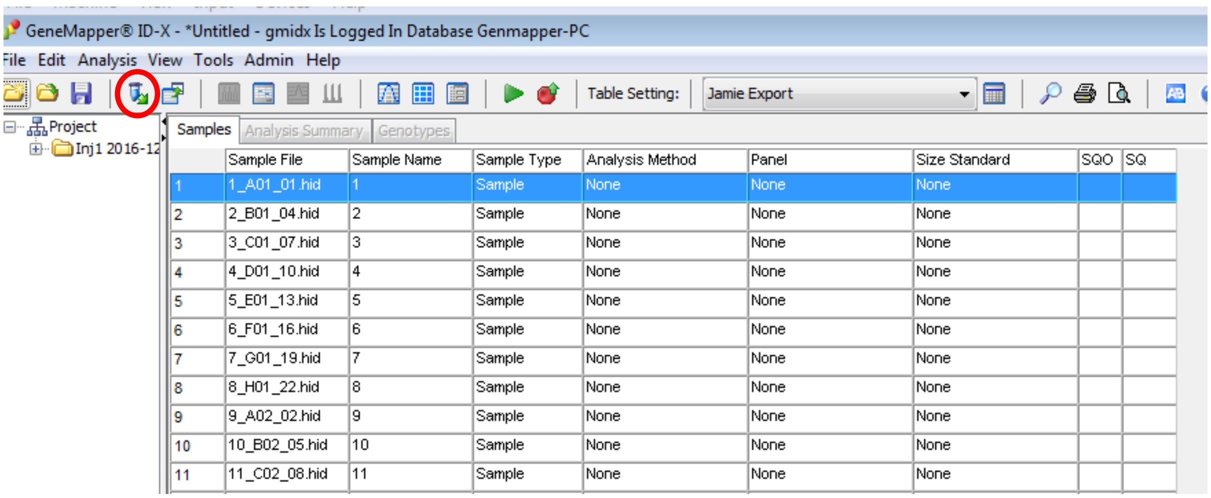

5.1.4. Use the drop-down menu under "Analysis Method" and select "Mouse 21plex _50rfu".

5.1.5. Use the drop-down menu under "Panel" and select "Mouse 21plex".

5.1.6. Use the drop-down menu under "Size Standard" and select "GS600_LIZ".

5.1.7. Select the parameter (e.g., Size Standard), mark the top of the column using the drop-down menu, and press "Ctrl + D" or click "Edit" and "Fill Down".

NOTE: Use the "Fill Down" function in order to analyze all samples with the same parameters.

5.1.8. Click the green arrow icon to start analysis.

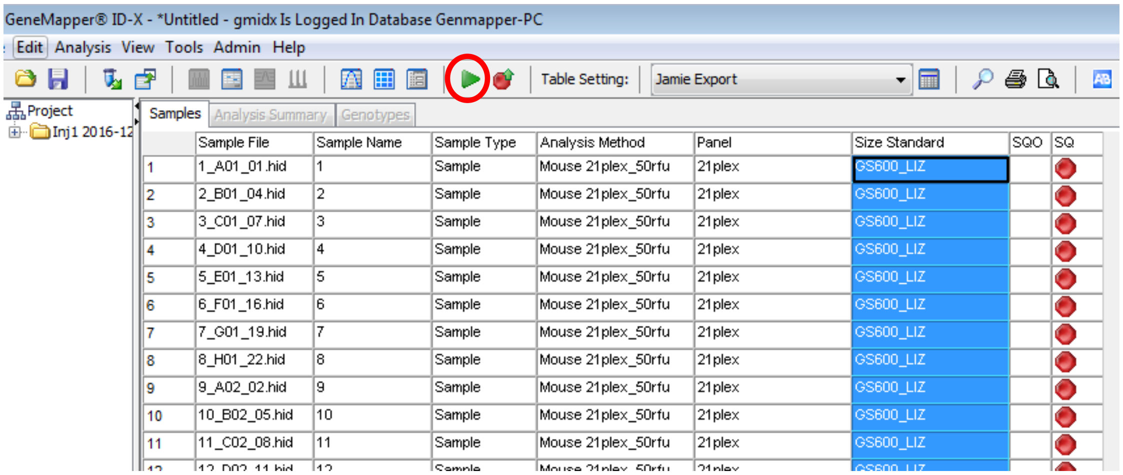

5.1.9. When prompted with the "Analysis Requirements Summary", select "Continue analysis", and click "OK".

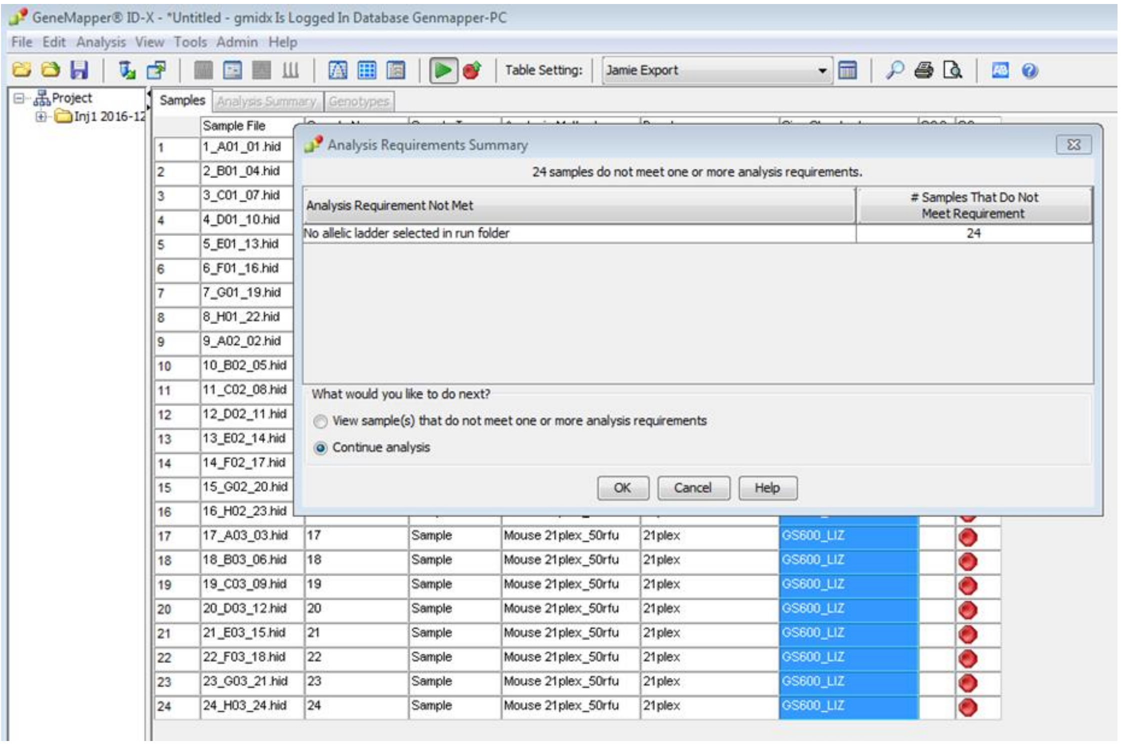

5.1.10. Analysis will begin after the project is saved. Save the project by using the file convention Date_Institution_Operator. (Ex. 031617_NIST_Almeida)

5.1.11. Click on the value under “Total # of Samples” highlighted in blue at the bottom on the screen, or choose the “Samples” tab to review the analyzed data.

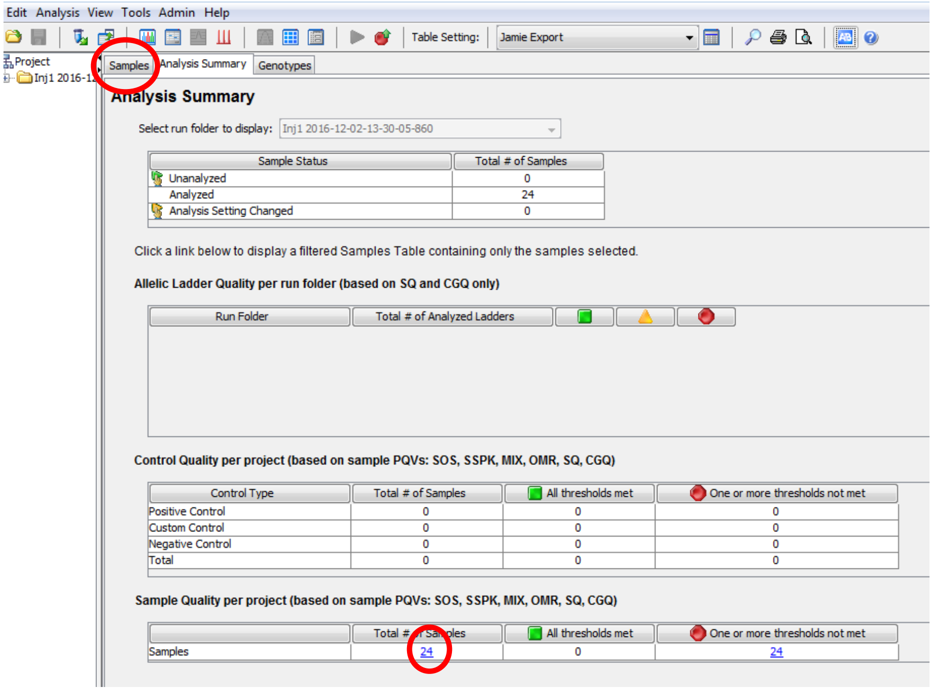

5.1.12. To visualize and review the analyzed data, highlight to select samples and select the "Display Plot" icon to visualize electropherograms.

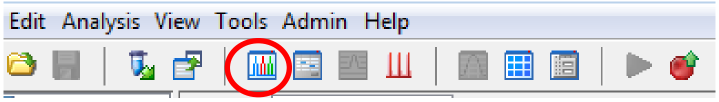

5.1.13. Use the drop-down menu to select the default Plot Setting: "Traditional Genotype Plot".

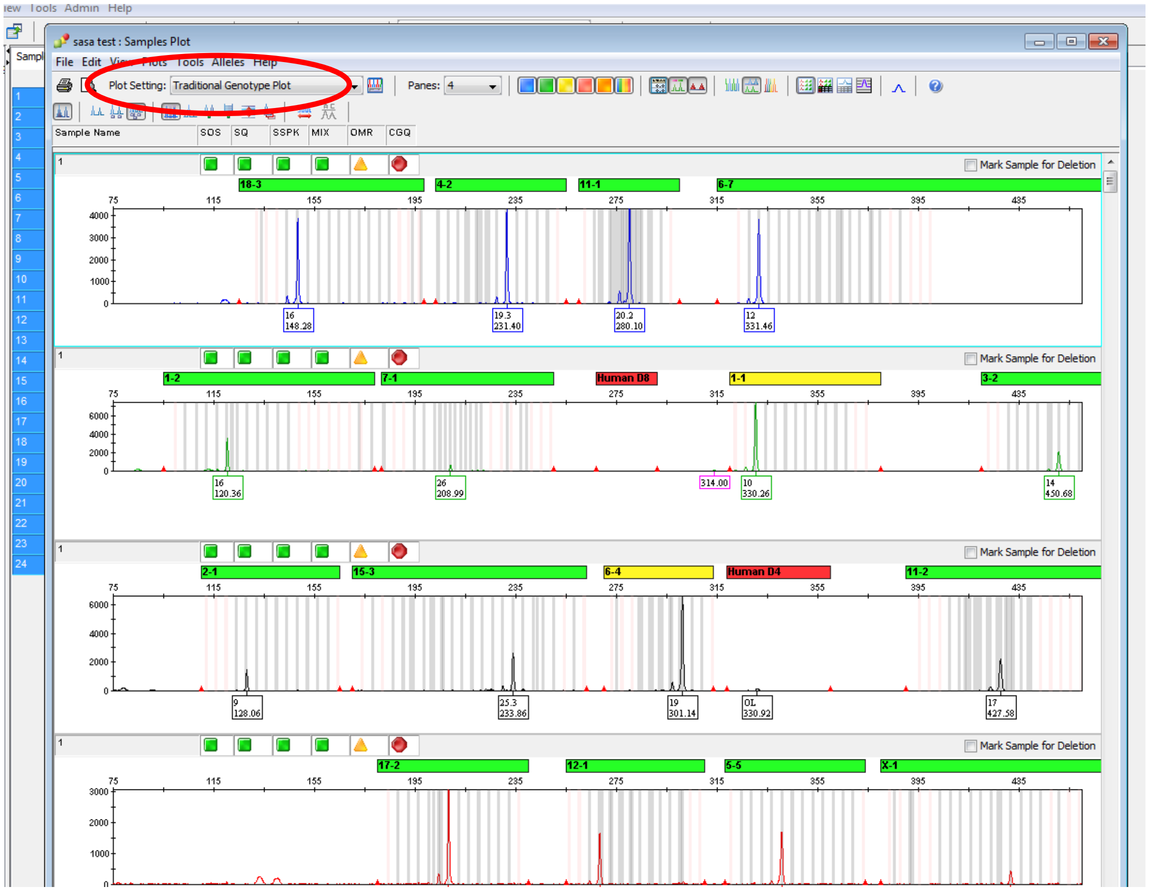

5.1.14. If the size standard is flagged, check the quality of the size standard. Note: This may be due to a poor injection or blown out signal.

5.1.15. Reinject the sample if the problem is due to a bad injection. If the sample is blown out and has bleed through, check the sizing peaks (these can be overridden if the peaks look ok). The size standard data can be accessed using the "Size Match Editor" icon (circled below), or by selecting "Tools" and "Size Match Editor" from the drop down menu.

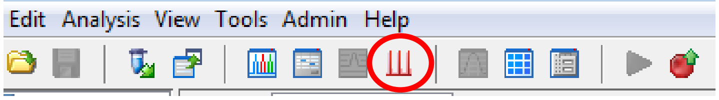

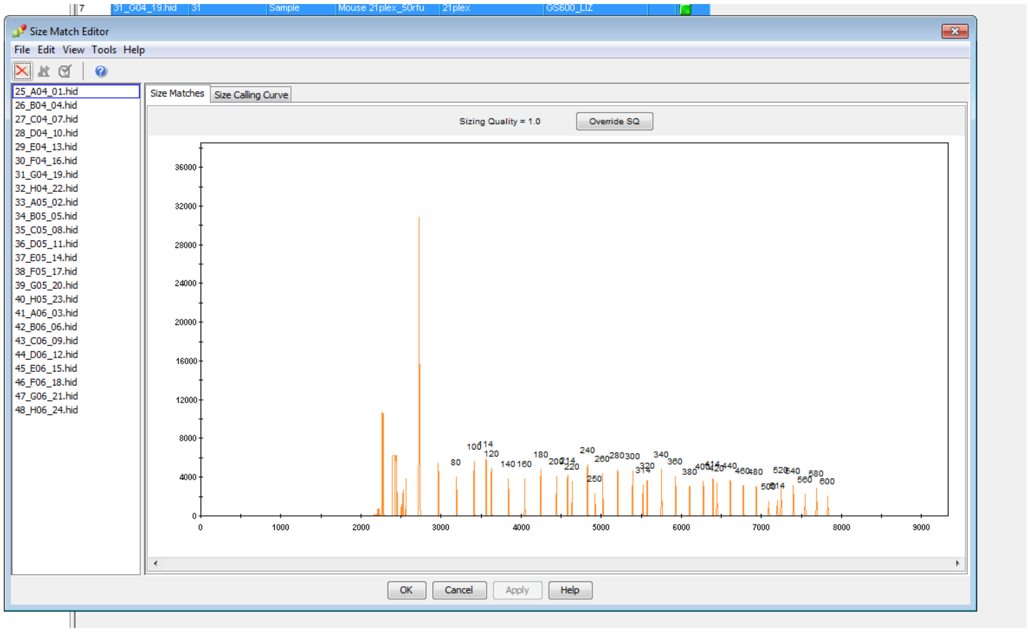

6. CALIBRANT DATA IMPORT

6.1. IMPORTING OF CALIBRANT DATA

6.1.1. Complete Table 2 (Allele Distribution for 19 STR Markers) by filling in the fragment length data obtained for each allele at each STR marker using the calibrant samples. Extrapolate fragment length values for alleles not represented in the calibrant samples until Table 2 is completed.

Example: STR marker 18-3

	A	B	C	D	E	F	G	H	I	J	K	L	M	N	O	P	Q
1	**Allele Distribution for 19 STR Markers**																
2	Marker										Alleles						
3	18-3																
4	FR3pig																
5																	
6																	
7																	
8	repeat	12	12.2	13	14	15	16	17	18	19	20	21	22	23	26	27	

For example: STR analysis of five calibrants will cover six alleles (repeats) for 18-3 Marker: 16, 17, 18, 19, 21 and 22. Allele 20 is unrepresented. The fragment length for the allele 20 will be estimated by extrapolating from the fragments length of allele 19 (fragment length: 160.8 bp) or 21 (168.8). Since each allele number represents tetranucleotide repeats, one allele will differ from the next one by 4 bp, and the 20 will be estimated to have 164.8 bp. Therefore, the fragment length of the allele 20 will be 4 bp more from allele 19 and 4 bp less than allele 21. For microvariants (ex. 20.1) the fragment length would be extrapolated by one base more than the fragment length for allele 20 and it will be 165.8 bp. Note that the sizing may vary slightly (165.8 bp may be 165.6 bp) due to migration effects. The left and right bin

offset is set to 0.4 bp of the mean fragment length for each bin and should capture these slight variations in fragment length.

No. Repeats	Fragment Length (bp)
20	164.8
20.1	165.8
20.2	166.8
20.3	167.8
21	168.8
21.1	169.8

6.1.2. Once Table 2 is complete, adjust all bins and panels to the fragment lengths recorded in Table 2 (see instructions below).

6.2. ADJUSTING BINS AND PANELS

6.2.1. Go to “Tools”, select “Panel Manager”, and click on the panel for the Mouse 21plex. The STR markers will appear under the folder “21plex.

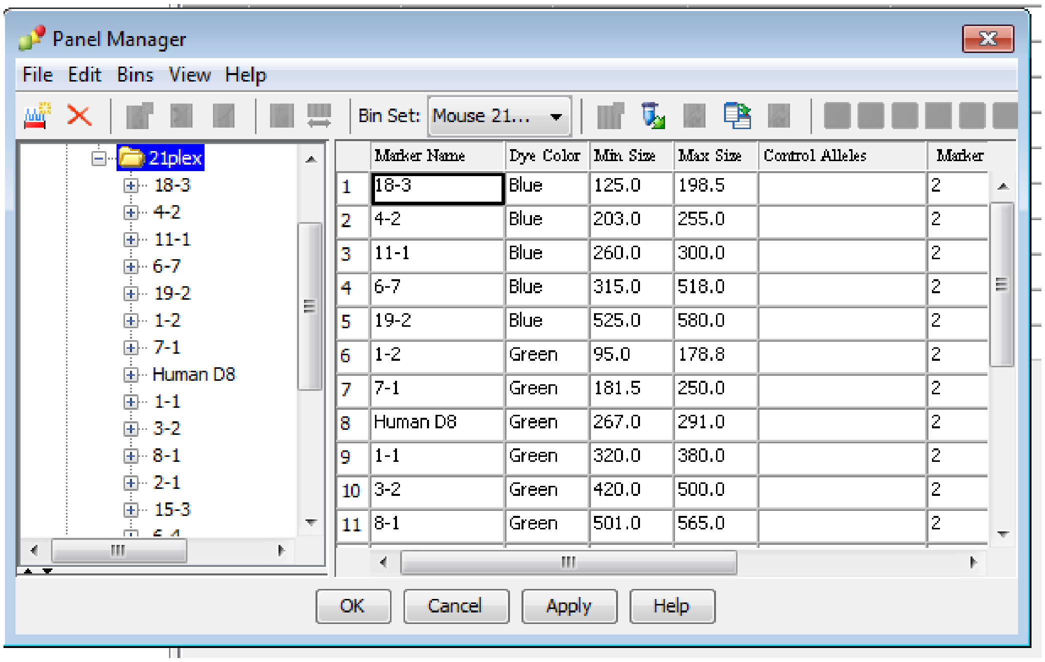

6.2.2. Click on the STR marker (ex.18-3) in the navigation part of the screen, and bins for that STR marker will appear. Select an existing bin (repeat numbers are labeled at the top of the bin) by left clicking the mouse, and then right click to Edit Bin.
NOTE: Do not change the name of the bin (this is the repeat number and it will not change).

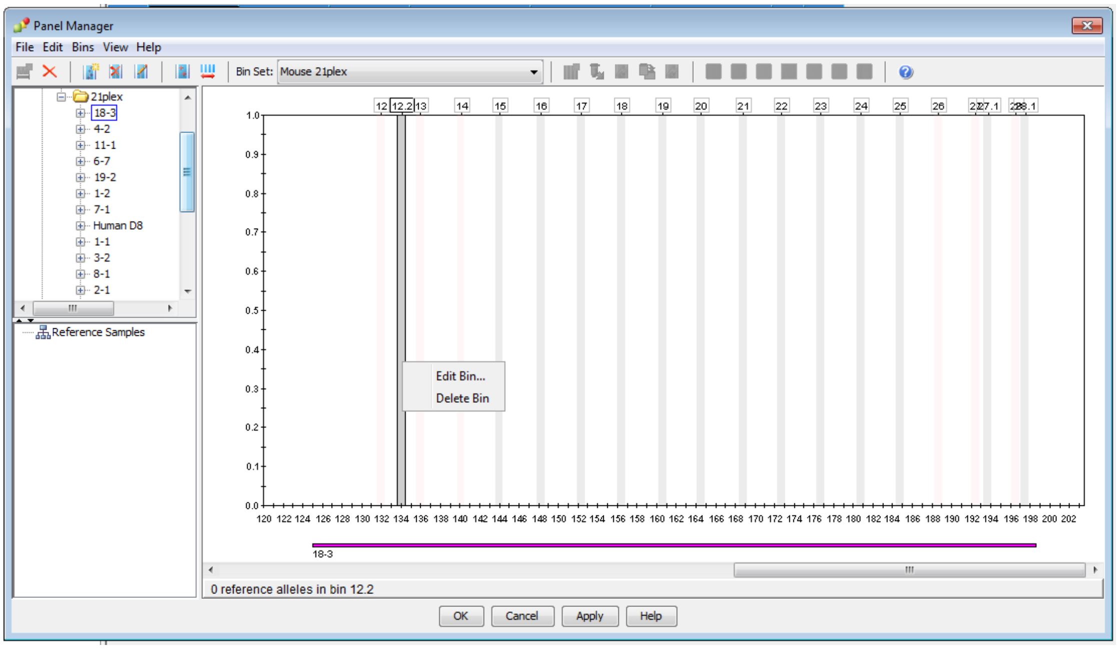

6.2.3. Select "Location" and change the fragment length to the value for that allele on Table 2. Some calibrants share alleles and their fragment lengths should be recorded and then averaged. The average value of the fragment length should be input into the "Location". Leave the right and left offset at 0.40. Select "OK".

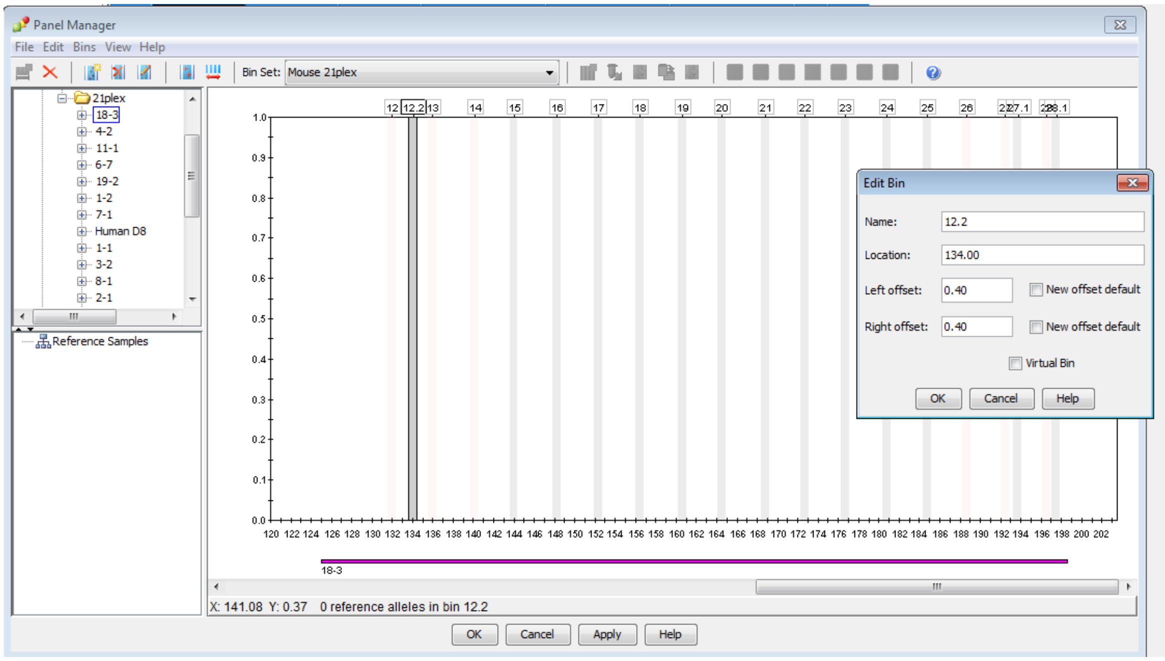

6.2.4. Repeat the same procedure to adjust bins for every allele represented on Table 2. Click "Apply" and then "OK" after any change.

6.2.5. After all bins have been adjusted and saved, reanalyze calibrant samples and confirm that all alleles fall into bins. If there is an off ladder call (OL) adjust that particular bin to accommodate the fragment length that maybe off by 0.1 bp. The bin offset can also be expanded to 0.50 instead of 0.40 for the left and right to help with this situation.

6.3. REVIEW SAMPLE DATA

6.3.1. **Off-ladder allele**
Peaks labeled with OL (Off-Ladder) could not be assigned to an allele call. These labels must be checked manually and may be deleted or redefined by clicking on them. OL calls may include bleed through, spike, dye blob, etc.

6.3.2. **Delete allele label**
In order to change the allele designation of unrealized peaks, click the icon below the peak (the icon turns bold). Open the drop-down menu by right-clicking the icon and choose "Delete Allele".

7. EXPORTING DATA FILE

7.1. EXPORTING OF DATA TABLE

7.1.1. Open the project file in GeneMapper ID-X or GeneMapper ID, or GeneMapper.

7.1.2. Choose the "Mouse Consortium Data Export Table" from the "Table Setting" drop-down screen.

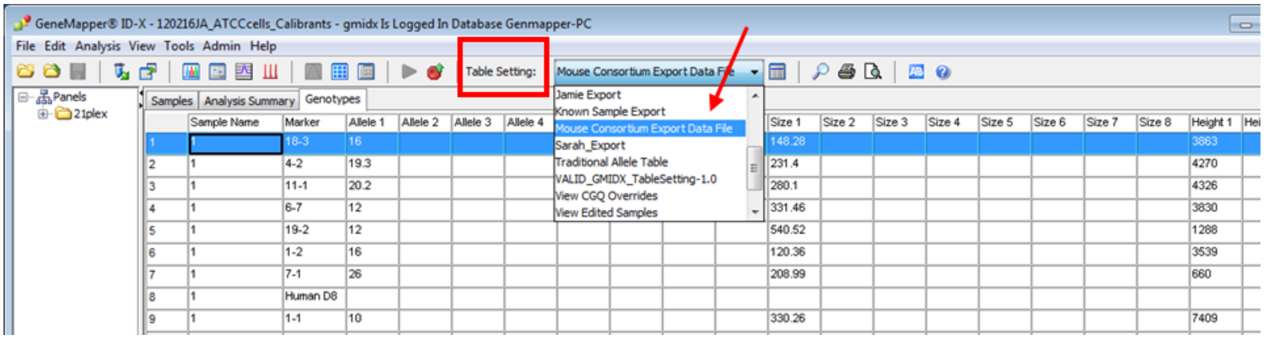

7.1.3. Click on the "Genotypes" tab (user should see Sample Name, Marker, Allele 1-8, Size 1-8, and Height 1-8).

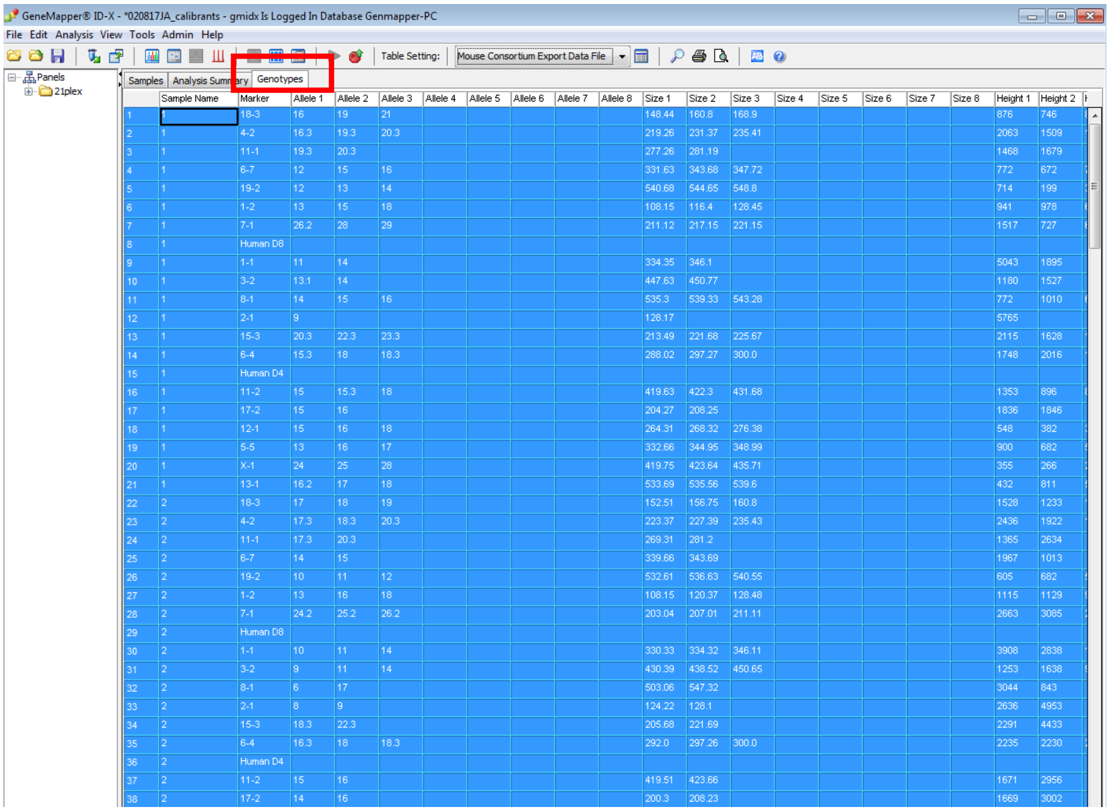

7.1.4. To select all, click "Control + A" on the keyboard (samples are sorted by sample file (default).

7.1.5. Click on the" Samples" tab.

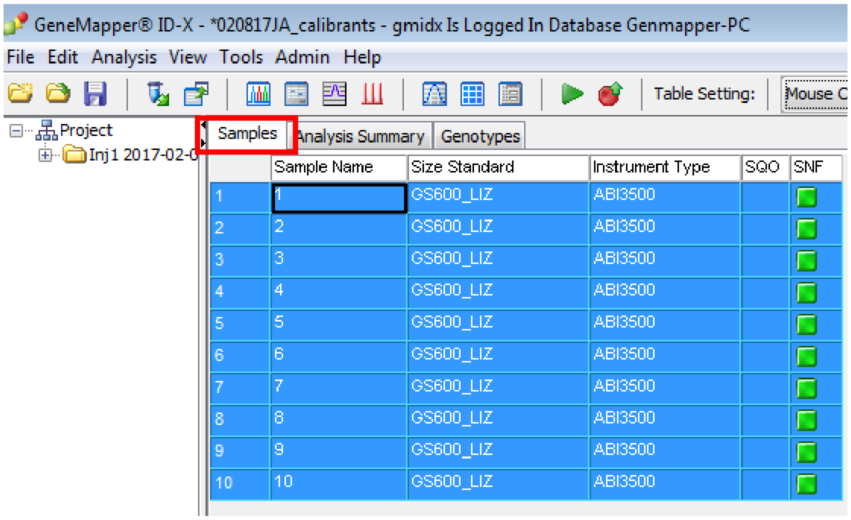

7.1.6. Click on “File”, then select “Export Combined Table”.

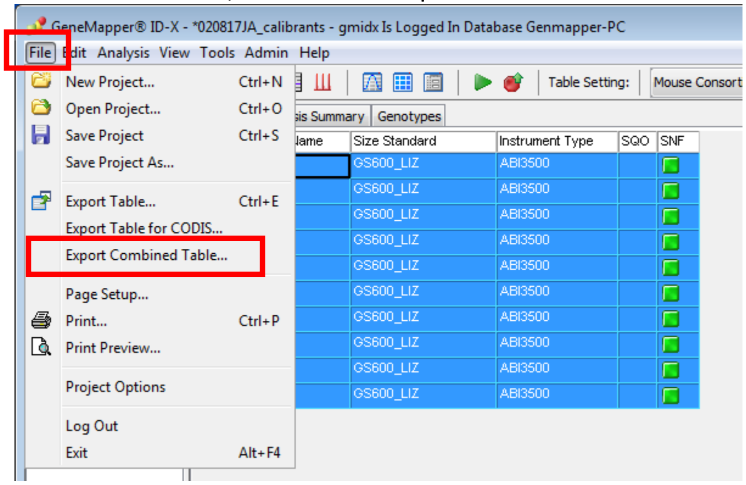

7.1.7. Under “merge” on the far right, click on “One line per sample”
NOTE: default setting is “One line per marker”.

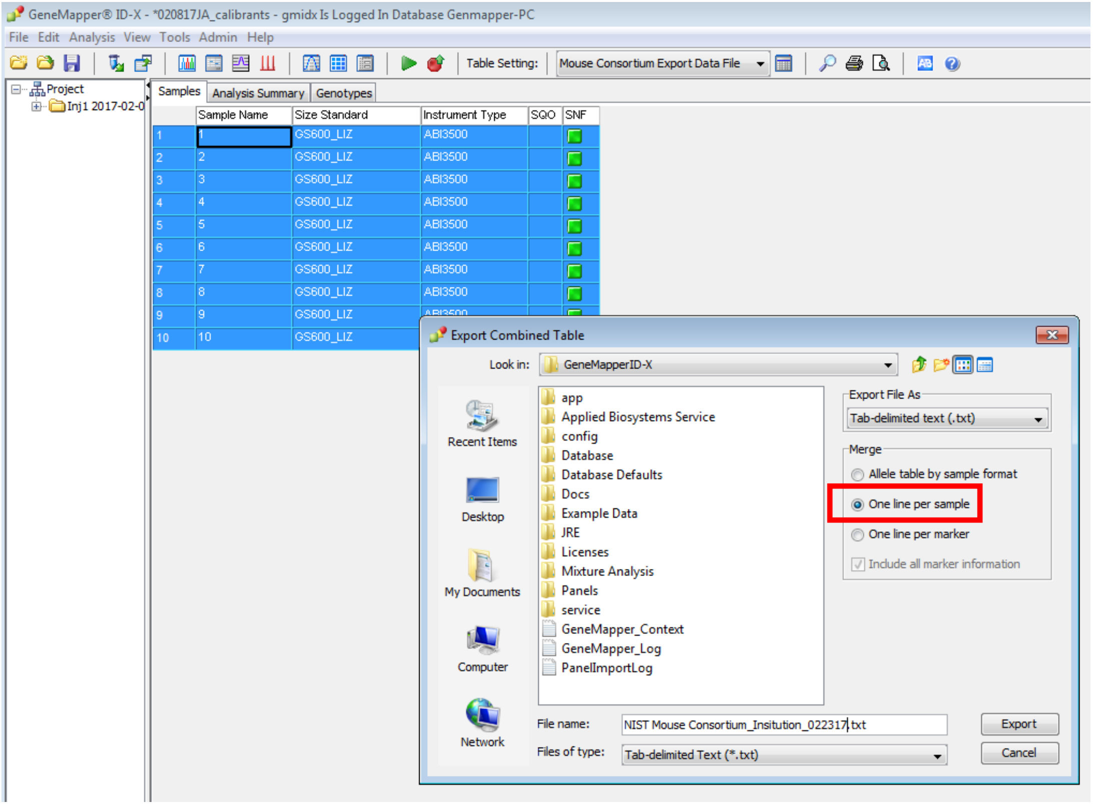

7.1.8. Name the file “NIST Mouse Consortium_Institution_date” and select the type of file saved as (*.csv) and choose folder to export to. (Ex. NIST Mouse Consortium_NIST_031617)

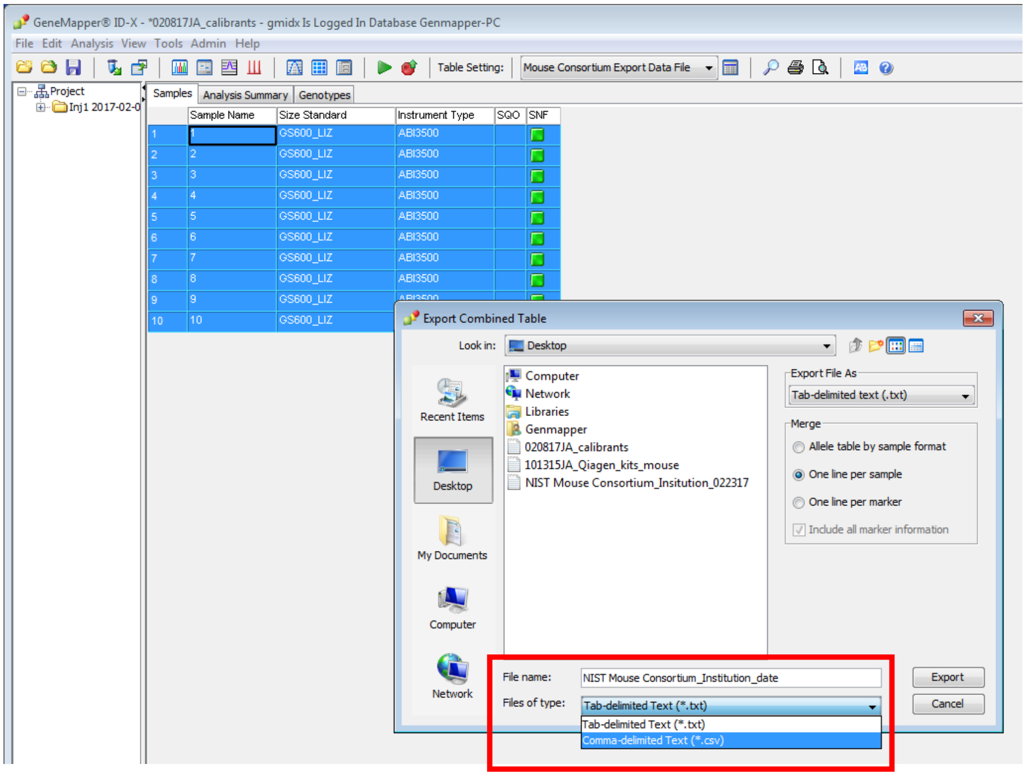

7.1.9. Click “Export”.

Send exported data file to NIST for analysis.
