# Supplementary material for: Interlaboratory study to validate a STR profiling method for intraspecies identification of mouse cell lines
Source: PLoS One. 2019 Jun 20;14(6):e0218412. doi: 10.1371/journal.pone.0218412 (PMC6586308; doi:10.1371/journal.pone.0218412)
Supplement: S3 File — Data Analysis instructions for SoftGenetics GeneMarker Software Version 1.85; Version 2.6.3 or Version 2.7.6 platforms. (PDF) [file pone.0218412.s003.pdf]

PROTOCOL 3: DATA ANALYSIS: SoftGenetics GeneMarker® Software Version 1.85; Version 2.6.3 or Version 2.7.6

1. INTRODUCTION/BACKGROUND

1.1. PURPOSE/SCOPE

The purpose of this protocol is to provide detailed information for data analysis and data export of mouse STR profiles using GeneMarker Software.

1.2. THIS PROTOCOL PROVIDES DETAILED INFORMATION REGARDING:

1.2.1. Importing of bins and panels, analysis methods, table settings, and size standards for the SoftGenetics GeneMarker® Software Version 1.85; Version 2.6.3 and Version 2.7.6 (files will be provided electronically by NIST).

1.2.2. Adjustment of bins and panels to the appropriate fragment lengths based on data collected using the provided calibrant samples.

1.2.3. Export data file after analysis is complete.

1.3. EACH CONSORTIUM MEMBER WILL BE PROVIDED WITH:

1.3.1. Five calibrant DNA samples

1.3.1.1. Calibrants 1-4 contain mixtures of 2 or more mouse DNAs to obtain the most allele coverage possible; Calibrant 5 is a single sample.

1.3.1.2. Calibrants will be used to generate fragment lengths obtained by genotyping the samples using the mouse multiplex PCR assay.
Note: All alleles present in the calibrant samples have been sequenced.

1.3.2. STR profiles for the calibrant DNA (Table 1) and their associated electropherograms (Figures 1-5).

1.3.2.1. These profiles and fragment lengths were obtained in NIST laboratories using an ABI 3500xl, POP-4 polymer, and a 36cm array.

1.3.2.2. Fragment length values may vary if instruments and consumables are used other than what is listed above.

1.3.3. An Excel file of a fill-in table (Table 2) containing all known alleles for 19 mouse STR markers.

1.3.3.1. Fragment lengths obtained from genotyping the calibrant samples using the mouse multiplex PCR assay will be recorded in Table 2.

1.3.3.2. Dataset from the calibrants will be used to adjust the bins and panels.

2. DOCUMENTS/RECORDS

Document any changes made in the protocol with an explanation and submit this information along with the analyzed data.

3. EQUIPMENT/SUPPLIES

3.1. Computer with Windows Software

3.2. GeneMarker® Software (one of the following software)

3.2.1. GeneMarker® Software Version 1.85.

3.2.2. GeneMarker® Software Version 2.6.3.

3.2.3. GeneMarker® Software Version 2.7.6.

4. PROTOCOL

4.1. IMPORTING BINS AND PANELS

4.1.1 Download the files for bins/panels, provided by NIST, to a desktop with GeneMarker Software.

4.1.2. Open GeneMarker Software and "Log in".

4.1.3. In the toolbar, select "Tools" and then select "Panel Editor" from the drop-down menu.

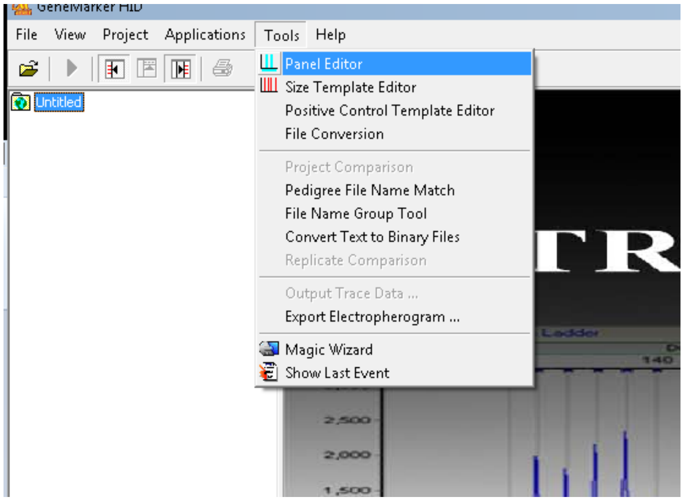

4.1.4. Select “File” and then “Import Panels” (which includes both bins and panels).

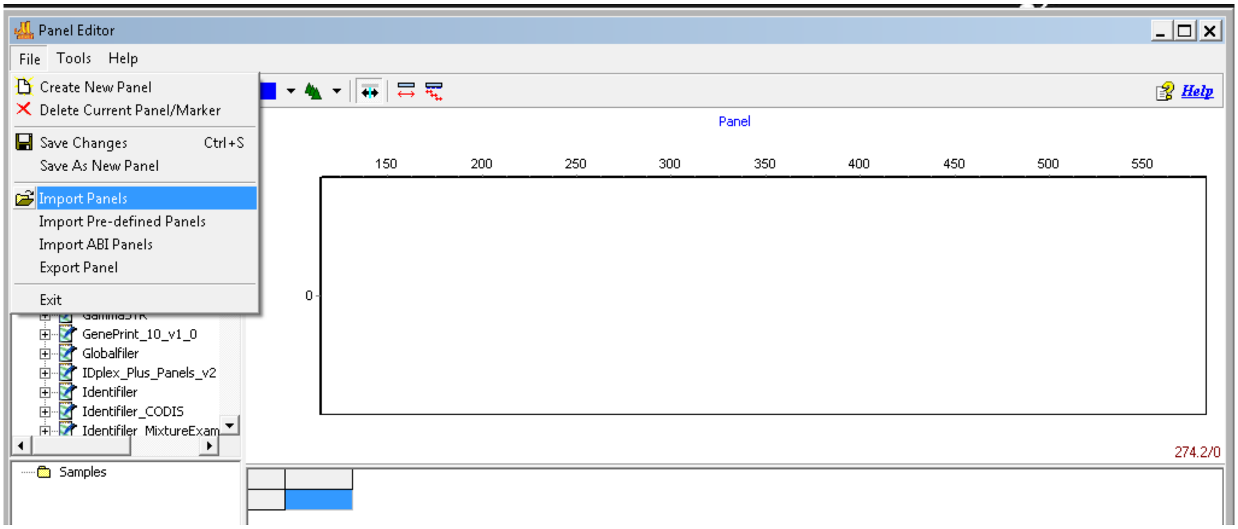

4.1.5. Navigate to find the files provided by NIST on the desktop (12plex_100rfu and 12plex_500rfu) and click “Open”. Both files can be imported at the same time.

4.1.6. Under “Panel Templates” you should now see 21plex_100rfu and 21plex_500rfu. Save and close the window.

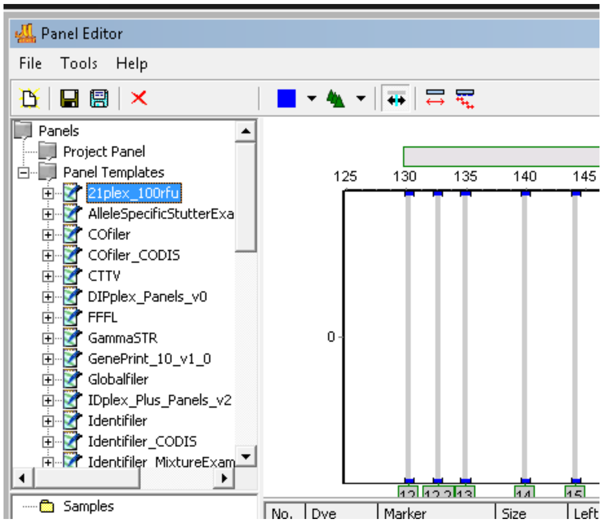

4.2. ADJUSTING OF BINS AND PANELS

Different instruments, DNA size standards, polymers, and arrays may result in different fragment lengths of the alleles (size in base pairs). The current bins and panels reflect fragment lengths using the ABI 3500xl, POP-4 polymer, and a 36cm array in our lab. Calibrant samples are provided with known repeats based

on sequence data. The bins and panels may need to be adjusted to reflect differences in fragment length based on instrumentation other than what is listed above.

4.2.1. **STR analysis of five calibrants**

4.2.1.1. Complete PCR and fragment analysis of the five calibrant DNA samples by following Protocol 1: PCR and Genetic Analyzer Sample Preparation.

4.2.1.2. Transfer data from the Genetic Analyzer onto a desktop with GeneMarker Software. Go to "File" and then "Open Data".

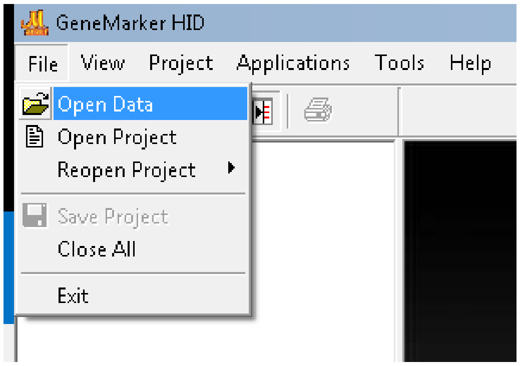

4.2.1.3. Select "Add Folder" and select the data file from the Genetic Analyzer and click "OK".

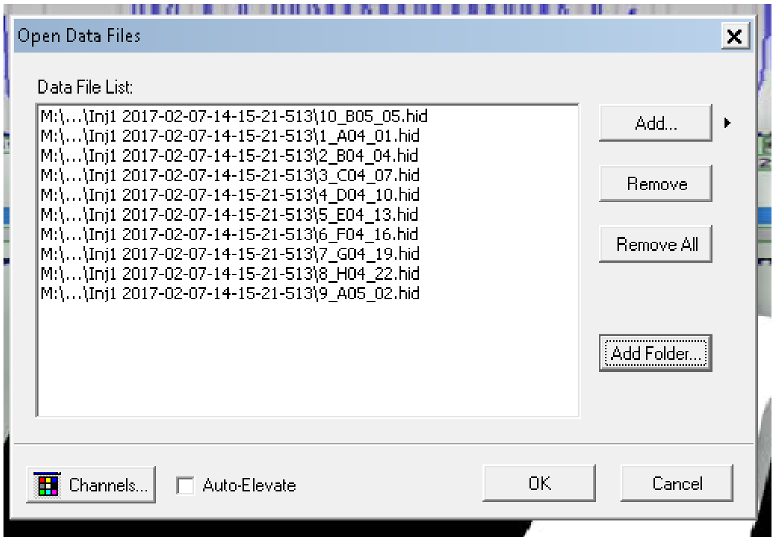

4.2.1.4. The data files will be visible. Select "OK". The raw data files should now be visible.

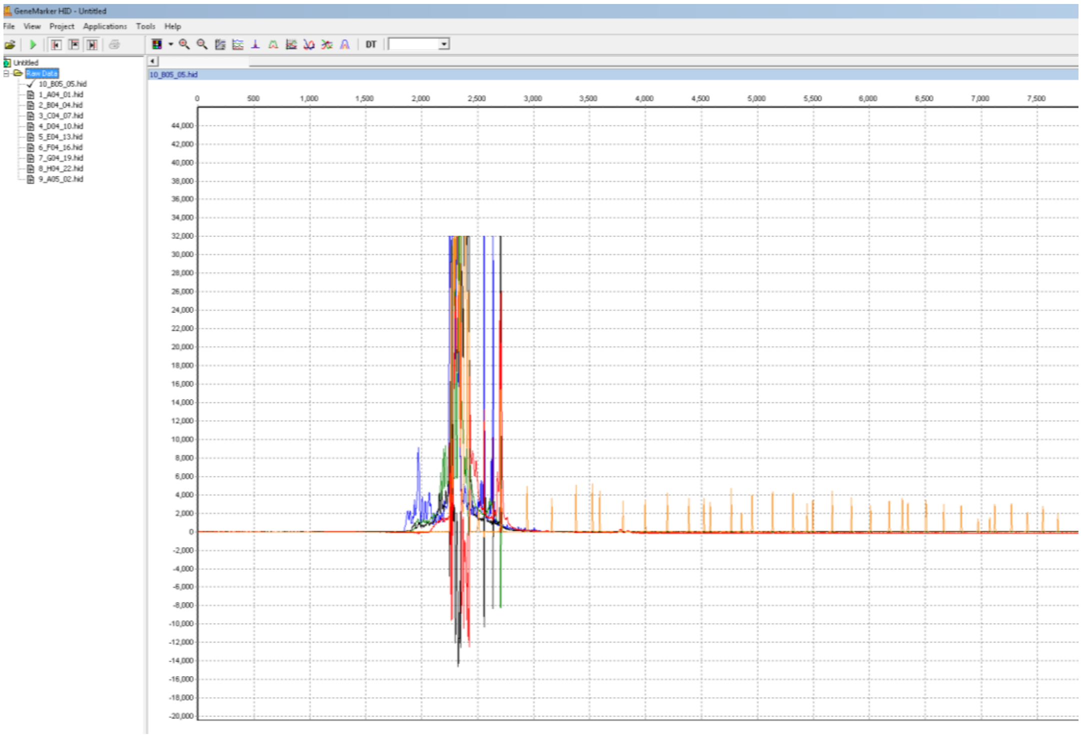

4.2.1.5. Go to “Project” and select “Run”.

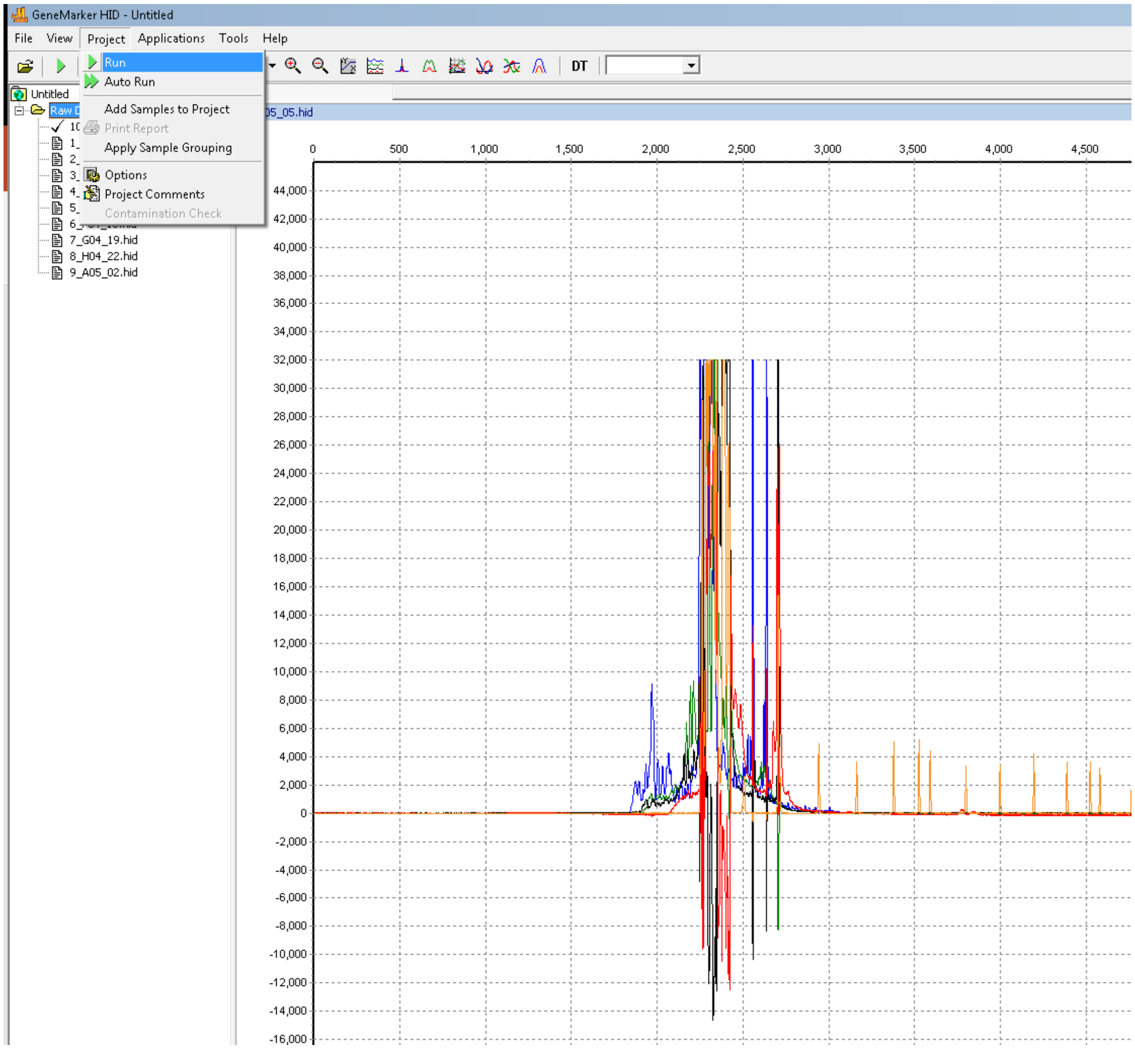

4.2.1.6. Create a Run Template. The "Run Wizard" appears. Click "Select an existing template or create one". Within "Template Name" type in "Mouse 21". Choose one of the panels that were just imported (ex. 21plex_500rfu), select the size standard (GS600_LIZ), select the Standard Color as "LIZ", and choose Analysis Type (based on file structure: .hid, .fsa, etc.) and then click "Save". Click "Next".

NOTE: After the template has been saved, the template name will appear in the left column.

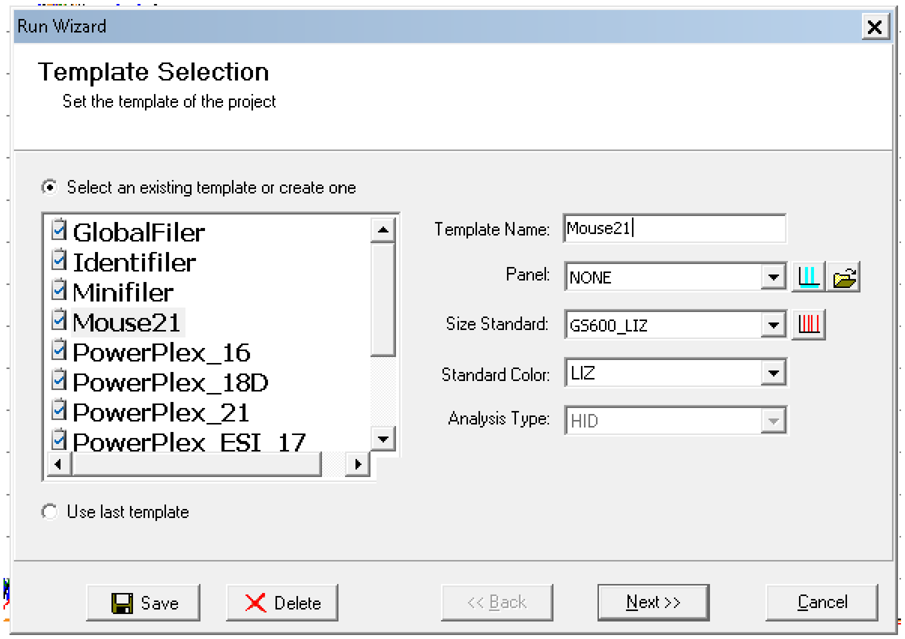

4.2.1.7. The “Data Process-HID Analysis” screen should appear with default settings. Click “Next”.

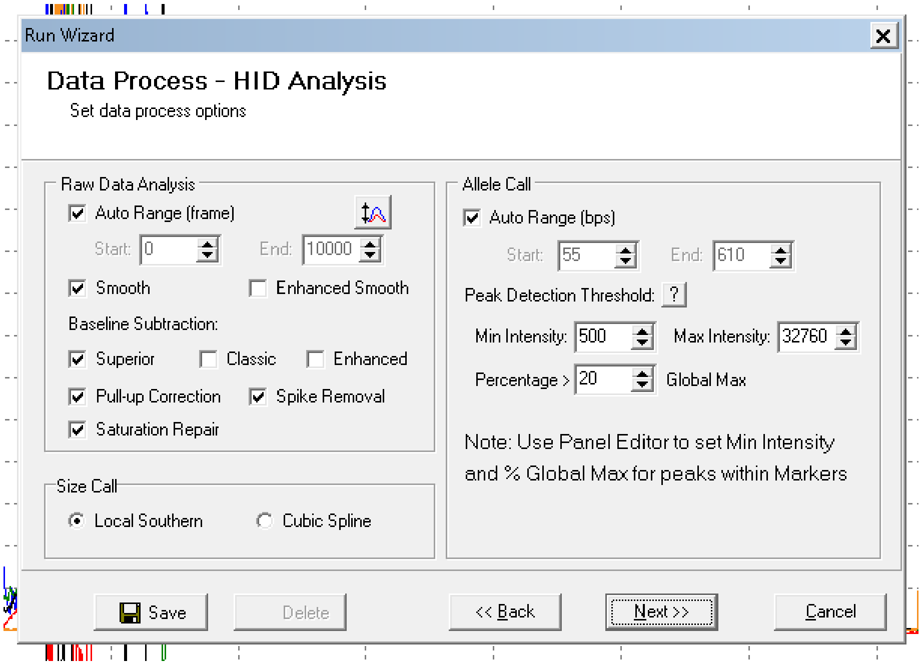

4.2.1.8. On the “Additional Settings-HID Analysis” screen, use default settings but deselect “Auto Select Best Ladder” and “Auto Panel Adjustment” on the far right. Click “Save” and then click “OK”.

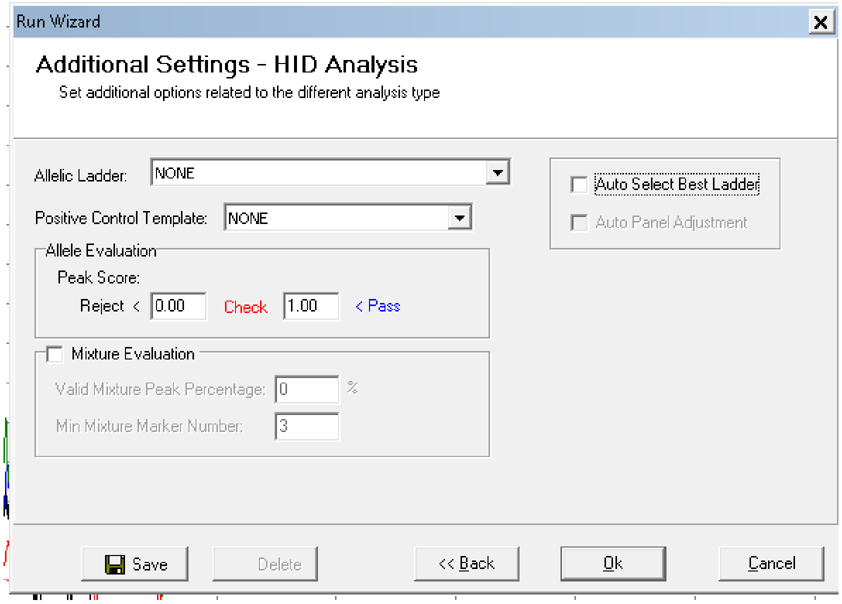

4.2.1.9. Data is now being processed. Click “OK”.

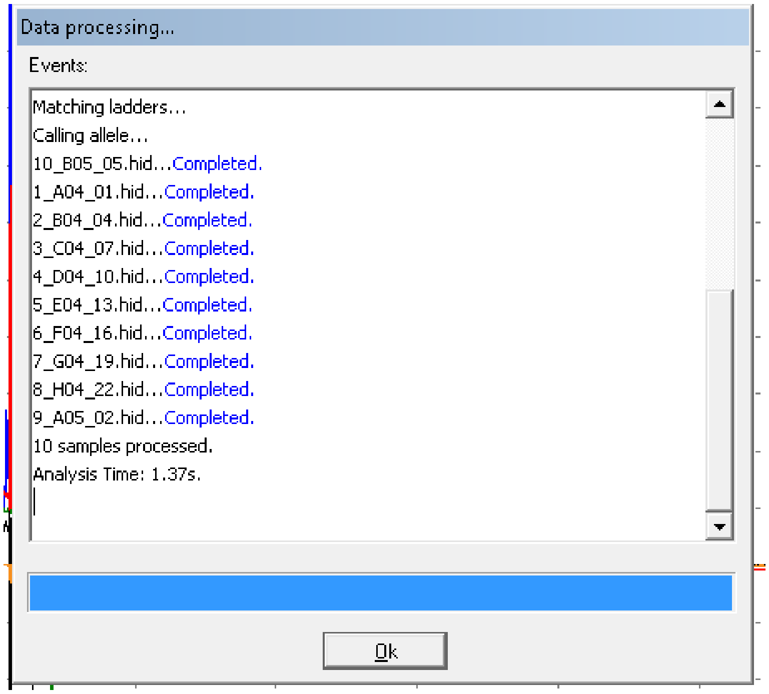

4.2.1.10. Size Standard screen is now visible. If the size standard is flagged, check the quality of the size standard. This may be due to a poor injection or blown out signal. Reinject the sample if the problem is due to a bad injection. If the sample is blown out and has bleed through, check the sizing peaks (these can be overridden if the peaks look ok). The size standard data can be accessed by selecting “Tools” and “Size Template Editor” from the drop-down menu.

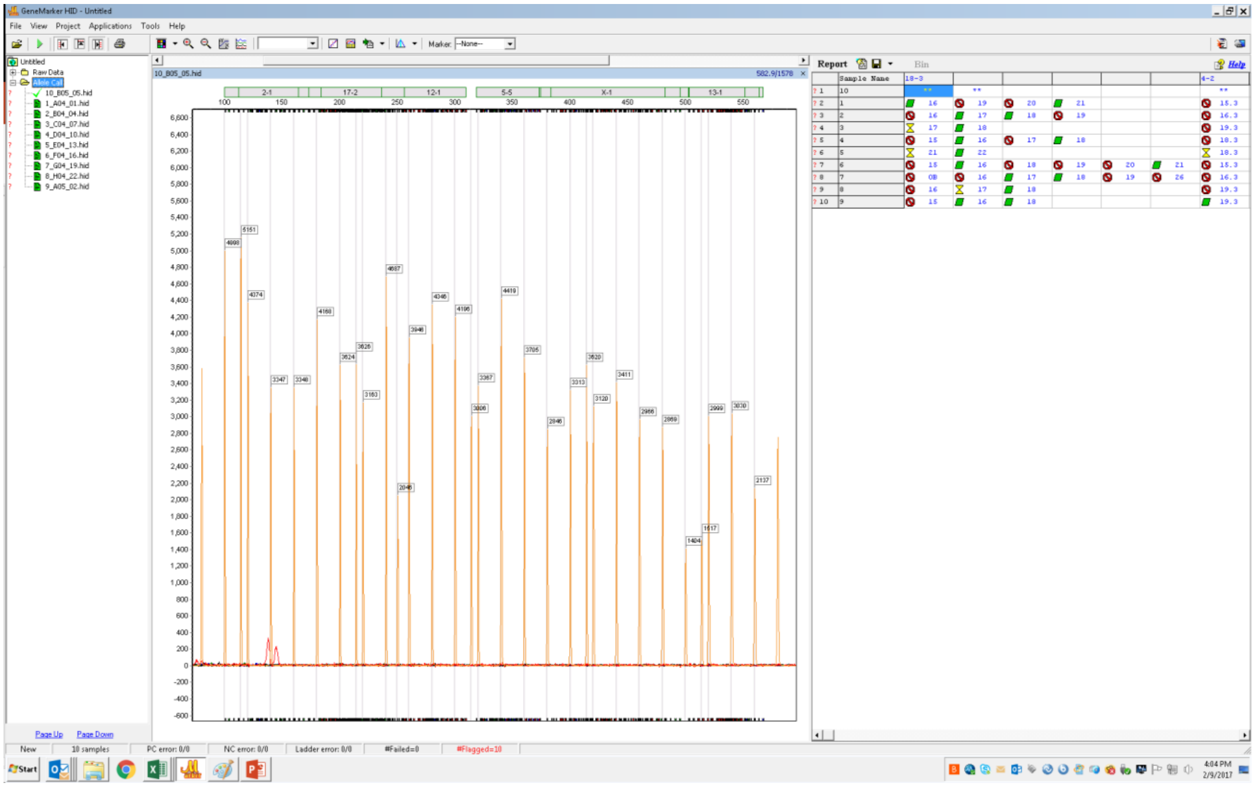

NOTE: To visualize the split screen to see data, go to "View" and select "Show Report" and "Show Navigator".

4.2.2. Calibrant Data Import

4.2.2.1. Complete Table 2 (Allele Distribution for 19 STR Markers) by filling in the fragment length data obtained for each allele at each STR marker using the calibrant samples.

4.2.2.2. Select a single dye channel (blue for example) and zoom in to see the allele calls for STR marker 18-3. Record the fragment lengths for each calibrant for 18-3. If an allele is not unlabeled and it should be, right click on the peak and select "Insert allele". Adjust the allele call if it is incorrect. Click "OK".

4.2.2.3. Extrapolate fragment length values for alleles not represented in the calibrant samples until Table 2 is completed.

Example: 18-3

Allele Distribution for 19 STR Markers															
Marker	Alleles														
18-3															
FR3pig															
repeat	12	12.2	13	14	15	16	17	18	19	20	21	22	23	26	27

For example: STR analysis of five calibrants will cover six alleles (repeats) for 18-3 Marker: 16, 17, 18, 19, 21 and 22. Allele 20 is unrepresented. The fragment length for the allele 20 will be estimated by extrapolating from the fragment length of allele 19 (fragment length: 160.8 bp) or 21 (168.8). Since each allele number represents tetranucleotide repeats, one allele will differ from the next one by 4 bp, and the 20 will be estimated to have 164.8 bp. Therefore, the fragment length of the allele 20 will be 4 bp more from allele 19 and 4 bp less than allele 21.

For microvariants (ex. 20.1) the fragment length would be extrapolated by one base more than the fragment length for allele 20 and it will be 165.8 bp. ***Note: The sizing may vary slightly (165.8 bp may be 165.6 bp) due to migration effects.*** The left and right bin offset is set to 0.4 bp of the mean fragment length for each bin and should capture these slight variations in fragment length.

No. Repeats	Fragment Length (bp)
20	164.8
20.1	165.8
20.2	166.8
20.3	167.8
21	168.8
21.1	169.8

4.2.2.4. Once Table 2 is complete, adjust all bins and panels to the fragment lengths recorded in Table 2 (see instructions below).

4.2.3. Adjusting bins and panels

4.2.3.1. Go to "Tools", select "Panel Editor", and click on the "Panel Templates". Select "21plex_100rfu" (or "21plex_500rfu).

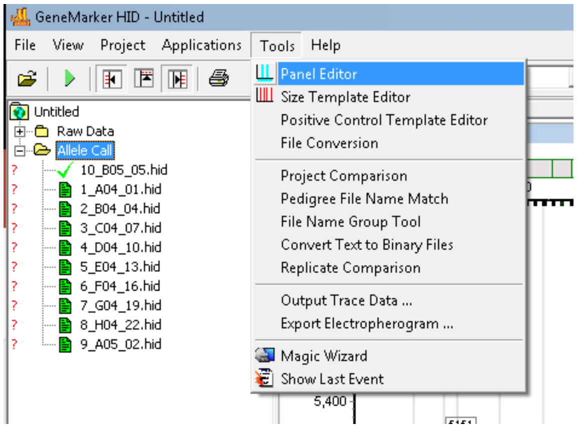

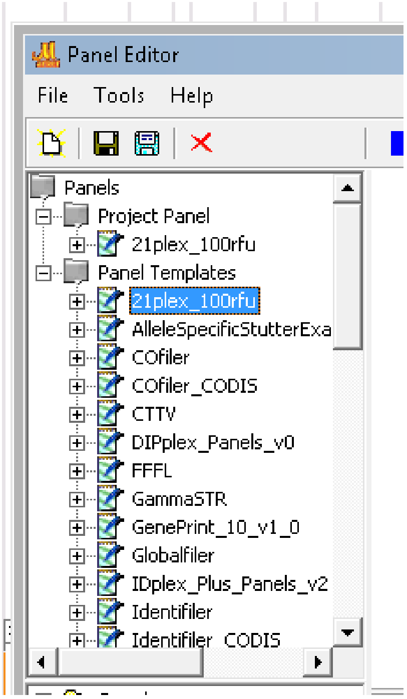

4.2.3.2. Expand 21plex_100rfu to see all of the STR markers.

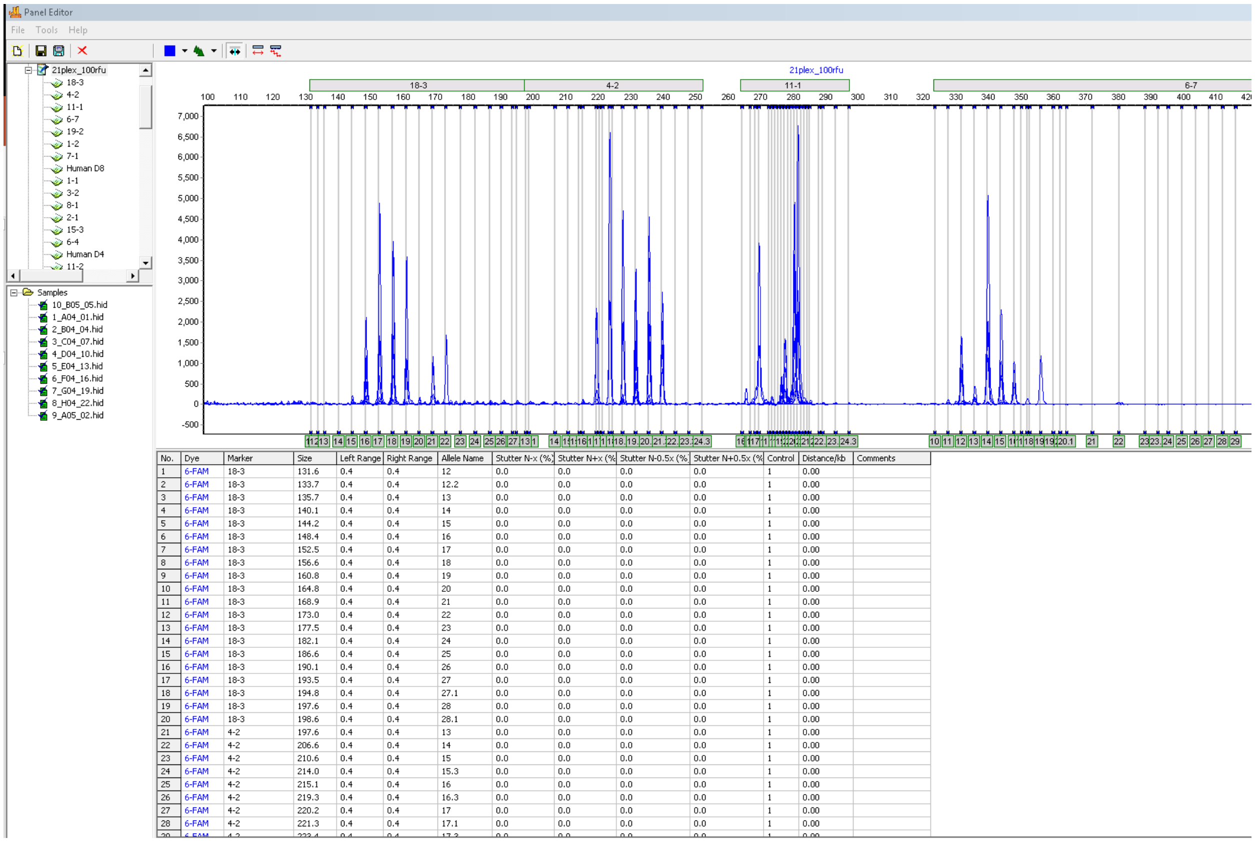

4.2.3.3. Click on marker 18-3 to visualize the calibrant allele calls and additional bins. Fill in the extrapolated values from Table 2 for 18-3 in the lower panel of the screen by clicking on a cell under the “Size” column. Leave the “Left Range” and “Right Range” at 0.4. Do not change the marker or the allele name. Adjust for each fragment length for each allele based on the calibrant sizing. ***NOTE: Do not use Auto Adjust. We found it to be inaccurate.***

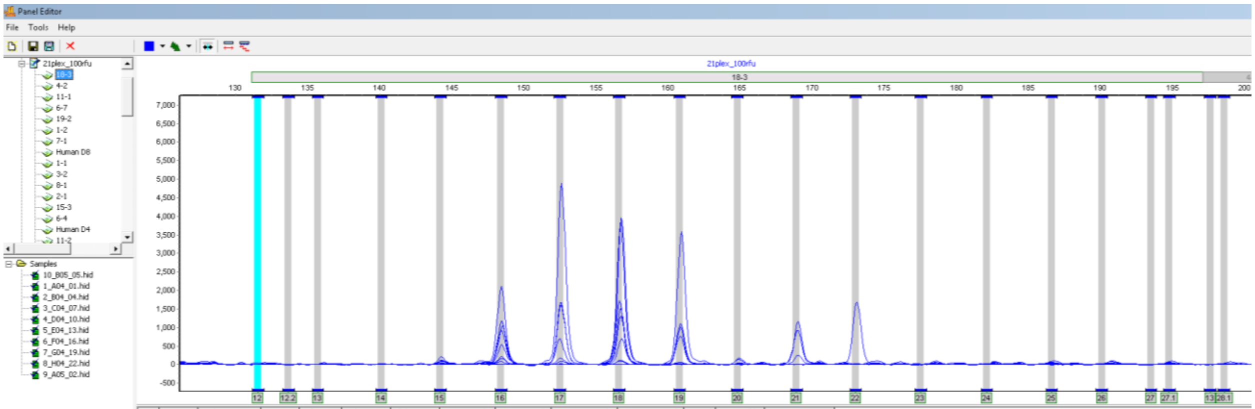

No.	Dye	Marker	Size	Left Range	Right Range	Allele Name	Stutter N-x (%)	Stutter N+x (%	Stutter N-0.5x (%	Stutter N+0.5x (%	Control	Distance/kb	Comments
1	6-FAM	18-3	131.6	0.4	0.4	12	0.0	0.0	0.0	0.0	1	0.00	
2	6-FAM	18-3	133.7	0.4	0.4	12.2	0.0	0.0	0.0	0.0	1	0.00	
3	6-FAM	18-3	135.7	0.4	0.4	13	0.0	0.0	0.0	0.0	1	0.00	
4	6-FAM	18-3	140.1	0.4	0.4	14	0.0	0.0	0.0	0.0	1	0.00	
5	6-FAM	18-3	144.2	0.4	0.4	15	0.0	0.0	0.0	0.0	1	0.00	
6	6-FAM	18-3	148.4	0.4	0.4	16	0.0	0.0	0.0	0.0	1	0.00	
7	6-FAM	18-3	152.5	0.4	0.4	17	0.0	0.0	0.0	0.0	1	0.00	
8	6-FAM	18-3	156.6	0.4	0.4	18	0.0	0.0	0.0	0.0	1	0.00	
9	6-FAM	18-3	160.8	0.4	0.4	19	0.0	0.0	0.0	0.0	1	0.00	
10	6-FAM	18-3	164.8	0.4	0.4	20	0.0	0.0	0.0	0.0	1	0.00	
11	6-FAM	18-3	168.9	0.4	0.4	21	0.0	0.0	0.0	0.0	1	0.00	
12	6-FAM	18-3	173.0	0.4	0.4	22	0.0	0.0	0.0	0.0	1	0.00	
13	6-FAM	18-3	177.5	0.4	0.4	23	0.0	0.0	0.0	0.0	1	0.00	
14	6-FAM	18-3	182.1	0.4	0.4	24	0.0	0.0	0.0	0.0	1	0.00	
15	6-FAM	18-3	186.6	0.4	0.4	25	0.0	0.0	0.0	0.0	1	0.00	
16	6-FAM	18-3	190.1	0.4	0.4	26	0.0	0.0	0.0	0.0	1	0.00	
17	6-FAM	18-3	193.5	0.4	0.4	27	0.0	0.0	0.0	0.0	1	0.00	
18	6-FAM	18-3	194.8	0.4	0.4	27.1	0.0	0.0	0.0	0.0	1	0.00	
19	6-FAM	18-3	197.6	0.4	0.4	28	0.0	0.0	0.0	0.0	1	0.00	
20	6-FAM	18-3	198.6	0.4	0.4	28.1	0.0	0.0	0.0	0.0	1	0.00	

4.2.3.4. Click on the light blue save icon “Save changes with signal info” first.

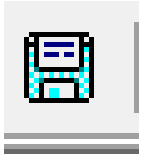

4.2.3.5. Click on “Save changes” icon.

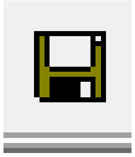

4.2.3.6. Repeat the same procedure to adjust bins for every allele represented on Table 2.

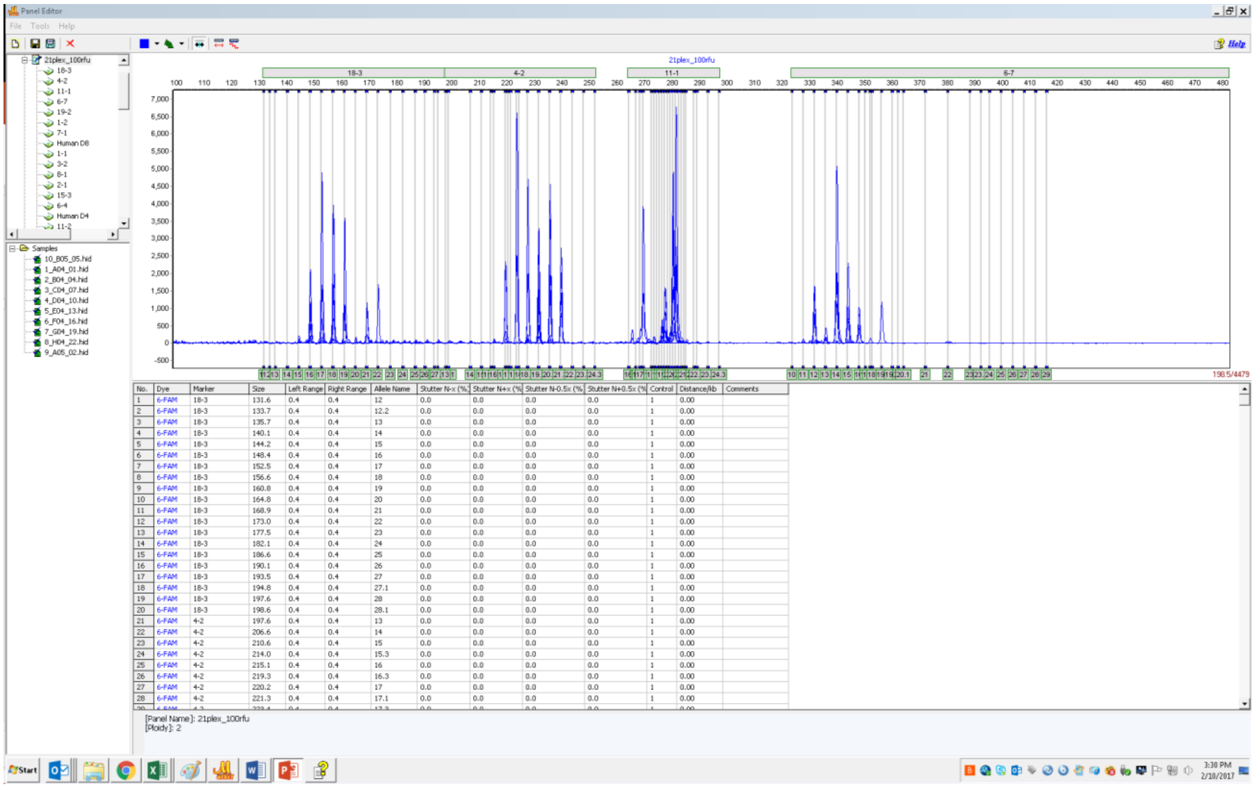

4.2.3.7. Click on the light blue save icon “Save changes with signal info” first.

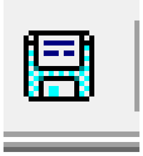

4.2.3.8. Click on “Save changes” icon.

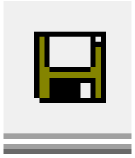

4.2.3.9. After all bins have been adjusted and saved, reanalyze samples and confirm that all alleles fall into bins. If there is an off ladder call (OL) adjust that particular bin to accommodate the fragment length that maybe off by 0.1 bp. The bin offset can also be expanded to 0.50 instead of 0.40 for the left and right to help with this situation.

4.3 EXPORTING DATA FILE

To export the data table:

4.3.1. After analysis of all samples is complete, ensure that the report tab is open (Go to View and select "Show Report").

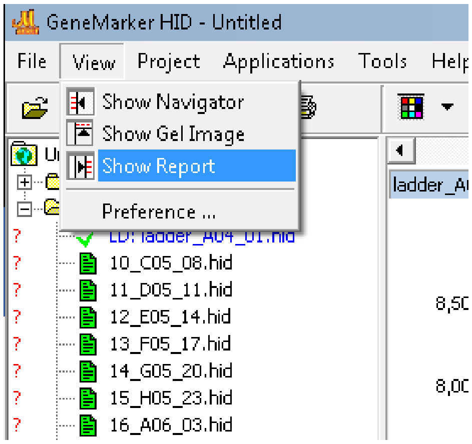

4.3.2. Select "Report Settings" on the Report table view.

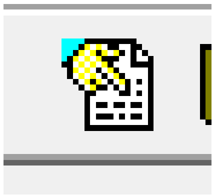

4.3.3. Under "Report Style" select "Peak Table".

4.3.4. Ensure that "Show Sample Name" is selected and Under "Options" make sure "Size Range" is de-selected.

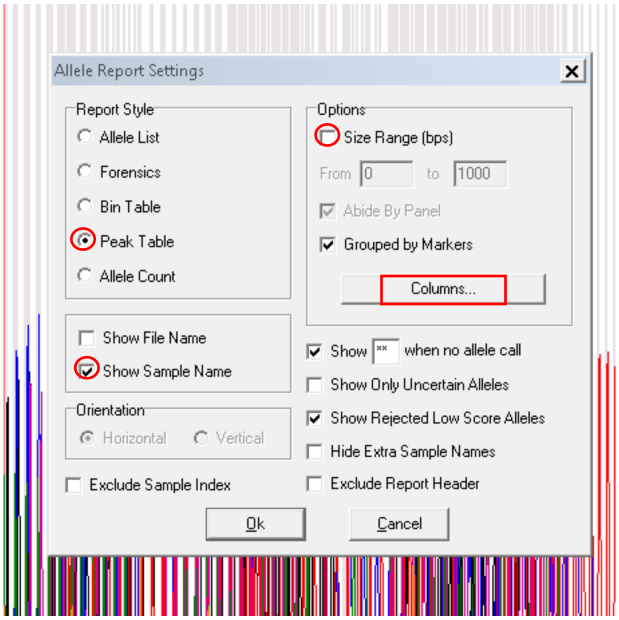

4.3.5. In the "Allele Report Settings" click on "Columns" and add "Size", "Height", "Marker", and "Allele" to the Selected Columns. Click "OK", and then click "OK" again.

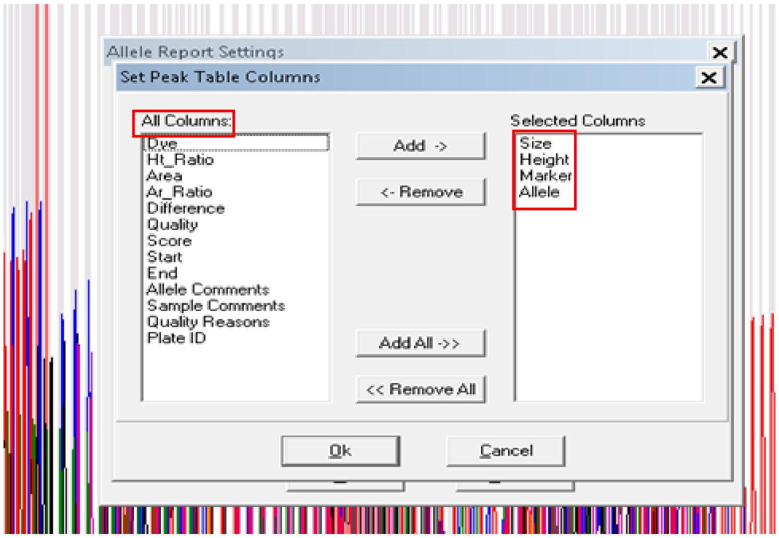

4.3.6. Click on the icon symbol shown below to ensure that all dyes are selected.

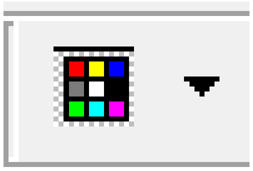

4.3.7. Save the file with the following convention:
NIST Mouse Consortium_Institution_date. It will save as a .xls file.

4.3.8. A notification of a successfully saved file should appear.

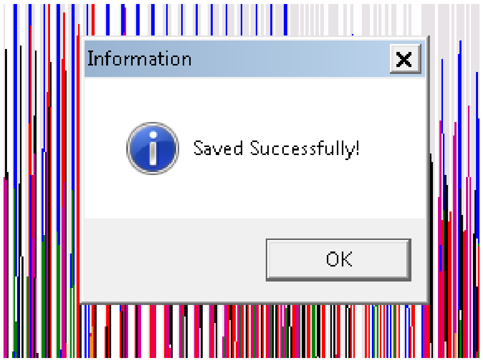

4.3.9. Send data file (.xls) to NIST for analysis
