# Supplementary figures and images for: Interlaboratory study to validate a STR profiling method for intraspecies identification of mouse cell lines
Source: PLoS One. 2019 Jun 20;14(6):e0218412. doi: 10.1371/journal.pone.0218412 (PMC6586308; doi:10.1371/journal.pone.0218412)

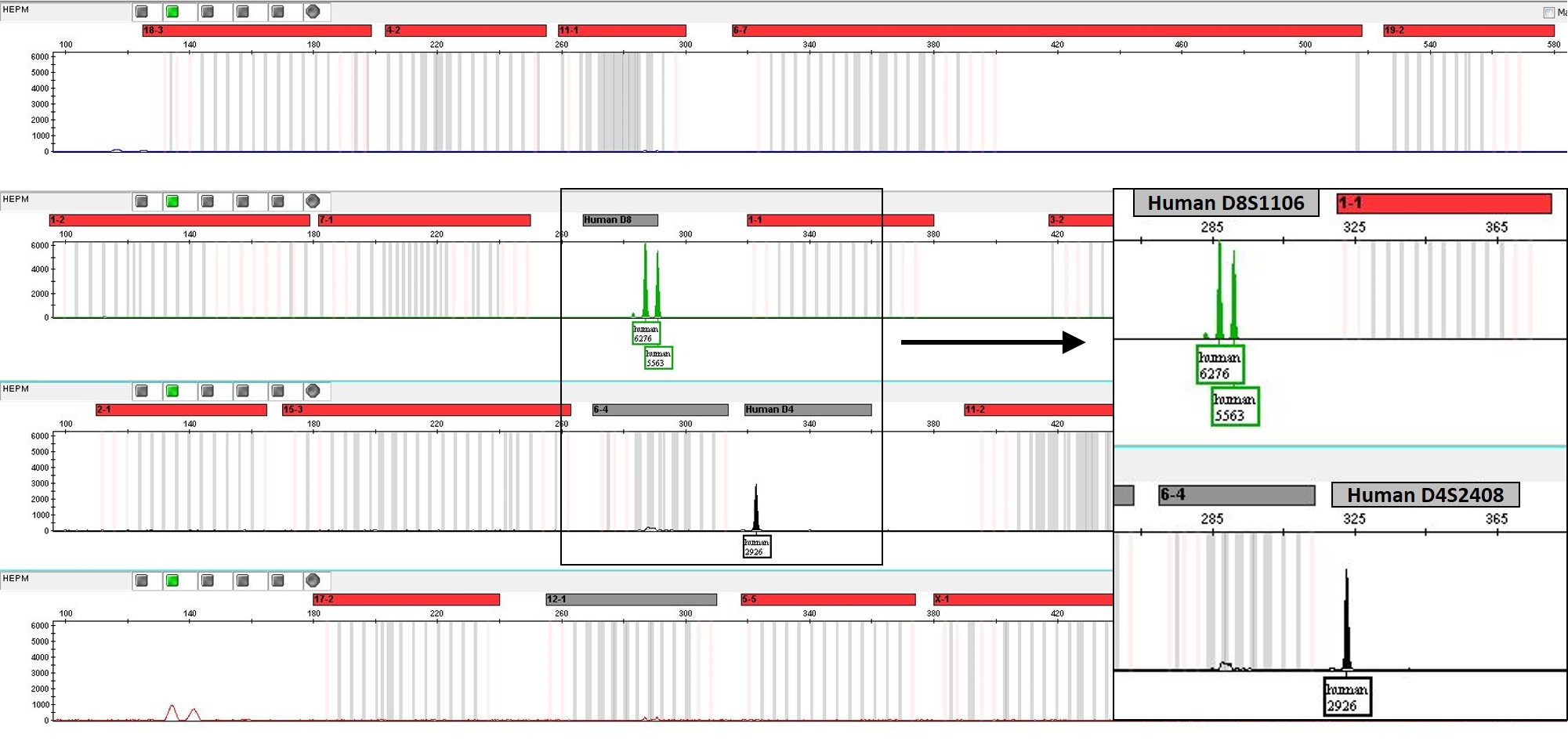

Supplement: S1 Fig — Amplification of human alleles present at D8S1106 and D4S2408 in the HEPM cell line using the multiplex PCR assay targeting 19 mouse STR markers and two human STR markers. (TIF) [file pone.0218412.s012.tif]

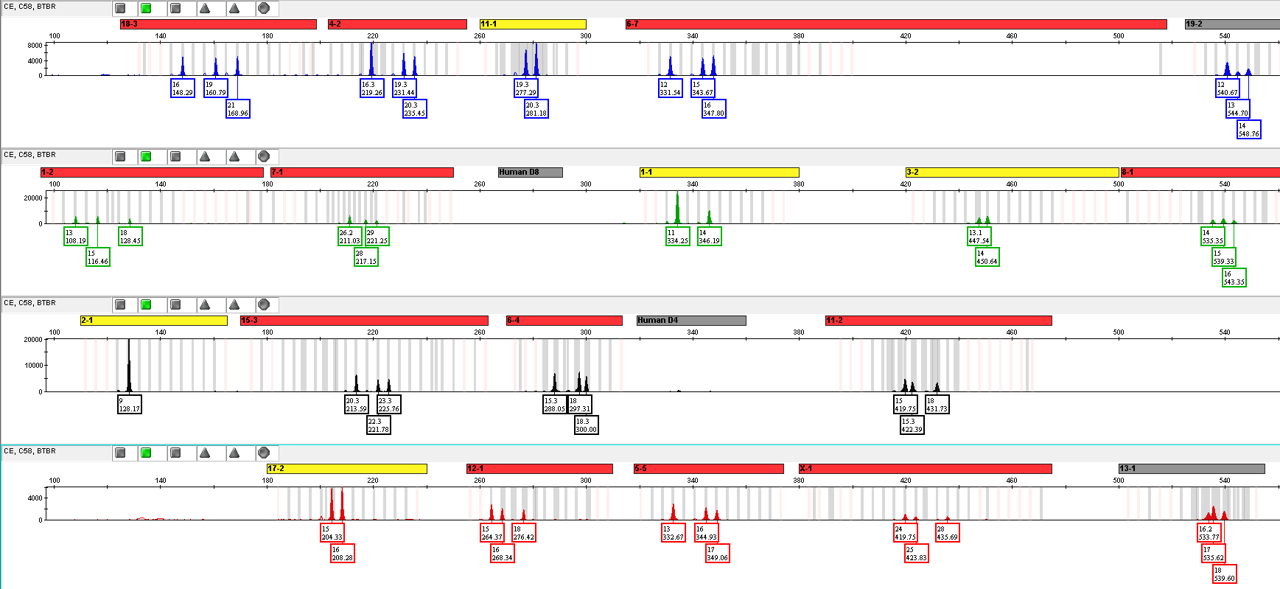

Supplement: S2 Fig — STR profile shown for Calibrant 1, a mixed diploid DNA sample from C58/J, CE/J, and BTBR T<+> tf/J mice, at 19 mouse STR markers. (TIF) [file pone.0218412.s013.tif]

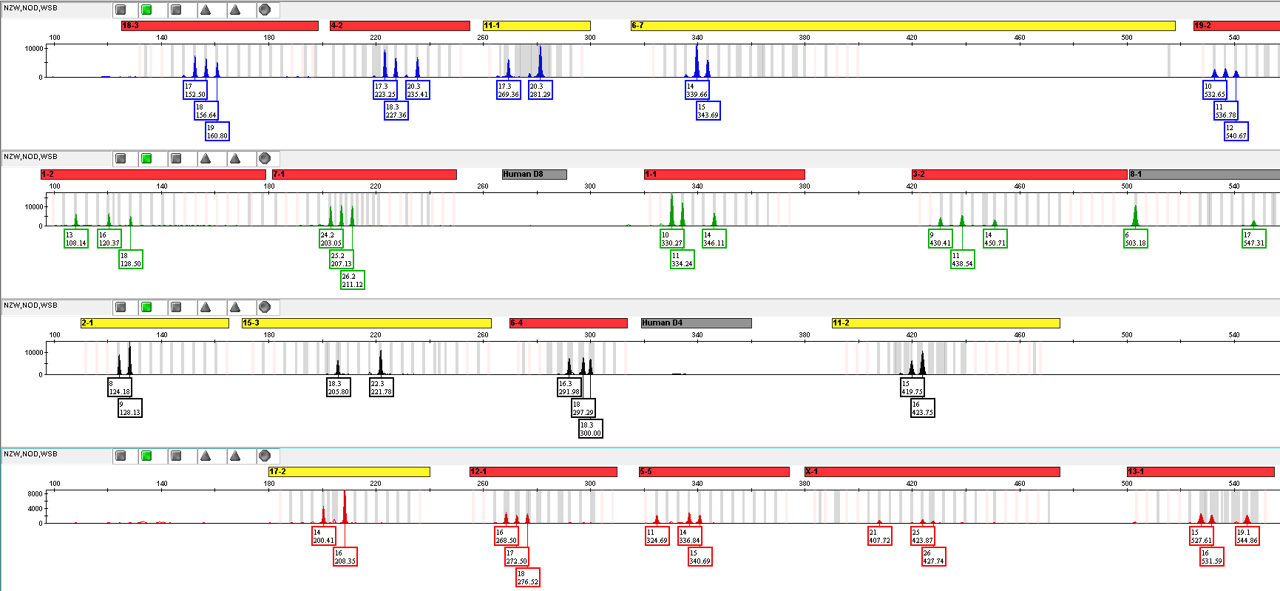

Supplement: S3 Fig — STR profile shown for Calibrant 2, a mixed diploid DNA sample from NZW/LacJ, NOD/ShiLtJ, and WSB/EiJ mice, at 19 mouse STR markers. (TIF) [file pone.0218412.s014.tif]

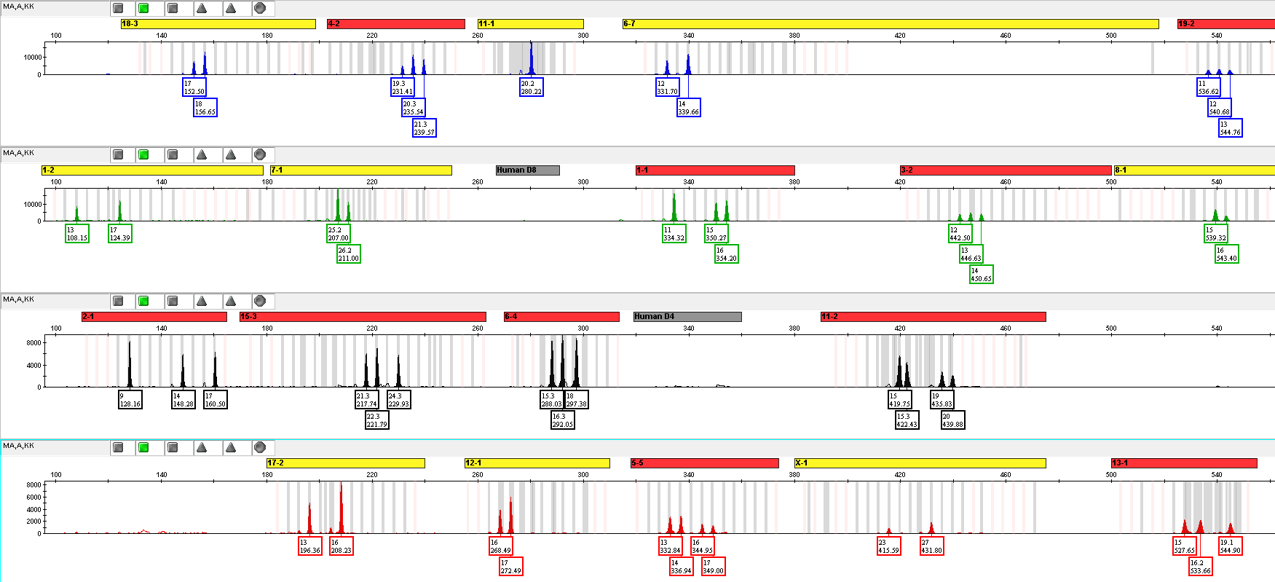

Supplement: S4 Fig — STR profile shown for Calibrant 3, a mixed diploid DNA sample from A/J, KK/HlJ, and MA/MyJ mice, at 19 mouse STR markers. (TIF) [file pone.0218412.s015.tif]

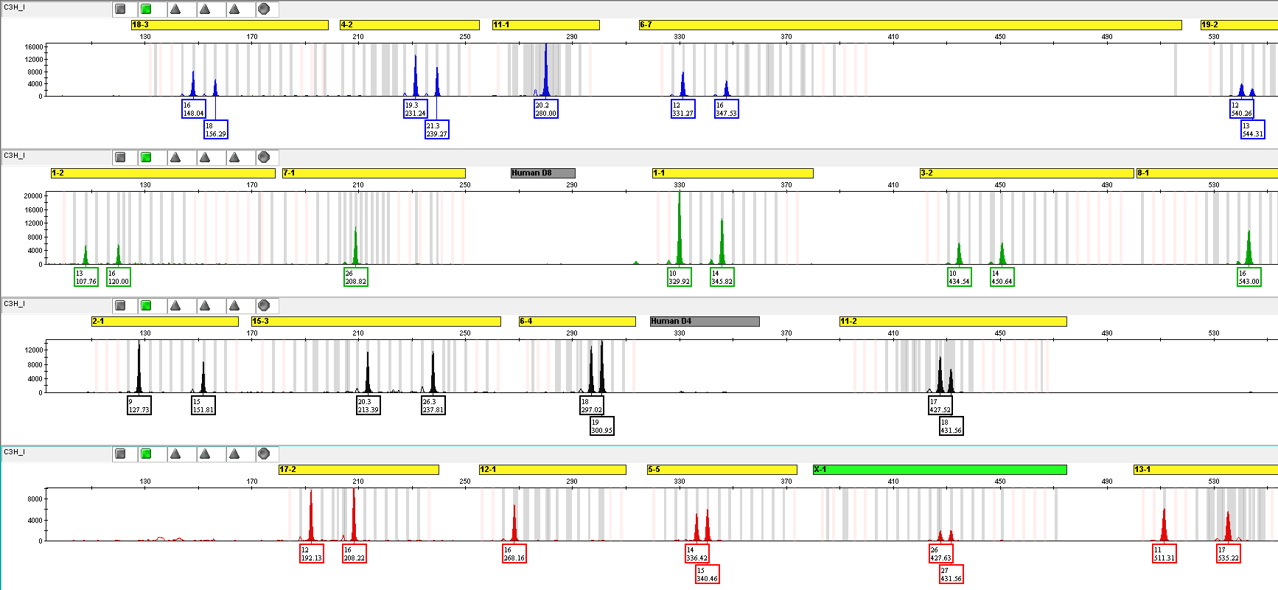

Supplement: S5 Fig — STR profile shown for Calibrant 4, a mixed diploid DNA sample from C3H/HeJ and I/LnJ mice, at 19 mouse STR markers. (TIF) [file pone.0218412.s016.tif]

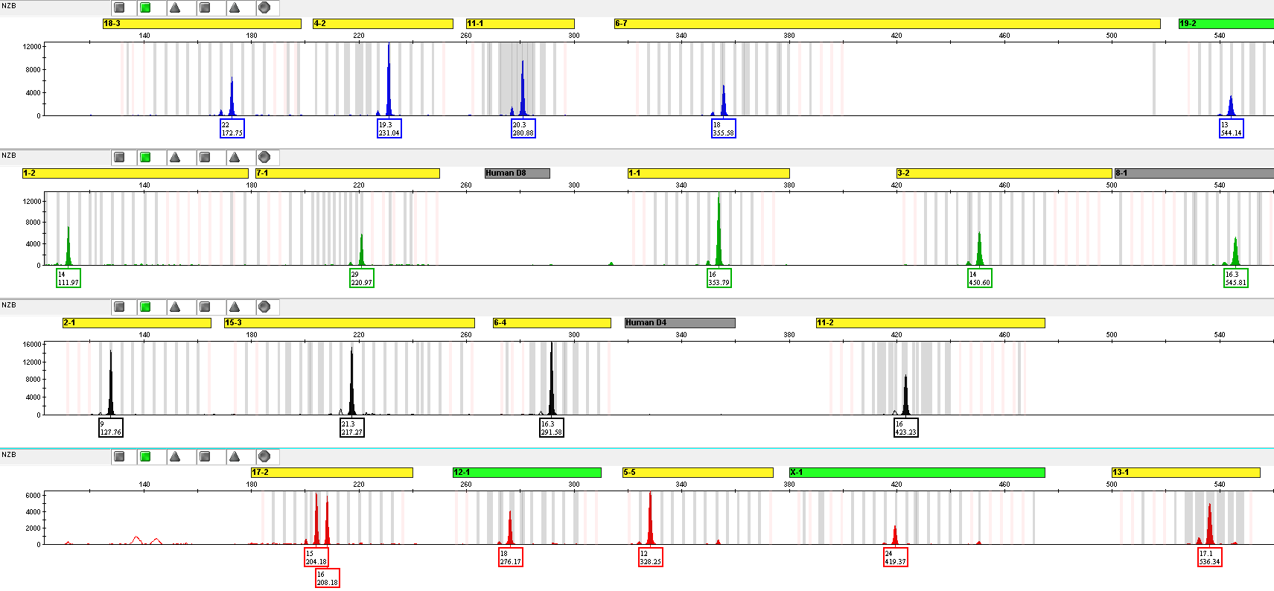

Supplement: S6 Fig — STR profile shown for Calibrant 5, a diploid DNA sample from an NZB/BlNJ mouse, at 19 mouse STR markers. (TIF) [file pone.0218412.s017.tif]

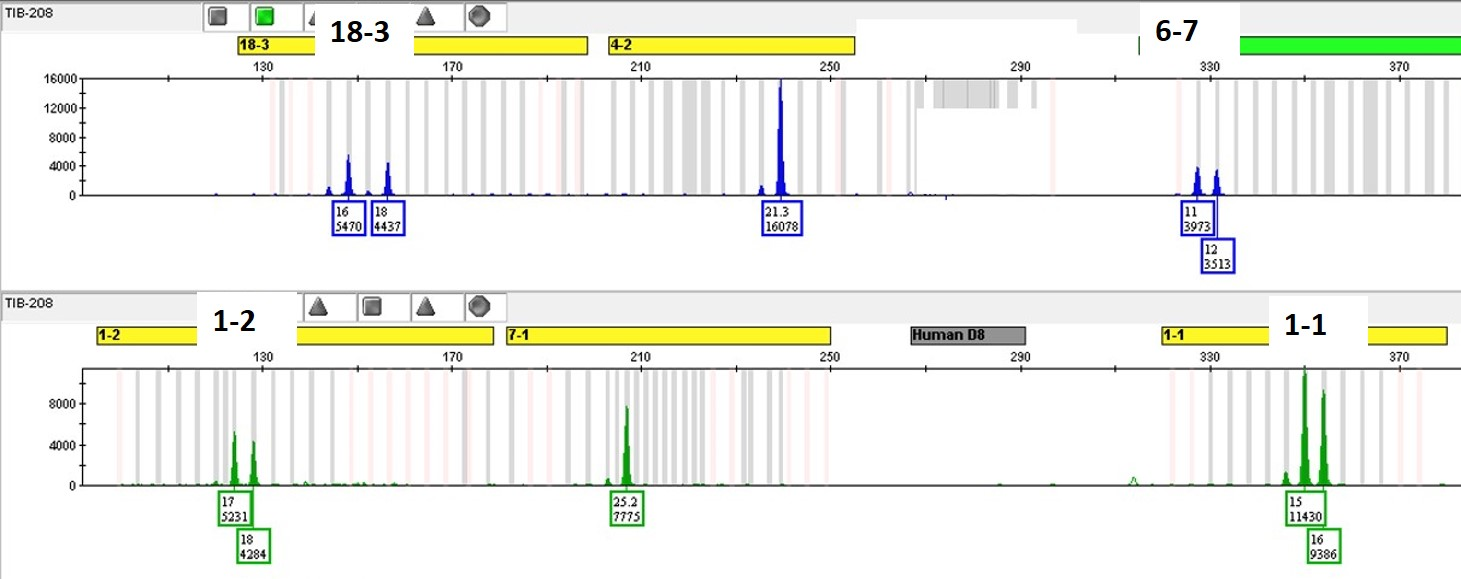

Supplement: S7 Fig — A20, a sarcoma cell line, is an example of a cell line that has balanced peak height at STR markers 18–3, 6–7, 1–2, and 1–1. (TIF) [file pone.0218412.s018.tif]

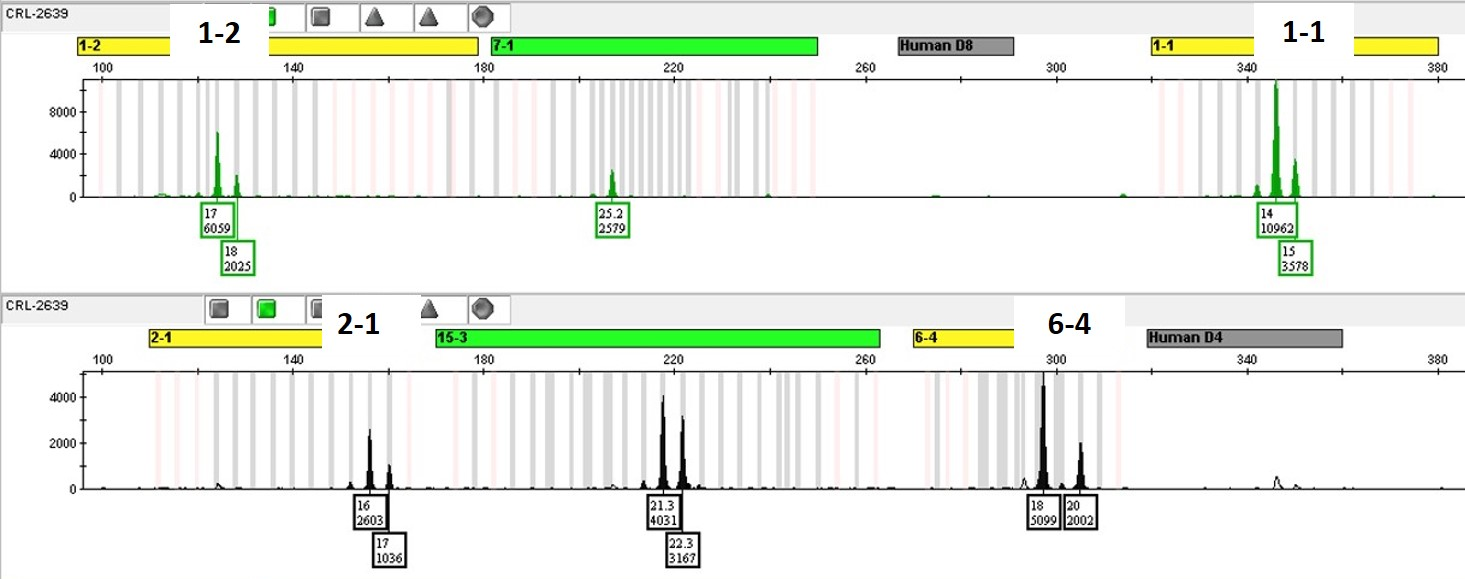

Supplement: S8 Fig — A colon carcinoma cell line, CT26.CL25, has several instances of peak height imbalance shown at STR markers 1–2, 1–1, 2–1, and 6–4. (TIF) [file pone.0218412.s019.tif]
